# G_i/o_-Protein Coupled Receptors in the Aging Brain

**DOI:** 10.3389/fnagi.2019.00089

**Published:** 2019-04-24

**Authors:** Patrícia G. de Oliveira, Marta L. S. Ramos, António J. Amaro, Roberto A. Dias, Sandra I. Vieira

**Affiliations:** ^1^Department of Medical Sciences, Institute of Biomedicine (iBiMED) and The Discovery CTR, Universidade de Aveiro, Aveiro, Portugal; ^2^School of Health Sciences (ESSUA), Universidade de Aveiro, Aveiro, Portugal

**Keywords:** G protein-coupled receptors GPCRs, G_i/o_ heterotrimeric G proteins, aging, receptor density and binding potential, frontal cortex, hippocampus, basal ganglia

## Abstract

Cells translate extracellular signals to regulate processes such as differentiation, metabolism and proliferation, via transmembranar receptors. G protein-coupled receptors (GPCRs) belong to the largest family of transmembrane receptors, with over 800 members in the human species. Given the variety of key physiological functions regulated by GPCRs, these are main targets of existing drugs. During normal aging, alterations in the expression and activity of GPCRs have been observed. The central nervous system (CNS) is particularly affected by these alterations, which results in decreased brain functions, impaired neuroregeneration, and increased vulnerability to neuropathologies, such as Alzheimer’s and Parkinson diseases. GPCRs signal via heterotrimeric G proteins, such as G_o_, the most abundant heterotrimeric G protein in CNS. We here review age-induced effects of GPCR signaling via the G_i/o_ subfamily at the CNS. During the aging process, a reduction in protein density is observed for almost half of the G_i/o_-coupled GPCRs, particularly in age-vulnerable regions such as the frontal cortex, hippocampus, substantia nigra and striatum. G_i/o_ levels also tend to decrease with aging, particularly in regions such as the frontal cortex. Alterations in the expression and activity of GPCRs and coupled G proteins result from altered proteostasis, peroxidation of membranar lipids and age-associated neuronal degeneration and death, and have impact on aging hallmarks and age-related neuropathologies. Further, due to oligomerization of GPCRs at the membrane and their cooperative signaling, down-regulation of a specific G_i/o_-coupled GPCR may affect signaling and drug targeting of other types/subtypes of GPCRs with which it dimerizes. G_i/o_-coupled GPCRs receptorsomes are thus the focus of more effective therapeutic drugs aiming to prevent or revert the decline in brain functions and increased risk of neuropathologies at advanced ages.

## G Protein Coupled Receptors (GPCRs)

**FIGURE 1:**
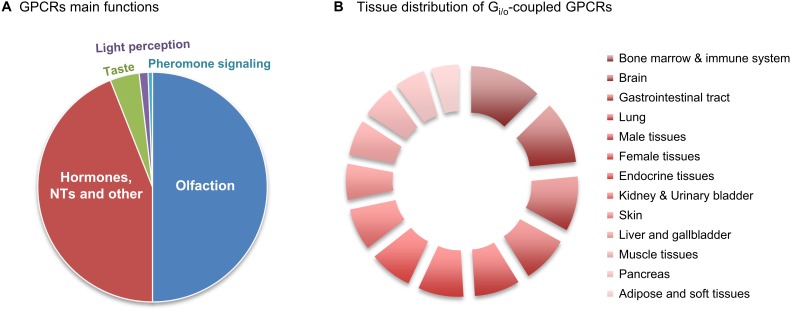
Relative abundance of G_i/o_-coupled GPCRs. **(A)** GPCRs’ main functions. There are about 800 human GPCRs, and around half have sensory functions that mediate olfaction, taste, light perception and pheromone signaling. Another big slice of the pie belongs to non-sensory GPCRs that mediate signaling from other type of ligands, mainly from hormones and neurotransmitters (NTs). **(B)** Relative abundance of G_i/_o-coupled GPCRs in 13 human tissues/regions was taken from the Human Protein Atlas (in Supplementary Table S1), quantitatively transformed (value 1 was attributed to ‘low’, 2.5 for ‘medium’ and 3.5 for ‘high’ abundance), and the relative % of tissue distribution calculated by taking the sum of all abundance values, in all the 13 tissues, as 100%.

G Protein Coupled Receptors (GPCRs) comprise the largest family of transmembrane receptors, with over 800 members present in humans (Fredriksson et al., 2003; Hauser et al., 2017). GPCRs share a common structure of seven transmembrane helical regions, an extracellular N-terminus and an intracellular C-terminus (Alexander et al., 2017). Due to this structure, GPCRs are also called 7-Transmembrane receptors (7-TM receptors). While the first description of a crystalline structure of a non-GPCR 7-TM protein was of the bacteriorhodopsin (Grigorieff et al., 1996), the first GPCR to have its structure determined was bovine retinal rhodopsin (Palczewski et al., 2000). From the more than 800 human GPCRs, around half have sensory functions that mediate olfaction (about 400), taste (33), light perception (10) and pheromone signaling (5) (Mombaerts, 2004). Of the remaining non-sensory GPCRs, which account for over 370, more than 90% are expressed in the brain, and mediate signaling from multiple types type of ligands, regulating several physiological processes throughout the human organism, mainly endocrine and neurological processes (Heng et al., 2013; Rask-Andersen et al., 2014; Munk et al., 2016; Alexander et al., 2017; Huang et al., 2017) (Figure 1A). GPCRs represent the most common target for therapeutic drugs (Hauser et al., 2017). In 2017, Hauser and coworkers by manually curating the Center Watch’s Drugs in Clinical Trials database and by cross referencing with public sources, have identified 475 approved medicine drugs that target GPCRs, which accounted for around 34% of all FDA-approved drugs (Hauser et al., 2017; Sriram and Insel, 2018). This agrees with reports that estimated a proportion ranging from 20 to 50% for drugs that have GPCRs as a target, with discrepancies probably resulting from the varying definitions of “drug target” (Heng et al., 2013; Rask-Andersen et al., 2014; Laschet et al., 2018). As new functions for GPCRs are being discovered, especially for the still around 100 orphan GPCRs for which no endogenous ligand or clearly defined function are currently known, the number of drugs targeting GPCRs is expected to increase (Kroeze et al., 2003; Tuteja, 2009;Laschet et al., 2018).

The first categorization of GPCRs became known as the ‘A-F classification system.’ Created by Kolakowski (1994), it divided GPCRs in six classes/families that share some sequence homology and some functional similarities (Kolakowski, 1994). A seventh class/family (‘O’) was added later on (Tuteja, 2009):

• Family A (rhodopsin receptor family), which includes receptors for odorants, is the largest family of GPCRs and was first divided into three groups (1–3) according to the type of ligand (e.g., small ligands, peptides, high molecular weight hormones such as FSH/TSH/LH) and the GPCR region where to the ligands bind. Currently it has at least 19 known divisions/subfamilies.
• Family B (secretin receptor family) has about 60 members (currently divided in 3 subfamilies), presents a large N-terminal ectodomain and, although lacking the family A structural signature, they have morphological similarities to group A3. Their ligands include low molecular weight hormones such as GH-RH, but mainly polypeptide hormones such as glucagon, calcitonin, and secretin.
• Family C (metabotropic glutamate receptor family) has about 24 members that also have large ectodomains for ligand binding. Besides the different mGluR types, the family also comprises GABA-B receptors and putative pheromone receptors coupled to the heterotrimeric G Protein G_o_.
• Family D (fungus pheromone receptor family) is not found in vertebrates, and contains pheromone receptors (VNs) associated with the heterotrimeric G protein G_i_.
• Family E (cAMP receptor family) is comprised by cAMP receptors (cAR) and is also not found in vertebrates.
• Family F (frizzled/smoothened receptor family) includes the “frizzled” and “smoothened” receptors involved in embryonic development and in cell polarity and division.
• Family O: GPCRs that did not belong to any of the aforementioned families were later on assigned to a new family termed O (for ‘Other’) (Tuteja, 2009).

An alternative classification system, termed “GRAFS,” was proposed by Fredriksson et al. (2003), and divides GPCRs into 5 classes/families according to their primary sequence homology: Glutamate, Rhodopsin, Adhesion, Frizzled/Taste2, and Secretin. The main differences between this nomenclature system and the A-F(O) one are the division of Family B into Secretin and Adhesion families, the inclusion of several unique classified receptor proteins, and the Taste 2 receptors. The Secretin family has 15 members that have an extracellular hormone-binding domain, bind peptide hormones and have conserved cysteine residues that form a network of three cysteine bridges in their N-termini (Grauschopf et al., 2000). The Adhesion family is the second largest GPCR family in humans, with 33 members, and has long and diverse N-termini (Bjarnadóttir et al., 2004) and a GPCR proteolytic (GPS) domain that Secretin receptors do not have (Fredriksson et al., 2003; Lagerström and Schiöth, 2008).

Nowadays, pharmacologists generally use the GRAFS sequence similarity family-based classification, supplemented by a ligand-based functional classification. GPCRs usually bind to more than one ligand, but are commonly known according to the main endogenous one, such as dopamine or serotonin, in a third ligand-based classification. This last will be the classification used throughout this review, which will be focused on GPCRs coupled to heterotrimeric G proteins of the G_i/o_ subfamily (Figure 1B).

## Heterotrimeric G Proteins of the G_i/o_ Subfamily

G protein-coupled receptors are so called since they translate the signal from extracellular ligands into intracellular responses via transducers named ‘heterotrimeric G proteins’ (Resende and Vieira, 2012). Heterotrimeric G proteins are termed as such due to their three subunits: α, β, and γ, with the first being a GTP-binding protein with GTPase activity, and the latter two forming a single inseparable complex usually called the G_βγ_ subunit. Heterotrimeric G proteins are divided into four families according to the functional and structural homologies of their G_α_ subunits, which are responsible for the G_αβγ_ tri-complex main properties: G_i/o_, G_s_, G_q/11_, and G_12/13_ (Gudermann et al., 1996; Offermanns, 2003; Hollmann et al., 2005; Yudin and Rohacs, 2018). The G_s_ proteins act as stimulators of adenylyl cyclases (AC), leading to increased levels of cAMP and activation of downstream pathways, including PKA; the G_q/11_ proteins act as stimulators of phospholipase C, which in turns produces the intracellular messengers DAG and IP_3_, resulting in the activation of PKC and calcium signaling; the G_12/13_ proteins act as activators of the RhoA small GTP binding protein and of phospholipase D to regulate cell shape and motility; in contrast, the G_i/o_ proteins are generally described as inhibitory, with AC and potassium channels as their main effectors (Nobles et al., 2005; Milligan and Kostenis, 2006; Yudin and Rohacs, 2018).

The G_i/o_ family is composed of 8 genes. The three G_i_ proteins, G_αi1_, G_αi2_, and G_αi3_, inhibit some adenylyl cyclase isoforms (with ‘i’ referring to its inhibitory effect), reducing the ability of basal and G_s_-stimulated AC to generate cAMP. Other members of this family are G_t1_, G_t2_ (transducin) and G_gust_ (gustducin), together with G_z_ and G_o_ proteins. G_t1/2_ and G_gust_ are involved in visual and taste functions, respectively, and activate cGMP-phosphodiesterase. G_z_ inhibits AC, stimulates K^+^ channels and interacts with several protein RGSs. G_z_ is also phosphorylated by protein kinase C (PKC) and p21-activated kinase 1 (PAK1). Finally, the G_o_ protein (‘o’ standing for ‘other’) was discovered during G_i_ purification from bovine brain (Sternweis and Robishaw, 1984). G_o_ effect on AC is not clear: while initial reports indicated that G_o_ did not inhibit AC (Katada et al., 1986; Jiang and Bajpayee, 2009), more recent studies point to its ability to inhibit the AC 1 isoform, although not the AC V or VI ones (Birnbaumer, 2007). The G_o_ gene (*GNAO*) transcript is alternatively spliced into G_α1_ and G_α2_ variants, and some studies suggest that G_o_ ability to modulate AC activity is primarily due to its less studied G_o2_ isoform or via its βγ subunit (Kobayashi et al., 1990; Jiang and Bajpayee, 2009). G_o_ also seems to have a significant role in modulating several other signaling pathways, including the STAT3 and ERK pathways (van Biesen et al., 1996; He et al., 2005; Bikkavilli et al., 2008; Jiang and Bajpayee, 2009; Zhao et al., 2016). All of these G_i/o_ family members, except for the G_z_ protein, are inactivated by the pertussis toxin (Offermanns, 2003; Hollmann et al., 2005) and all induce K^+^ channels opening, besides functioning via other transduction mechanisms. G_i/o_-coupled GPCRs stimulate GIRK (G-protein activated Inwardly Rectifying K^+^) channels through direct interactions between G_βγ_ and the channel, leading to hyperpolarization and thus decreased neuronal excitability (Whorton and MacKinnon, 2013; Yudin and Rohacs, 2018). Importantly, although G_o_ is the most abundant heterotrimeric G protein in the central nervous system (CNS), constituting about 1% of the total membrane protein in the brain (Jiang and Bajpayee, 2009), very little is known about its functions, with this being particularly true for the G_α2_ isoform (Jiang et al., 2001; Jiang and Bajpayee, 2009; Wang et al., 2011; Tang et al., 2012).

**FIGURE 2:**
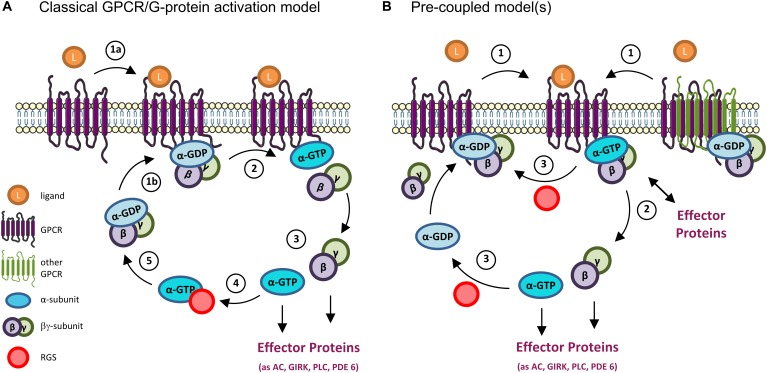
GPCR and G-proteins activation models. **(A)** Classical GPCR/G protein activation model. When inactive, heterotrimeric G proteins exist as a complex comprising the α subunit (bound to GDP) and the βγ subunit. (1a) The binding of a ligand (as a neurotransmitter) to a GPCR results in a conformational change that allows (1b) the binding of the receptor to the G protein. (2) This in turn causes a second conformational change on the α-subunit that results in the exchange of its GDP by GTP, and the separation of the α and βγ subunits. (3) At this point the G protein is active, and both the α and βγ subunits interact with downstream effectors to modulate different signaling pathways. (4) The G protein activation is terminated by the hydrolysis of the GTP molecule into GDP, a reaction that can be accelerated by the binding of Regulators of G-protein Signaling (RGS) to the α subunit. (5) The GTP hydrolysis results in the re-formation of the trimeric complex, bringing the G protein back to its inactive state. **(B)** Pre-coupled model(s). New mechanisms of GPCRs’ activation have emerged from various studies, particularly on G_i/o_-coupled GPCRs. In these, inactive GPCRs monomers (left) or homo/hetero dimers (right) exist in an inactive G protein-binding state. In some cases, effector proteins (as adenylyl cyclase, AC) can even be part of the inactive pre-coupled receptorsome complex (not shown). (1) When the GPCR is activated by a ligand (as dopamine, serotonin, etc.) (2) its coupled G protein becomes active by exchanging GDP by GTP and either dissociates from the GPCR and its βγ subunit to initiate the signaling response (left) or remain associated to the complex and activate downstream effector proteins at the membrane (right). (3) In either case, the GTPase activity (accelerated, e.g., by binding to RGS proteins) terminates the activation cycle for that G protein, and the GPCR-inactive G protein complex reassembles/is reinstalled. Note that, in both classical and pre-coupled models, when an active G_α_ subunit dissociates from the GPCR, another cytosolic inactive heterotrimeric G protein can bind to the activated GPCR to get activated, in an amplification mechanism. GIRK, G-protein activated Inwardly Rectifying K^+^ channel; PLC, phospholipase C; PDE 6, phosphodiesterase 6.

In the classical model of activation/deactivation of G proteins, the G_α_ subunit is in an inactive state when bound to GDP, forming a complex with the G_βγ_ subunit. When a ligand binds to a GPCR [the most common Guanine Nucleotide Exchange Factor (GEFs)], the receptor changes its conformation and binds to a heterotrimeric G protein, causing a conformational change in the α subunit of the G protein. This causes the exchange of GDP for GTP in the G_α_ subunit and the separation of the α subunit from the βγ ones (and from the GPCR). At this point, the G protein is active and both subunits (G_α_ and G_βγ_) can interact with and modulate the activity of several effector proteins. The G protein signaling is terminated due to the intrinsic GTPase activity of G_α_, which hydrolyzes its GTP to GDP, with consequent re-association of G_α_ to G_βγ_ and inactivation of the G protein (Offermanns, 2003; Hollmann et al., 2005; Baltoumas et al., 2013). Negative regulators, like Regulators of G protein signaling (RGS), can bind to an activated G_α_ subunit and accelerate its GTPase activity, leading to a faster inactivation and reassembling of the heterotrimeric G protein (De Vries et al., 2000; Hollinger and Hepler, 2002; Lin et al., 2014) (Figure 2A).

Alternatively, a pre-coupled model (Figure 2B) has gained ground, particularly for G_i/o_-coupled GPCRs, the focus of this review. In this, inactive GPCRs in the rest state are in a heterotrimeric G protein-binding state from which they dissociate when activated. When this occurs, the G protein is also activated, exchanges GDP by GTP in the G_α_ subunit, and initiates the signaling response. This also allows for the active GPCR to bind to another G protein, in a catalytic activation and signal amplification cycle (Figure 2B). This GPCRs-G protein pre-coupled model explains why GPCRs have high affinity binding to agonist ligands in the absence of GTP, and why many G_i_-coupled GPCRs can be co-purified with bound inactive G_i_ protein (Carpenter et al., 2016; Gurevich and Gurevich, 2017; Nehmé et al., 2017).

Indeed, several evidence have emerged supporting other activation models than the classical one, including observations that (1) GPCRs can form either hetero- or homodimers and create functional pre-coupled complexes with heterotrimeric G proteins in the rest state (Han et al., 2009; Parker et al., 2011; Ferre et al., 2014; Nishimura et al., 2017; Prieto, 2017; Durdagi et al., 2018; Navarro et al., 2018; Tóth et al., 2018; Calebiro and Koszegi, 2019); (2) the G proteins can exist in a pre-coupled complex with the GPCR and an effector protein, as AC; further, binding of agonists to GPCR might result in the activation of the G protein without its dissociation from the complex (Ferré, 2015; Navarro et al., 2018); (3) some G_α_ subunits can interact and modulate downstream signaling even when bound to GDP (Kamakura et al., 2013; Lin et al., 2014); (4) newly identified modulators of G protein activity, called GEMs (guanine exchange modulators), can activate and inhibit different G_α_ subunits through the same motif (Gupta et al., 2016; Ghosh et al., 2017).

## Brain-Enriched GPCRs Coupled to G_i/o_

Many of the physiological roles of GPCRs are determined by the heterotrimeric G proteins to which they couple (Park et al., 2012; Masuho et al., 2015). The coupling between GPCRs and the G_i/o_ family is of particular interest in the brain since G_i/o_ proteins are generally inhibitory, decreasing neuronal excitability, and can be the target not only for, e.g., analgesics, antipruritics, but also for several modulatory medications (Yudin and Rohacs, 2018). Further, the oligomerization properties that are emerging for some of these GPCRs, together with the high abundance of G_o_ in the CNS (Jiang and Bajpayee, 2009) and its less understood functions and signaling mechanisms, all these made G_i/o_-coupled GPCRs the central topic of this review.

From all GPCRs, 21.9% couple exclusively to the G_i/o_ subfamily, and other 5% can couple to proteins of the G_i/o_ and of other G subfamilies. Almost all of these GPCRs belong to the Glutamate and Rhodopsin families (Offermanns and Rosenthal, 2008), but some receptors of the F/Frizzled/smoothened family also have been reported to couple to G_i/o_. These include G_i_-coupled smoothened receptors signaling for Sonic Hedghog (Polizio et al., 2011a,b; Carbe et al., 2014; Cheng et al., 2018; Ho Wei et al., 2018) and Frizzled 6 Wnt receptor (Kilander et al., 2014a,b; Arthofer et al., 2016). Noteworthy, all these receptors may couple differentially among different G_i_ and G_o_ isoforms, and specifically prefer one specific isoform to the other(s) (Jiang et al., 2001, 2002; Jiang and Bajpayee, 2009; Wang et al., 2011; Tang et al., 2012), a topic that should be further explored by researchers.

Data regarding the relative protein abundance of G_i/o_-coupled GPCRs in human tissues was obtained from ‘The Human Protein Atlas’ database (v16.1.proteinatlas.org) (Uhlén et al., 2015), and is presented in the Supplementary Table S1. A short summary of this table’s information is here shown as a relative abundances chart (Figure 1B). This chart reveals that GPCRs coupled to G_i/o_ have a wide distribution pattern throughout various human body tissues/regions, being present in virtually all human tissues in relevant amounts. Nevertheless, Figure 1B also indicates that G_i/o_-coupled GPCRs are highly abundant in the brain, hematopoietic and immune system, lung, gastrointestinal tract, endocrine tissue and reproduction systems, while being slightly less abundant in pancreas, adipose and other soft tissues. Based on this graph we have further focused this review on G_i/o_-coupled GPCRs highly abundant in the brain (with a ‘high’ relative abundance in Supplementary Table S1) and/or with high brain specificity (present in no more than 4 tissues other than brain). These criteria retrieved the following 12 GPCRs: adrenoceptors, dopamine receptors, acetylcholine receptors, serotonin receptors, purinoceptors, opioid receptors, somatostatin receptors, angiotensin receptors, cannabinoid receptors, leukotriene receptors, metabotropic GABA receptors, and metabotropic glutamate receptors. Lysophospholipid receptors are also highly abundant in brain but also in various other body tissues, and were here excluded.

G_i/o_-coupled GPCRs have several functions within the nervous system, and a better understanding of the mechanisms regulated by these receptors is essential for the search of new therapeutic targets (Liu et al., 2018). The functions of G_i/o_-coupled GPCRs can be detected and studied at a molecular and cellular level, such as neurite outgrowth, neurotransmitter release and synaptic plasticity, which are then translated into complex brain functions, such as memory, learning, and cognition. Serotonin, dopamine, cannabinoid, and metabotropic glutamate receptors are involved in mechanisms of short and long-term memory, memory consolidation, and learning (Abush and Akirav, 2009; Leung and Wong, 2017). Opioid and serotonin receptors are involved in the perception and processing of pain (Dong et al., 2001; Diniz et al., 2015). Most GPCRs, including dopamine, serotonin, and opioid receptors also play a prominently role on modulating and influencing behavior and emotions (Baik, 2013; Chu Sin Chung and Kieffer, 2013; Nautiyal et al., 2015). Sleep and the regulation of the circadian rhythm are also highly regulated by GPCRs signaling, although it is not clear if the main GPCRs involved act through the G_i/o_ family (Civelli, 2005, 2012; Tsuneki et al., 2016). Additionally, GPCRs play a crucial role on brain development, by modulating different mechanisms, such as neuronal migration, neurite outgrowth and axonal elongation. Some of the GPCRs responsible for these actions include somatostatin, dopamine, and cannabinoid receptors (Leroux et al., 1995; Le Verche et al., 2009; Ma’ayan et al., 2009; Zhang et al., 2009). A detailed list of G_i/o_-coupled GPCRs functions on the nervous system can be found in Table 1.

**Table 1:** Brain-enriched and/or neurological-relevant GPCRs that can couple to the Gi/o subfamily, and their functions.

Receptor classes		Gα protein	GPCRs’ ligand(s) and general functions; subtypes and their specific functions
Adrenoceptors	α2A (ADRA2A)	Gi and Go	**Ligand:** Adrenaline > noradrenaline
			• Their activation causes platelet aggregation, blood vessel constriction and constriction of vascular smooth muscle;
	α2B (ADRA2B)		• Presynaptically, these receptors inhibit the release of noradrenaline (negative feedback control);
			• **α2B and α2C:** positive regulation of neuronal differentiation.
	α2C (ADRA2C)		
Dopamine receptors	D2 (DRD2)	Gi/Go	**Ligand:** Dopamine
	D3 (DRD3)		• Their activation mediates inhibitory dopaminergic neurotransmission;
	D4 (DRD4)		**D2:** important in the reward effects of morphine; knockout mice exhibit abnormal synaptic plasticity and display reduced levels of aggression; adenohypophysis development; associative learning; axonogenesis; branching morphogenesis of nerves; cerebral cortex GABAergic interneuron migration; chemical synaptic transmission, postsynaptic; long-term memory; negative regulation of synaptic transmission, glutamatergic; neurological system process involved in regulation of systemic arterial blood pressure; neuron-neuron synaptic transmission; orbitofrontal cortex development; positive regulation of dopamine uptake involved in synaptic transmission; positive regulation of glial cell-derived neurotrophic factor secretion; positive regulation of long-term synaptic potentiation; positive regulation of neuroblast proliferation; prepulse inhibition; regulation of dopamine uptake involved in synaptic transmission; regulation of long-term neuronal synaptic plasticity; regulation of synapse structural plasticity; regulation of synaptic transmission, GABAergic; response to axon injury; striatum development; synapse assembly; synaptic transmission, dopaminergic; visual learning.
			**D3:** their distribution is consistent with a role in cognition and emotional functions; implicated in modulation of cocaine self-administration; promotes cell proliferation**;** learning or memory; negative regulation of oligodendrocyte differentiation; prepulse inhibition; regulation of dopamine uptake involved in synaptic transmission; synaptic transmission, dopaminergic; visual learning.
			**D4:** may be involved in the modulation of gastric acid secretion; responsible for neuronal signaling in the mesolimbic system of the brain, an area of the brain that regulates emotion and complex behavior; modulates the circadian rhythm of contrast sensitivity and inhibits adrenergic receptor-induced melatonin synthesis by α(1B)-D_4_ and β_1_-D_4_ receptors heterodimerization; knockout mice are supersensitive to several psychoactive substances, including ethanol, cocaine, and methamphetamine; inhibitory postsynaptic potential; positive regulation of dopamine uptake involved in synaptic transmission.
Acetylcholine receptors	M2 (CHRM2)	Gi and Go	**Ligand:** Acetylcholine
	M4 (CHRM4)		• Their activation mediates many of the effects of acetylcholine in the central and peripheral nervous system;
			• Seem to function as autoreceptors;
			• Knockout mice reveal an important neuromodulatory role for these receptors;
			**M2:** control of myocyte contraction; smaller role in the smooth muscle contractile response; may be involved in the regulation of body temperature; nervous system development; synaptic transmission, cholinergic;
			**M4:** inhibitory autoreceptor for acetylcholine; activation of the receptor in the striatum inhibits dopamine-induced locomotor stimulation in mice; synaptic transmission, cholinergic.
Serotonin receptors	5-HT1A (HTR1A)	Gi/Go	**Ligand:** Serotonin
	5-HT1B (HTR1B)		• **5-HT1A:** autoreceptors that inhibit cell firing; involved in many neuromodulative processes; implicated in the neuroendocrine regulation of adrenocorticotropic hormone (ACTH); role in the regulation of 5-hydroxytryptamine release, metabolism and levels in the brain – thereby affects neural activity, mood and behavior; plays a role in the response to anxiogenic stimuli;
	5-HT1D (HTR1D)		• **5-HT1B:** induce presynaptic inhibition and influence behavior; vascular effects, such as pulmonary and cerebral arteries vasoconstriction; may also have a role in the regulation of bone; regulates the release of 5-hydroxytryptamine, dopamine and acetylcholine in the brain, and thereby affects neural activity, nociceptive processing, pain perception, mood and behavior; chemical synaptic transmission; negative regulation of synaptic transmission, GABAergic; negative regulation of synaptic transmission, glutamatergic;
	5-HT1E (HTR1E)		• **5-HT1D:** affect locomotion and anxiety; involved in vascular vasoconstriction in the brain; implicated in feeding behavior, anxiety, depression; may have a stimulatory effect on growth hormone secretion; regulates the release of 5-hydroxytryptamine in the brain, and thereby affects neural activity; chemical synaptic transmission;
	5-HT1F (HTR1F)		• **5-HT1E:** exact function is presently unknown, due to the lack of selective ligands; may have an important evolutionary role in humans and may be involved in the regulation of memory; chemical synaptic transmission;
	5-HT5A (HTR5A)		• **5-HT1F:** chemical synaptic transmission;
			• **5-HT5A:** may play a role in sleep and serve as a presynaptic serotonin autoreceptor; chemical synaptic transmission.
Purinoceptors	P2Y12 (P2RY12)	Gi/Go	**Ligand P2Y12 e P2Y13:** ADP
			**Ligand P2Y14:** UDP-glucose
	P2Y13 (P2RY13)		**P2Y12:** role in amplification of platelet activation and aggregation and blood coagulation; glial cell migration; regulation of microglial cell migration;
	P2Y14 (P2RY14)		**P2Y13:** may play a role in hematopoiesis and the immune system.
Opioid receptors	δ (DOR-1)	Gi and Go	**Ligand delta (δ):** β-Endorphin, met/leu-enkephalin
	κ (KOR-1)	Gi/Go	**Ligand kappa (κ):** Dynorphin A
	μ (MOR-1)		**Ligand mu (μ):** β-Endorphin > Dynorphin A > met/leu-enkephalin
	OPRL1		**Ligand OPRL1:** Nociceptin/orphanin FQ
			• **δ:** role in the perception of pain and in opiate-mediated analgesia; developing analgesic tolerance to morphine; chemical synaptic transmission; neuropeptide signaling pathway;
			• **κ:** are believed to mediate analgesia and sedation (perception of pain), miosis and dieresis; role in mediating reduced physical activity and regulation of salivation upon treatment with synthetic opioids; may play a role in arousal and regulation of autonomic and neuroendocrine functions; chemical synaptic transmission; neuropeptide signaling pathway;
			• **μ:** are believed to mediate analgesia, hypothermia, respiratory depression, miosis, bradycardia, nausea, euphoria, and physical dependence; involved in neurogenesis; chemical synaptic transmission; neuropeptide signaling pathway; positive regulation of neurogenesis;
			• **OPRL1:** potential role in modulating various brain functions, including instinctive behaviors and emotions; role in modulating nociception and the perception of pain; regulation of locomotor activity by the neuropeptide nociception; chemical synaptic transmission; neuropeptide signaling pathway.
Somatostatin receptors	SSTR1	Gi and Go	**Ligand SSTR1-4:** somatostatin-14 = somatostatin-28.
	SSTR2		**Ligand SSTR5:** somatostatin-28 > somatostatin-14.
	SSTR3		• **SSTR1:** cerebellum development; forebrain development;
	SSTR4		• **SSTR2:** growth hormone secretion; glucagon secretion; immune responses; mediates the inhibitory effect of somatostatin-14; inhibits cell growth; stimulates neuronal migration; stimulates axon outgrowth; neuronal development and maturation; mediates negative regulation of insulin receptor signaling through PTPN6; inactivates SSTR3 receptor function; cerebellum development; forebrain development;
	SSTR5		• **SSTR3:** inhibition of angiogenesis; cerebellum development; forebrain development; chemical synaptic transmission; neuropeptide signaling pathway;
			• **SSTR4:** arachidonate release; activation of mitogen-activated protein kinase cascade; anti-proliferative action in tumor cells; cerebellum development; forebrain development;
			• **SSTR5:** GH secretion; insulin secretion; increases cell growth; chemical synaptic transmission.
Angiotensin receptors	AT1 (AGTR1)	Gi/Go, Gq/11, G12/13	**Ligand:** Angiotensin II > Angiotensin III
			• Their activation mediates the cardiovascular effects of angiotensin II and angiotensin III (vasoconstriction, heart rate, aldosterone secretion, blood pressure, and volume);
			• Regulation of cell proliferation.
Cannabinoid receptors	CB_1_ (CNR1)	Gi and Go	**Ligand:** Anandamide and 2-arachidonoyl glyceride
	CB_2_ (CNR2)		• **CB_1_:** inhibition of ongoing release of excitatory and inhibitory neurotransmitters; axonal fasciculation; memory; negative regulation of action potential; positive regulation of neuron projection development; regulation of synaptic transmission, GABAergic and glutamatergic; *trans*-synaptic signaling by endocannabinoid, modulating synaptic transmission; negative regulation of dopamine secretion;
			• **CB_2_:** effect on the immunological activity of leukocytes; immune suppression; induction of apoptosis; induction of cell migration; negative regulation of action potential; negative regulation of synaptic transmission, GABAergic.
Leukotriene receptors	LTB4R	Gi/Go and Gq/11	**Ligand:** Leukotriene B4
	LTB4R2		• **LTB4R:** may be the cardiac P2Y receptor involved in the regulation of cardiac muscle contraction; act as a co-receptor; for macrophage-tropic Human immunodeficiency virus 1 (HIV-1) strains; causes chemotaxis; neuropeptide signaling pathway;
			• **LTB4R2:** mediates chemotaxis of granulocytes and macrophages; inhibit chemotaxis; neuropeptide signaling pathway.
Metabotropic GABA receptors	GABA_B2_ (GABBR2)	Gi and Go	**Ligand:** γ-Aminobutyric acid (GABA)
			• Involved in the fine tuning of inhibitory synaptic transmission; pre-synaptic GABA receptor is an inhibitory neurotransmitter in the brain; postsynaptic GABA receptor decreases neuronal excitability; involved hippocampal long-term potentiation, slow wave sleep, muscle relaxation and anti-nociception; gamma-aminobutyric acid signaling pathway.
Metabotropic glutamate receptors	mGluR2 (GRM2)	Gi/Go	**Ligand:** Glutamate
	mGluR3 (GRM3)		• **mGluR2:** may be involved in suppression of neurotransmission; may be involved in synaptogenesis or synaptic stabilization; chemical synaptic transmission; glutamate secretion; regulation of synaptic transmission, glutamatergic;
	mGluR4 (GRM4)		• **mGluR3:** chemical synaptic transmission; regulation of synaptic transmission, glutamatergic;
			• **mGluR4:** may have a role in modulation of glutamate transmission in the CNS; chemical synaptic transmission; neurotransmitter secretion; regulation of neuronal apoptotic process; regulation of synaptic transmission, glutamatergic;
	mGluR6 (GRM6)		• **mGluR6:** may have an important function in some inherited eye diseases; required for normal vision; chemical synaptic transmission; regulation of synaptic transmission, glutamatergic;
	mGluR7 (GRM7)		• **mGluR7:** may have a role in modulation of glutamate transmission in the CNS; chemical synaptic transmission; negative regulation of glutamate secretion; regulation of synaptic transmission, glutamatergic; sensory perception of smell; sensory perception of sound; short-term memory; transmission of nerve impulse;
	mGluR8 (GRM8)		• **mGluR8:** presynaptic receptor may modulate glutamate release at the axon terminals of mitral/tufted cells in the entorhinal cortex; regulation of synaptic transmission, glutamatergic.
Oxytocin receptors^∗^	OT (OXTR)	Gi/Go but mainly Gq/11	**Ligand:** Oxytocin
			• Stimulates contraction of uterine smooth muscle and of mammary gland; stimulates milk secretion in response to suckling; memory; positive regulation of synapse assembly, positive regulation of synaptic transmission, GABAergic and glutamatergic; positive regulation of norepinephrine secretion; sleep; social behavior.
Adenosine receptors^∗^	A1 (ADORA1)	Gi/Go	**Ligand:** Adenosine
			• **A1:** distributed widely in peripheral tissues where they have a mainly inhibitory role; cognition; excitatory postsynaptic potential; negative regulation of long term synaptic depression; negative regulation of mucus secretion; negative regulation of neurotrophin production; negative regulation of synaptic transmission, GABAergic; negative regulation of synaptic transmission, glutamatergic; nervous system development; regulation of respiratory gaseous exchange by neurological system process;
			• **A3:** may play a role in reproduction.


*^∗^The protein levels of these receptors in the brain were not reported in the Human Protein Atlas. However, as these GPCRs are known to play important functions in the brain, and their protein levels decrease with age, they were also included in this analysis. Data was taken from UniProt (The UniProt Consortium, 2017) and InterPro (Petryszak et al., 2016) database and included brain related Gene Ontology (GO) annotations (Molecular functions and Biological processes). ‘Gi and Go’, GPCRs that couple with both Gi and Go proteins; ‘Gi/Go’, GPCRs known to couple with at least one of these Gα proteins (Gimpl and Fahrenholz, 2001; Guo et al., 2001; Hunyady and Catt, 2006; Peres et al., 2007; Svízenská et al., 2008; Jiang and Bajpayee, 2009; McQuail et al., 2013).*

## Age-Induced Alterations in Brain-Enriched G_i/o_- Coupled GPCRs

Age-related alterations in neurotransmitters, their receptors, and related neuromodulatory systems may affect the responsiveness of receptors to neurotransmitters, leading to abnormal signal transduction activity via the receptors themselves or via other GPCRs they associate with at the membrane. As such, alterations in GPCRs’ expression and activity with aging can be associated to aging hallmarks and age-related pathologies. Decreased expression or activity of GPCRs and G proteins have indeed been associated to alterations in neuronal plasticity and increased sensitivity to neurodegenerative processes, with a consequent decline in cognitive, motor, and even sensory capabilities (Mesco et al., 1991; González-Maeso et al., 2002; Mato and Pazos, 2004). These associations suggest a crucial role for GPCRs in aging and in age-related and protein aggregation-associated pathologies, such as Alzheimer’s, Parkinson’s, and Huntington’s diseases, as well as vascular and frontotemporal dementia (Mesco et al., 1991; González-Maeso et al., 2002; Alemany et al., 2007; Huang et al., 2017).

**FIGURE 3:**
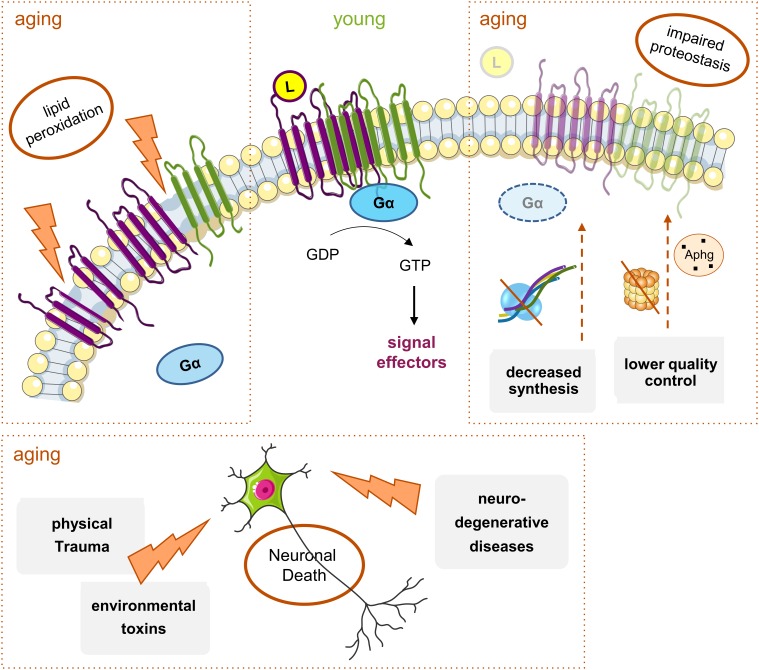
Main mechanisms of age-induced GPCRs decline. With aging, there is a general decrease in G_i/o_-coupled GPCRs protein levels throughout the brain. Though many elements are involved, there are three main factors: (1) alterations in the plasma membrane structure and fluidity caused by events like lipid peroxidation; these lead to the instability of membrane proteins, including GPCRS, and result either in their altered function or decreased levels and densities at, e.g., signaling nanodomains; (2) hindered proteostasis: decreased protein synthesis due to alterations in transcription factors or increased mRNA instability, and accumulation of aberrant GPCRs and other signaling proteins due to less efficient proteasome or autophagy (Aphg) quality control systems, may lead to a decrease in the levels of functional GPCRs in the aged brain; (3) the increased incidence of neurodegenerative diseases and physical trauma with aging, together with exposure to toxins, all lead to neuronal death. Moreover, the alterations in protein synthesis and membrane structure can eventually contribute to the onset or progression of neurodegenerative pathologies, with a subsequent further decrease in some GPCRs’ levels.

Alterations in the signaling pathways evoked by GPCRs can occur with age at various molecular levels, from ligand- or G protein-GPCR interactions to downstream intracellular signaling pathways involving the G_i/o_ effector proteins. Altered structure or functionality of the plasma membranes’ lipids, and on the membrane fluidity by lipid peroxidation, can all induce changes in the GPCR’s activity and density (number of molecules in a given region as a membranar nanodomain). In addition to the lipid environment, the loss of receptors can result from reduced protein synthesis and increased accumulation of aberrant non-functional proteins due to impaired proteostasis (Gouveia et al., 2017), and from cell degeneration and death due to trauma, toxins or pathologies (Huguet et al., 1994; Cunha et al., 1995; Alemany et al., 2007) (Figure 3).

A compilation of age-related alterations in the brain densities of relevant G_i/o_-coupled GPCRs and in their affinities to ligands or other compounds, can be found in Supplementary Table S2. Notwithstanding some contradictions, general lines could be taken from the compiled studies and are discussed below. A tendency can be observed for a general age-related decrease in the brain density of these GPCR receptors. In fact, the density of 64.3% of the studied G_i/o_-coupled receptors subtypes decreased with aging (corresponding to 44.1% of the density studies). GPCRs densities were observed to increase in only ∼10.5% of the studies, although corresponding to the other third of the analyzed GPCR subtypes (35.7%). Around 45.4% of the studies retrieved unaltered GPCR densities with advanced age. The affinity of the receptors to their ligands seems to not have suffered as much with age as their density. Indeed, 78% of the analyses to brain G_i/o_-coupled GPCRs affinities did not show alterations, ∼8.5% showed decreased affinity and 13.5% increased affinity to ligands. When both parameters (density and affinity) are combined in the ‘Binding potential’ of the GPCRs to their ligands, an age-related decrease of 50.6% is observed. Of note, the ‘Binding potential,’ which is calculated as the ratio between *B*_max_ (total density of the receptor) to *K*_D_ (the equilibrium dissociation constant of the tested radioligand), is thus a parameter that decreases when the receptor’s density decreases or when it has a higher tendency to dissociate from its ligand, as for example a decrease binding to an agonist due to a lower level of pre-coupled association with a G protein in the resting state.

Age-associated changes vary not only between GPCRs classes and subtypes, but also depending on the specific brain region (Pascual et al., 1991; Bigham and Lidow, 1995; Van Laere et al., 2008). These region-specific effects may be ascribed to the different rates of neuronal differentiation and maturation of the various brain regions (Sowell et al., 2003), leading to different patterns and rates of receptors’ biosynthesis and maturation after birth, besides the differential sensitivity to external and internal hazard (Hamilton et al., 1984; Araki et al., 1997). Specific information on age-related alterations of each GPCR class and subtype is presented below.

### Adrenoceptors

Age-associated changes in the densities of various adrenoceptors vary with the subtype of receptor to the brain area and specific layer (Bigham and Lidow, 1995). With age, the density of adrenoceptors particularly decreases in the striatum and various cortical regions (frontal, temporal, primary visual, prefrontal, motor, and somatosensory cortices) (Nomura et al., 1986) (Supplementary Table S2). The levels of α2-adrenoceptor may decrease with age due to the known loss of presynaptic noradrenergic terminals in layer I (Pascual et al., 1991; Bigham and Lidow, 1995). This reduction can have important functional consequences in synaptic integration of inputs originated from different cerebral regions (Chu et al., 2003), contributing to the motor and somatosensory deficits observed in elderly individuals. Alterations in the brain adrenergic system with advanced age seem to correlate with increased depression and memory loss incidence (Arango et al., 1992; Moore et al., 2005).

Abnormal CNS adrenergic activity has also been suggested in patients with Alzheimer’s disease (AD), with a decrease in the α2-adrenoceptor density being observed in AD patients’ brains (Meana et al., 1992).

### Dopamine Receptors

The dopaminergic system modulates neurotransmission in areas known for their vulnerability to aging, as the substantia nigra and striatum (areas related to movement control and affected in Parkinson’s disease, PD) and the hippocampus, a region with a role in learning and memory (affected in AD) (Kaasinen et al., 2000; Amenta et al., 2001).

Age-related decline in the dopaminergic neurotransmission (including decreased protein and mRNA levels of the D2 receptors and their binding potential) has been observed in various species, such as rabbits, rats and humans, and is one of the more consistent manifestations of neural aging and age-related degeneration (Thal et al., 1980; O’Boyle and Waddington, 1984; Wong et al., 1984; Henry et al., 1986; Joyce et al., 1986; Lai et al., 1987; Morgan et al., 1987; Seeman et al., 1987; De Keyser et al., 1990; Mesco et al., 1991; Weiss et al., 1992; Antonini et al., 1993;Rinne et al., 1993; Valerio et al., 1994; Wang et al., 1995; Ricci et al., 1996; Kaasinen et al., 2000; Inoue et al., 2001; Villar-Cheda et al., 2014; Matuskey et al., 2016; Dang et al., 2017) (Supplementary Table S2). The decline of dopamine receptors with aging might be a direct effect of the loss of dopaminergic and their target neurons in the aging brain and/or the expression of autoreceptors on dopamine neurons (Naoi and Maruyama, 1999; Branch et al., 2016; Dang et al., 2017). Indeed, cells in the substantia nigra project to the striatum with a regionally corresponding arrangement, and the observed reduction in dopamine receptors may also result from cortical and thalamic atrophy, and loss of striatal size with age (Morris et al., 1999; Kaasinen et al., 2000; Raz et al., 2003). Further, the decline in dopamine receptors also results from their reduced biosynthesis and incorporation into the neuronal membranes, partially due to decreased transcription in surviving dopaminergic neurons or increased receptor degradation (Morgan et al., 1987; Rinne, 1987; Han et al., 1989; Mesco et al., 1991; Antonini et al., 1993; Valerio et al., 1994). As such, age-related decrease of D2 receptor in striatum and neuronal cell loss in the substantia nigra have been associated to a decline in the synaptic dopamine reuptake due to age-related decline of the dopamine transporter (DAT) in the striatum (Ishibashi et al., 2009). Regarding gender, Pohjalainen et al. (1998) reported gender-related differences in D2 receptor affinity in the left striatum with age, which may contribute to the differential vulnerability of men and women to psychiatric disorders like schizophrenia (Pohjalainen et al., 1998). In contrast to D2, the D3 receptor levels were shown to increase with age in the striatum and nucleus accumbens, which might represent increased D3 receptor function in these regions to compensate D2 receptor striatal loss (Wallace and Booze, 1996). It is still controversial if D3 receptor levels change in the aged human substantia nigra, since [^11^C]-(+)-PHNO binding to D3 receptor was found either unaltered or increased (Nakajima et al., 2015; Matuskey et al., 2016). Alterations in the D4 receptor with age have been by far less studied (Valerio et al., 1994); however, it would be interesting to understand if age-associated circadian rhythm alterations are associated with altered D4 levels or their heterodimerization with adrenergic receptors (α_1_B and β_1_), with consequent mis-regulation of adrenergic receptor-induced melatonin synthesis (González et al., 2012). As dopamine neurons are associated with motor, cognitive and endocrine functions, the loss of dopamine neurons and receptors with age most likely contributes to age-related decreased frontal metabolism, cognitive and memory performances, and decreased motor functions (Mesco et al., 1991; Valerio et al., 1994). Indeed, the dopaminergic network seems to be intimately associated with the senescence process. Most D2 dopamine receptors in the CNS are coupled to the G_o_ protein, with the usually robust ability of GTP to regulate the binding affinity of dopamine to D2 receptors being absent in G_o_-deficient mice (Jiang et al., 2001).

In agreement to its tight relation with aging, alterations in dopamine receptors are also characteristic of age-related pathologies as PD, but also of other disorders, such as Gilles de La Tourette’s syndrome, depression, schizophrenia, and attention deficit disorder (Meng et al., 1999; Rangel-Barajas et al., 2015; Dang et al., 2017; Prieto, 2017).

### Acetylcholine Receptors

Acetylcholine (ACh) receptors play an essential role in neural network support, with their levels peaking very early (at 3 months of age) in the rat hippocampus (Tice et al., 1996). Different subtypes of ACh receptors appear to have different sensitivities to aging, with mRNA and protein levels of the muscarinic M2 receptor suffering a more pronounced age-induced decrease than the ones of the M4 receptors. Nevertheless, alterations in other subtypes of muscarinic receptors may not be as relevant as for M2. For example, significant changes in the density of the M1, M3, and M4 receptor subtypes were detected in the cerebellum but probably have a minimal impact on cerebellar function since over 90% of the cerebellum muscarinic receptors belong to the M2 subtype (Blake et al., 1991; Nordberg et al., 1992; Lee et al., 1994; Tice et al., 1996) (Supplementary Table S2).

The age-related decrease in the M2 densities at the hippocampus and frontal cortex may affect processes such as learning and memory and other cognitive functions (Tice et al., 1996; Scarr, 2012) (Table 1). Nieves-Martinez et al. (2012) reported that normal, healthy aged rats have an age-related increase in cognitive rigidity associated with reduced muscarinic receptor function in the dorsomedial striatum, even if there is no visible loss of M2/M4 receptor densities (Nieves-Martinez et al., 2012).

Age-related loss of ACh receptors or impairment of their function could also be implicated in the pathophysiology of age-associated CNS diseases, such as Parkinson’s and Alzheimer’s diseases (Tayebati et al., 2002, 2004; Piggott et al., 2003). However, activation of M2 and M4 mAChRs receptors aggravated the formation of the amyloid-β peptide and inhibited the release of the neurotrophic sAPPα peptide, potentiating AD (Thathiah and De Strooper, 2011).

### Serotonin Receptors

Limbic and neuroendocrine control areas are under the influence of serotonergic innervation (Arranz et al., 1993) and the effect of aging on the serotonergic signaling has been thoroughly studied (Supplementary Table S2).

Cognitive deficits (Arranz et al., 1993), changes in sleep (Arranz et al., 1993; Meltzer et al., 2001), food intake (Arranz et al., 1993; Meltzer et al., 2001), mood (Arranz et al., 1993), circadian rhythms, neuroendocrine function, affective state, memory and hormone secretion (Meltzer et al., 1998; Lerer et al., 1999), and decreased libido (Arranz et al., 1993) are some of the disturbances that have been attributed to age-related changes in the serotonergic system (Matuskey et al., 2012). Age-dependent decreases in 5-HT1-receptor binding potential (mainly resulting from alterations in the 5-HT1A subtype, see Supplementary Table S2) occur in the hypothalamus, in various cortical regions including the frontal cortex, and in the hippocampus and brainstem (these both only significantly in men) (Meltzer et al., 2001; Tauscher et al., 2001; Parsey et al., 2002; Matuskey et al., 2012). In the frontal cortex, there is a decrease in the receptor’s affinity (Bigham and Lidow, 1995), as well as a decrease in G protein activation induced by a 5-HT1A agonist (González-Maeso et al., 2002), revealing this region as being highly affected by age. Contrarily, an increase in 5-HT receptor binding potential was observed at basal ganglia (putamen, pallidum) (Matuskey et al., 2012) and enhanced downstream G protein signaling at the hippocampus (Duncan and Hensler, 2002). Indeed, although the 5-HT1A receptor density decreased in the aging hippocampal CA1 region (Burnet et al., 1994), 5-HT1A receptor-stimulated [^35^S]GTPγS binding increased, representing a compensatory mechanism at this region; this is not observed, e.g., in the dentate gyrus, in spite of it also presenting decreased 5-HT1A density (Duncan and Hensler, 2002). Gender-specific age-related decreases in 5-HT1A binding potential (Supplementary Table S2) were hypothesized to result from differences in hormonal levels and distribution, as of circulating estrogen (Meltzer et al., 2001). Reduction in 5-HT1 receptor density with aging may result from decreased receptor synthesis (Palego et al., 1997), loss of neuronal cell bodies (Dillon et al., 1991) and/or alterations in the lipid environment of neuronal cell membranes, namely increase in viscosity and reduction in fluidity due to lipid peroxidation (Dillon et al., 1991; Huguet et al., 1994; Palego et al., 1997).

Pathological alterations of the 5-HT metabolism and/or loss of serotonergic neurotransmission can also lead to several CNS pathologies, including AD, schizophrenia, depression, and anxiety. Serotonergic systems represent a potential therapeutic target for the treatment of AD, since decreased serotonergic neurons and/or reduction of serotonergic projections can be observed in AD patients (Butzlaff and Ponimaskin, 2016). Further, 5-HT1A receptor levels were reported to be decreased in the hippocampus and frontal cortex of AD patients (Mizukami et al., 2011; Verdurand and Zimmer, 2017). At a molecular level, altered serotonin signaling seem to have a role in the production of toxic amyloid proteins and amyloid plaques, leading to the progression of the disease (Butzlaff and Ponimaskin, 2016). Relatively to PD, a decrease in the 5-HT1A binding potential has been detected in the midbrain raphe of PD patients, correlating with the severity of tremors (Doder et al., 2003). In parallel, the density of these receptors was observed to be increased in the temporal cortex of patients with PD and Lewy body dementia experiencing depression (Sharp et al., 2008; Guerram et al., 2016).

### Opioid Receptors

The opioid system is involved in the modulation of several physiological processes, such as analgesia, stress response, immune response, synaptic activation and neuroendocrine function (Zhao et al., 2016). For example, kappa (κ) receptors are located on presynaptic axonal terminals, modulating the release of other classes of neurotransmitters, such as acetylcholine and substance P (Svingos et al., 2001). Further, opioids induce modifications in the endocrine system resulting in stimulatory or inhibitory effects on hormone release (Vuong et al., 2010). With advanced age, the capacity of opioids to regulate hormones of the pituitary gland is decreased, and it is suggested that receptor expression could depend on the endocrine environment (Carretero et al., 2004).

Age-related decreased densities of opioid receptors have been observed at the striatum, frontal poles, anterior cortex, hippocampus (Hess et al., 1981; Piva et al., 1987; Maggi et al., 1989) (Supplementary Table S2). The kappa receptors are the main affected ones, being particularly decreased at the substantia nigra, striatum (caudate and putamen) and various cortical regions including the lateral agranular field of frontal cortex. The potency of an antagonist of kappa receptors was also found decreased in the medial pre-frontal cortex (Sirohi and Walker, 2015). The decreased opioid binding to kappa receptors may result from age-related destruction of cell bodies and consequent degeneration of axons (Hiller et al., 1992). On the other hand, opioid signaling via the mu (μ) receptor seems to increase with age at the prefrontal cortex, but its density is decreased at the hypothalamus (González-Maeso et al., 2002).

The functions of the opioid system in learning and memory (Thathiah and De Strooper, 2011; Zhao et al., 2016) support a possible linkage between the opioid system and Alzheimer’s disease (Mathieu-Kia et al., 2001), which is also reinforced by the overlap between the distribution of opioid receptors and the localization of the amyloid plaques in AD patients (Zhao et al., 2016). Other links include the fact that opioids can modulate the release of acetylcholine and substance P, already associated with AD (Barg et al., 1993). Furthermore, the agonist-induced activation of the human delta (δ) opioid receptor increases the activities of β- and γ-secretase, which leads to the increased production of the amyloid-β peptide (Sarajärvi et al., 2015).

### Somatostatin Receptors

Somatostatin systems are highly expressed in the mammalian brain and are involved in many brain functions such as motor activity, sleep, and sensory and cognitive processes (Carretero et al., 2004). Somatostatin receptors (SSTRs) are also involved in some pathologies, such as Alzheimer’s disease, neuroendocrine dysfunctions and several types of cancer (mainly the SSTR2 subtype in the case of human tumors) (Patel, 1999).

Down-regulation of somatostatin receptors was observed in normal aging (Kumar, 2005; Gahete et al., 2010), and their affinity to ligands decreases with age at hippocampus, striatum and frontal cortex (Sirvio et al., 1987; Reed et al., 1999; Shimokawa et al., 2000) (Supplementary Table S2). Importantly, in contrast to rodents, human SSTRs maintain their density in the cerebellum (Gonzalez et al., 1992; Laquerriere et al., 1994), suggesting that somatostatin plays an important role in the mature human cerebellum (Laquerriere et al., 1994).

A decline of somatostatin receptors (possibly SSTR2 and SSTR4) was also observed in AD brains, and is related to the degree of dementia (Kumar, 2005; Gahete et al., 2010). Indeed, a decline in these receptors was observed to lead to reduced action of neprilysin, an enzyme that degrades the amyloid-β peptide, leading to the accumulation of amyloid-β in AD senile plaques. As such, somatostatin receptors are potential pharmacological targets for prevention and treatment of AD (Burgos-Ramos et al., 2008).

### Angiotensin Receptors

Angiotensin II, a ligand of angiotensin type 1 (AT1) receptors, is known to be an inducer of inflammation and oxidative stress (Villar-Cheda et al., 2012, 2014; Jackson et al., 2018).

Benigni et al. (2009) have observed that mice lacking AT1 receptors have higher longevity due to their lower oxidative stress, and have increased protection against age-related progression of atherosclerosis (Benigni et al., 2009).

In fact, the inhibition of the AT1 receptor has been suggested to aid in the attenuation of several cognitive pathologies (Jackson et al., 2018). Increased activity of the local renin-angiotensin system (RAS), via an increased expression of AT1 receptors in substantia nigra, seems to be involved in age-related loss of dopaminergic neurons and in an increased risk for PD due to the upregulation of NADPH-dependent oxidases (Villar-Cheda et al., 2012, 2014; Jackson et al., 2018). Further, inhibition of AT1 receptors in aged rats led to a decrease in their higher susceptibility to dopaminergic neurotoxins (Villar-Cheda et al., 2012, 2014).

### Cannabinoid Receptors

The cannabinoid system modulates multiple physiological processes, mainly in the central motor system, being involved in learning, memory, motor and reward processes, regulation of pain mechanisms and protection against neuroinflammation (Van Laere et al., 2008; Piyanova et al., 2013; Stumm et al., 2013).

Loss of cannabinoid 1 (CB1) receptors can result in neuronal loss and cognitive deficits characteristic of the aging brain (Piyanova et al., 2013; Stumm et al., 2013). With advanced age, CB receptors decrease and undergo biochemical and functional alterations in various cortical areas and in extrapyramidal structures (Supplementary Table S2). In the latter, selective degeneration of neurons with CB receptors, and a reduction in receptor synthesis involving a decline of mRNA levels, are possible mechanisms leading to the visible CB receptor loss (Mailleux and Vanderhaeghen, 1992; Romero et al., 1998; Mato and Pazos, 2004). The CB receptors also have their signaling through G proteins decreased at substantia nigra and frontal cortex (Romero et al., 1998; Mato and Pazos, 2004). These alterations are possibly implicated in the pathophysiology of distinct neurological and psychiatric disorders, such as PD, Huntington’s chorea, AD, schizophrenia and depression (Romero et al., 1998; Hurley et al., 2003; Mato and Pazos, 2004; Van Laere et al., 2008; Wong et al., 2010; Takkinen et al., 2018), with these receptors being considered a new target for drug treatment of neuropsychiatric disorders. At a molecular level, lack of CB1 receptors are suggested to influence the course of brain aging via induction of lipofuscin accumulation, reduced expression and activity of cathepsin D, and alterations in lysosomal protease activity with subsequent decreased degradation of damaged macromolecules in the hippocampus (Piyanova et al., 2013). Regarding the binding potential, gender- and region-dependent increases in the CB1 receptor binding were detected in the human memory, limbic and motor circuits (Van Laere et al., 2008). The authors hypothesize that these alterations may reflect a way to compensate age-related endocannabinoid dysfunction (decreased ligand levels at, e.g., the hippocampus), functional losses in other monoamine systems, or may even result from increased expression of the receptors in non-neuronal elements such as glial cells. Gender-dependent CB1R up-regulation can result, e.g., from compensatory mechanisms modulated by sex hormones (Van Laere et al., 2008). In mouse hippocampus (and parieto-temporal cortex), an increase in [18F]FMPEP-D2 binding is observed during aging, which could be a compensatory reaction against to the age-related endocannabinoid dysfunction that has been described in rodents (Maccarrone et al., 2001; Canas et al., 2009; Takkinen et al., 2018).

The mRNA levels of CB1R were found to be significantly decreased in the brains of patients with Parkinson’s disease, specifically in the striatum (caudate nucleus and putamen) and globus pallidus (Hurley et al., 2003). These alterations not only indicate a connection between changes in the dopaminergic system and the cannabinoid system, but also reinforce the cannabinoid receptors as potential targets in the treatment of PD.

### Metabotropic GABA Receptors

GABA is the main inhibitory neurotransmitter, being involved in a plethora of brain regulatory mechanisms (Petroff, 2002; Ko et al., 2015).

As our brain gets older, the density of GABA receptors particularly decreases in the frontal cortex (Supplementary Table S2). Age-induced decreased GABAergic signaling can have as consequence loss of memory and impaired cognitive abilities, related to both medial temporal lobe and frontal cortical systems (McQuail et al., 2012). These are brain regions particularly vulnerable to aging alterations, and aged rodents present frontal cortical dysfunction that causes loss of behavioral flexibility (Barense et al., 2002; Schoenbaum et al., 2002; Rodefer and Nguyen, 2008; McQuail et al., 2012). In humans, the protein density of GABA_B_R2 was found decreased in the frontal cortex (McQuail et al., 2012), while unaltered in the cerebellum, temporal gyrus, hippocampus, sensory and motor cortices (McQuail et al., 2012; Pandya et al., 2019). In addition, activation of the GABAergic signaling via downstream G proteins is also decreased with age at the prefrontal cortex (González-Maeso et al., 2002). Decreased GABAergic signaling may reflect a decrease in the GABA receptors-containing nerve terminals at specific brain regions, or be a consequence of peripheral loss of input (Milbrandt et al., 1994; Caspary et al., 2008). It may also relate to decreased function of its downstream G_i/o_ protein signaling (discussed later on). A recent study reported an age-related increase in the mRNA levels of several GABA receptors in the primary visual cortex of rhesus monkeys, suggested to be a compensatory feedback mechanism for the reduced release of GABA found in this region (Liao et al., 2016).

In age-related Alzheimer’s pathology, GABAergic neurotransmission undergoes a dynamic remodeling. Astrocytes in AD brains were observed to have up-regulated GABA release, which binds to extrasynaptic GABA receptors and inhibits synaptic function, causing memory and cognitive deficits (Jo et al., 2014). In this sense, GABA receptors may be promising therapeutic targets for AD, using antagonists to alleviate the inhibition of synaptic function and improve cognition (Li et al., 2016).

### Metabotropic Glutamate Receptors

Glutamate is considered the major excitatory neurotransmitter in the mammalian brain (Hovelsø et al., 2012; Brosnan and Brosnan, 2013), being implicated in diverse CNS signaling pathways, mediating neuronal excitability, synaptic plasticity and neurotransmitters’ release (Ferraguti and Shigemoto, 2006; Brosnan and Brosnan, 2013; Zhou and Danbolt, 2014).

With aging there is an excessive glutamate release (Freeman and Gibson, 1987), and increased glutamate-mediated excitotoxicity has been widely reported in age-related diseases (Sabelhaus et al., 2000). Regarding its receptors, there is an age-dependent increase in protein and mRNA levels of group II mGlu receptor proteins (mGluR2 and mGluR3). This occurs in various brain regions (Angulo et al., 2003), including the frontal cortex, striatum (caudate and putamen), thalamus, cerebellum, and the hippocampus (Supplementary Table S2). mGluR2/3 increased levels can result from increased synthesis of receptors to compensate the excessive release of glutamate with aging (Simonyi et al., 2005). Contrarily, mGluR7 mRNA levels decrease with age in various brain regions, and may contribute to the age-related decline in sensory functions (Simonyi et al., 2000).

Metabotropic glutamate receptors are extensively reported to be involved in Alzheimer’s disease. For example, down-regulation of group II mGluR signaling through the use of antagonists may be a therapeutic approach for AD, since these receptors stimulate the release of amyloid β42, the predominant isoform of the amyloid-β peptide accumulated in AD brains. However, group II mGluRs agonists have shown to be neuroprotective against amyloid β toxicity (Thathiah and De Strooper, 2011; Caraci et al., 2018). Further, in the striatum and thalamus, agonists of group III mGluR proteins (mGluR4, mGluR6, mGluR7, and mGluR8) have an inhibitory effect on glutamate release, and may counteract excitotoxic neurodegeneration associated with age-related pathologies (Sabelhaus et al., 2000; Simonyi et al., 2005). In this sense, activation of group III mGluRs has been established to be neuroprotective (Ribeiro et al., 2017; Caraci et al., 2018). Besides AD, these receptors are also implied in other neurodegenerative diseases such as Parkinson’s and Huntington’s diseases (Ribeiro et al., 2017).

The protein levels of the following two G_i/o_-coupled GPCRs are not available at the Human Protein Atlas database, but their mRNA levels are quite abundant in brain. Further, they are known to play important functions in the brain, and their protein levels change with age. Hence, they were subsequently included in this section.

### Adenosine Receptors

Adenosine receptors are involved in the modulation of several brain functions, such as cognition, neurodevelopment and the sleep-awake cycle (Burnstock et al., 2011; Chen et al., 2014; Huang et al., 2014).

With aging, a reduction in the turnover rate of adenosine receptors (Fredholm et al., 1998) and a decline in the functional output of the adenosine A1 receptor pathway (Meerlo et al., 2004) have been observed. Reduction in the A1 receptor binding may be related to a specific reduction in A1 receptor’s levels (Pagonopoulou and Angelatou, 1992; Sperlágh et al., 1997; Meyer et al., 2007), a specific neuronal loss, or alterations of the neuronal set-up of A1 receptors, since there is a replacement of neuronal cells by glial cells (Pagonopoulou and Angelatou, 1992; Cunha et al., 1995). It may also result from a modification of the lipid environment of the receptor due to membrane peroxidation, or by alterations in adenosine receptors’ mRNAs due to a decreased transcription of the receptor gene and/or a decrease in its mRNA stability (Cunha et al., 1995; Cheng et al., 2000). These changes can have implications in the plasticity and function of the brain, and be associated to ischemia/hypoxia, dementia, and AD (Ekonomou et al., 2000; Meerlo et al., 2004; Meyer et al., 2007).

Regarding the AD pathology, a significant increase in A1 receptors was observed in degenerating neurons presenting neurofibrillary tangles, and in dystrophic neurites of senile plaques in the hippocampus and frontal cortex of AD patients (Angulo et al., 2003; Albasanz et al., 2008).

### Oxytocin Receptors

Oxytocin (OT) receptors are present in the CNS, with oxytocinergic neurons presenting widespread projections and modulating synaptic transmission and network activity in the hippocampus (Arsenijevic et al., 1995; Mairesse et al., 2015).

Age-related reduction in OT binding was reported in the caudate putamen, the olfactory tubercle, and the ventromedial hypothalamic nucleus of 20-month old rats. These reductions were due to a decrease in the binding area rather than from a decrease of the binding density within each area (Arsenijevic et al., 1995). The authors also hypothesized a link between this reduction in binding and a reduction in steroid hormones, such as testosterone, detected in the aged animals. This could be especially true for the olfactory tubercle and the ventromedial hypothalamic nucleus, since previous studies showed a modulatory effect of steroid hormones on the expression of OT receptors in these areas (Tribollet et al., 1990). Supporting this idea, a study showed that there were no significant alterations in OT ligand binding in ‘steroid-independent’ regions, such as the anterior olfactory nucleus, the dorsal peduncular nucleus and the ventral subiculum. However, this correlation between steroid action and OT binding did not apply to all areas of the brain. In the bed nucleus of stria terminalis and central amygdaloid nucleus, there were no alterations in binding despite the sensitivity of these areas to gonadal steroids. The loss of striatal OT receptors might also be due to neuronal cell death, to a decrease in the striatal dopamine content and of dopamine D2 receptors, since a stimulatory effect on the secretion of OT has been suggested for dopamine in some reproduction-related situations (Arsenijevic et al., 1995).

An analysis of the receptors discussed in this section indicates that, during the aging process, there is mainly a reduction in G_i/o_-coupled GPCR densities in particular brain areas (further summarized in Section “Conclusion”). This reduction most probably results from peroxidation of the membrane, imbalanced proteostasis (reduced biosynthetic capacity and impaired protein quality control), and neuronal degeneration and death (Figure 3). Additional insight on how these systems changes with age can be gathered by taking into account the extent of the area affected with aging and the differentially availability of the GPCRs in the brain (Dang et al., 2017). Regarding receptors’ affinities, although most of the studies reported no alterations, around 8.6% of GPCRs showed decreased binding potential.

Besides alterations resulting from the normal aging of the brain, altered GPCR signaling strongly correlates with the development of age-associated and other neuropathologies (Huang et al., 2017). In Alzheimer’s disease brains there is a decline in the densities and/or function of, e.g., α2-adrenoceptor (Meana et al., 1992), acetylcholine (Tayebati et al., 2002, 2004), 5-HT1A serotonin (Mizukami et al., 2011; Butzlaff and Ponimaskin, 2016; Verdurand and Zimmer, 2017), and somatostatin receptors (Kumar, 2005; Gahete et al., 2010), but an increase in adenosine A1 receptors (Albasanz et al., 2008). Decreased serotonin, glutamate, somatostatin, adenosine, and opioid receptors/transmission have all been associated with changes in cognitive and/or memory functions and with AD and the degree of dementia, together with increased metabotropic glutamate (group II mGluR) and extrasynaptic GABA signaling (Mathieu-Kia et al., 2001; Kumar, 2005; Albasanz et al., 2008; Gahete et al., 2010; Thathiah and De Strooper, 2011; Jo et al., 2014; Sarajärvi et al., 2015; Butzlaff and Ponimaskin, 2016; Li et al., 2016; Hauser et al., 2017; Caraci et al., 2018). In Parkinson’s disease, the levels, binding potential and/or function of GPCR receptors for dopamine (Meng et al., 1999; Rangel-Barajas et al., 2015; Dang et al., 2017), acetylcholine (Tayebati et al., 2002, 2004; Piggott et al., 2003) and serotonin (Doder et al., 2003) were found decreased. Further, declines in the levels of dopamine (Rangel-Barajas et al., 2015; Guerram et al., 2016), serotonin (Doder et al., 2003; Sharp et al., 2008), acetylcholine (Piggott et al., 2003; Tayebati et al., 2004), mGluR group III glutamate (Ribeiro et al., 2017) and cannabinoid receptors (Hurley et al., 2003) have been implicated in PD progression and symptoms, as well as increased signaling via angiotensin and mGluR group II metabotropic glutamate receptors (Villar-Cheda et al., 2012, 2014; Ribeiro et al., 2017; Jackson et al., 2018). Recent detailed information on the relation between GPCRs and neuropathologies can be found in Guerram et al. (2016), Huang et al. (2017), and Lütjens and Rocher (2017).

Degradation of specific G_i/o_-coupled GPCR signaling with aging and age-associated diseases may not only derive from the decreased levels of that specific GPCR, but also of other molecular players influencing the signaling mechanism, including the main ligand, membranar lipids and other GPCR interactors at the plasma membrane. These may include other GPCRs with which the GPCR in question dimerizes/oligomerizes (see below), regulators (e.g., GIT2, β-arrestin, allosteric regulators) (Chen et al., 2001; Desai and Miller, 2018; Durdagi et al., 2018; van Gastel et al., 2018), molecules of the signal transduction machinery including the G_i/o_ transducers and the AC effector (Kristiansen, 2004), all composing a functional receptorsome. Recent studies show that many GPCRs (including dopamine D_1-3_R, serotonin 5HT, angiotensin AT_1_R, purinoreceptor P2YR, cannabinoid CB_1_, adrenergic β_2_AR, etc.) form functional homo- and heterodimers to successfully translate extracellular signals (Ferre et al., 2014; Nishimura et al., 2017; Prieto, 2017; Durdagi et al., 2018; Tóth et al., 2018). Alterations in the assembly of the GPCR receptorsome complexes can alter the affinity to certain ligands, lead to desensitization or disruption of the signaling mechanism, potentially explaining the decreased binding potential and altered signaling detected in age-related studies, including specific deregulations of GPCR signaling in age-associated diseases (Grumolato et al., 2010; Ferré, 2015; Prieto, 2017; Durdagi et al., 2018; Maroteaux et al., 2019).

These new mechanisms comprising GPCRs dimerization and association with other signaling molecules reveal that GPCRs are not single pharmacological entities and explain why alterations in the plasma membrane, associated to aging and its diseases, affect the efficacy of drugs targeting GPCRs (Desai and Miller, 2018). Further, due to this oligomerization and cooperative character, one may alter GPCR signaling by targeting other components of the receptorsome (Nishimura et al., 2017; Durdagi et al., 2018), including other GPCRs. For example, the targeting of the AT_1_R angiotensin receptor, a signaling hub of receptor crosstalk whose levels are increased in areas mainly affected by age, is expected to aid in the recovery of signaling from other GPCRs with which it dimerizes (Tóth et al., 2018). The new knowledge on GPCR dynamics is thus a driving force in the research and development of new therapeutic drugs for age-associated diseases, from neurodegenerative to cardiovascular diseases, osteoporosis, type 2 diabetes mellitus, etc. (Guerram et al., 2016; Huang et al., 2017; Lütjens and Rocher, 2017; Tóth et al., 2018; van Gastel et al., 2018; Calebiro and Koszegi, 2019; Maroteaux et al., 2019). To achieve this goal it is necessary to understand how aging and age-associated disease impact other receptorsome components such as the downstream heterotrimeric G proteins.

## G_i/o_ in Brain Development and Aging

With age, the signaling downstream several G_i/o_-coupled GPCRs is altered, and may also be ascribed to alterations in their heterotrimeric G protein transducer. The alterations that these proteins suffer during brain development and aging are presented here, with a particular focus on G_αo_, the most abundant G_α_ subunit in the brain.

Regarding postnatal development, the brain mRNA levels of G_αo_ and G_αi_ were found to be slightly higher at P1 than in adulthood (Duman et al., 1989). G_αo_ and G_αi_ mRNA levels increased during the first 7 days postnatally and then decreased until P25 to levels equal to the adult brain. G_αs_ and G_β_ (the other G_α_ subunits analyzed) followed a similar pattern (Duman et al., 1989). Of note, this study did not specify the age of adult rats, and the characterization of a rat as “adult” or in ‘early adulthood’ can be based in a large window of ages (P21 to P92) (McCutcheon and Marinelli, 2009; Sengupta, 2013). There is some discrepancy between these mRNA results and previous studies focusing on the G_αo_ protein levels during this phase of development. In these, G_αo_ protein levels were low at P1 and drastically increased during the first weeks of development until around day 25, where they achieved adult levels (Asano et al., 1988; Chang et al., 1988). The differences could result from a lag in the processing of the G_αo_ mRNA that affects the detection of the G_αo_ protein early in development (Duman et al., 1989). On the other hand, they could be due to alterations in the rate of G_αo_ protein turnover during development, with G_αo_ exhibiting a higher turnover rate at early stages of development, and a slower and more stable turnover as the brain matures. This second hypothesis was corroborated by follow up results demonstrating a slower G_αo_ protein turnover with neuronal differentiation (Brabet et al., 1991). Later studies also showed brain region-specificities of G_αo_ levels. In rat cortex and thalamus, the protein levels of G_αo1_ (isoform 1 of G_αo_) slightly rise on the first postnatal days and then stabilize until adulthood (P90), while in the pituitary gland and in hippocampus, G_αo1_ levels significantly rise on the first 10–20 days and then start to slowly decrease until P90 (Ihnatovych et al., 2002).

The immunoreactivity of G proteins in the human brain has also been assessed in post-mortem brains of human individuals with ages ranging from 3 days to 92 years (Young et al., 1991). In the whole brain, G_αi_ and G_αs_ (52 kDa form) immunoreactivities were observed to significantly decrease with age. G_αo_ also showed a tendency to decrease with age, although not statistically significant. G_β_ immunoreactivity presented no alterations with age, while G_αs_ (45 kDa form) tended to increase. Specific evaluation of G_αo_ in the pre-frontal cortex of human post-mortem brains, however, revealed a more significant correlation between aging and decreased G_αo_ immunoreactivity (Li et al., 1996). Interestingly, neither G_αi_ nor G_αs_ exhibited major alterations with aging in this brain area. Of note, the differences between both studies can result not only from the different brain region analyzed, but also from some interindividual variability in the studied samples (Li et al., 1996). A later study showed a decrease in both G_αo_ (slight) and G_αi_ (significant) levels in the pre-frontal cortex with aging (González-Maeso et al., 2002), with the differential results between studies potentially residing in the range of ages represented in each study. While the Young et al. (1991) and González-Maeso et al. (2002) studies used samples from individuals with ages ranging from 3 days to 92-years-old and 1 to 88-years-old, respectively, the Li et al. (1996) study used samples from individuals with ages ranging from 19 to 100-years-old (Young et al., 1991; Li et al., 1996; González-Maeso et al., 2002). The lack of samples from younger individuals in the Li et al. (1996) study and/or its older individuals might explain the absence of alterations in G_αi_ levels, as well as the detection of a significant decrease in G_αo_ levels.

The Li et al. (1996) study also compared control individuals to individuals with Alzheimer’s Disease (AD), but no significant differences were detected in G proteins levels besides a slight decrease in G_αi1_ (Li et al., 1996). These results are in accordance with previous works that also showed no alterations in G_αo_ levels in different regions of AD’s brains (McLaughlin et al., 1991; O’Neill et al., 1994), with more recent studies also confirming these results (Hashimoto et al., 2004). Despite the lack of significant changes in G_αo_ levels in AD brains, G_αo_ signaling was found deregulated in AD patients. Forms of amyloid precursor protein (APP) with familial AD (FAD)-associated V642 mutations, such as V642 to I (London FAD mutation), F (Indiana FAD mutation) and to G (no FAD associated), have an increased capability to activate G_αo_, with this activation resulting in apoptosis and associated DNA fragmentation (Okamoto et al., 1995; Yamatsuji et al., 1996a,b). APP has also been shown to induce cell death via a mechanism involving both G_αo_ and Src activation (Sudo et al., 2000; Xu et al., 2009). There are also some reports studying a possible link between G_αo_ and the Alzheimer’s amyloid-β peptide (Aβ), with mixed results. While some reports show that Aβ does not participate in the APP-G_αo_ induced neuronal death (Sudo et al., 2001), others demonstrated that Aβ toxicity in neuronal cells is dependent on APP binding to G_αo_ (via deletion studies of the G_αo_ binding sequence) and G_αo_ activity (via cells exposure to the G_αo_ inhibitor pertussis toxin, PTX) (Shaked et al., 2009; Sola Vigo et al., 2009). These differences might be due to the different experimental setups. The Xu study intended to evaluate if Aβ mediated the neurotoxic effect of APP upon its “activation” with specific antibodies, thus placing Aβ downstream of APP G_αo_ interaction, while the Sola Vigo and Shaked studies placed Aβ upstream the APP-G_αo_ interaction.

G_αo_ alterations have also been associated with other neurological disorders. Although not age-related, these highlight the role of G_αo_ in the brain. Briefly, the levels of Go in its trimeric form are increased in Bipolar Affective Disorder brains when compared to control individuals (Friedman and Wang, 1996); both G_αo_ mRNA and protein levels are decreased in the prefrontal cortex of adult suicide victims, while in teenagers the G_αo_ decrease is only detected in subjects with a history of mental illness (Dwivedi et al., 2002).

Taking together, these studies indicate that G_αo_ expression in the human brain changes during brain development and aging, and that these alterations vary between different brain regions. Moreover, while alterations in G_αo_ levels do not seem to be associated with AD, a deregulation of G_αo_ signaling might play an important role in this age-related disease.

## Considerations on Age-Related Studies on GPCRs and Coupled G Proteins

Although the studies here analyzed were generally consistent in relation to an age-induced decreased in various GPCRs densities, some studies reported no alterations or even an increase in the same receptors’ densities. Differences between studies may be related to the specific brain region studied (already addressed in our approach to the GPCRs and G proteins studies), experimental methodologies used, differences in species/strains/individuals, age and gender/sex of the studied individuals.

Regarding the methodologies, several studies used ligands that have different affinities for the receptors; further, these ligands were sometimes non-selective or only slightly selective to the subtype of GPCR (Arranz et al., 1993; Kaasinen et al., 2000; Tayebati et al., 2001, 2002; Matuskey et al., 2016; Lebois et al., 2018; Takkinen et al., 2018). Radioisotope ligand-binding assays may also present some limitations. For example, the most used isotope, ^3^H, emits β-particles of low energy that are differently absorbed by tissues (Lidow et al., 1988). By its turn ^125^I emits high energy particles that reduce the method’s resolution (Lidow et al., 1988; Han et al., 1989). The receptors’ levels were also analyzed by different techniques. The GPCRs’ mRNA was assessed by Northern blot, or by the highly sensitive polymerase chain reaction (qPCR) technique, useful when only small amounts of tissue are available and when the target transcript is of low abundance (Burnet et al., 1994). Importantly, mRNA levels not always coincide with protein ones; as proteins are the GPCRs functional molecules, we have here placed a main focus on GPCRs’ protein levels (Simonyi et al., 2000; Yudin and Rohacs, 2018). Studies in *postmortem* tissues also have some restrictions since delays and the freezing storage process (temperature and tissue storage duration) may alter the integrity of receptors’ mRNA and protein (Mato and Pazos, 2004).

Additionally, changes in the aging brain may vary between species, strains and even individuals (Rinne et al., 1990; Ekonomou et al., 2000; Yudin and Rohacs, 2018). Studies have shown that humans and rhesus macaques have diverged from mice due to a marked increase in age-dependent down-regulation of neuronal genes’ expression (Loerch et al., 2008). This is an evolved feature of the human (and macaque) aging brain that might alter neural network and contribute to age-related cognitive changes (Loerch et al., 2008). Despite common neurodevelopmental processes in mammals, only a small subset of age-related alterations in gene expression are conserved from mouse to man (Loerch et al., 2008). In this way, experimental animals cannot fully model human, with the evolutionary distance between species being a limitation in scientific research (Loerch et al., 2008; Silbereis et al., 2016). On the other hand, morphological changes in the aging brain also depend on the age of individuals (Ekonomou et al., 2000). Studies of the biology of the aging brain using comparative survival curves for humans, mice, and rhesus monkey, suggest that a 30-month-old mouse is similar to an 81-year-old human and a 30-year-old rhesus monkey is similar to a 70-year-old human (Loerch et al., 2008). So, some contradictory reported results may be ascribed to differences in the age of the animals used, and possibly some age-related alterations might have been underestimated due to such differences. Also, it is important to consider sample size, as lower values will translate into low statistical power and can cause discrepancies in studies. In techniques such as molecular imaging, this is probably due to its high monetary cost and to the desire to limit radiation exposure in healthy volunteers (Karrer et al., 2017; Takkinen et al., 2018).

A clear comprehension on how gender/sex and age affect (neuro)pathologies is very important, and these two factors should be included as selection criteria or experimental parameters in the design and interpretation of this type of studies (Pandya et al., 2019). Regarding age, many studies have used only ‘under-aged’ subjects, such as rats and mice aged 24 months or even less [e.g., the oldest mice used in Yew et al. (2009) study were 12-months-old], or human individuals with ages ranging, e.g., from 22 to 53 years old (Li et al., 1996; Tauscher et al., 2001; Yew et al., 2009). In many studies, statistical significance was only obtained in 30-month-old rats but not in 24-month-old rats, which may justify contradictory results (Meyer et al., 2007). Gender/sex can also influence the density and affinity of GPCRs. Indeed, it appears to exist a gender/sex influence on the control of the aging mechanisms, and in the structure and function of the CNS, including in the synaptic alterations with aging and age-related pathologies (Messing et al., 1981; Palego et al., 1997). Some studies have addressed and revealed these gender/sex-specific alterations in GPCRs levels and function in animals and human samples (Supplementary Table S2).

It is thus important in the future to pay attention to these variables, and increase the number of human studies covering a good age interval and differentiating between both genders, in order to minimize discrepancies in results and make it easier to draw reliable conclusions on age-related GPCRs alterations in the human brain.

## Conclusion

**FIGURE 4:**
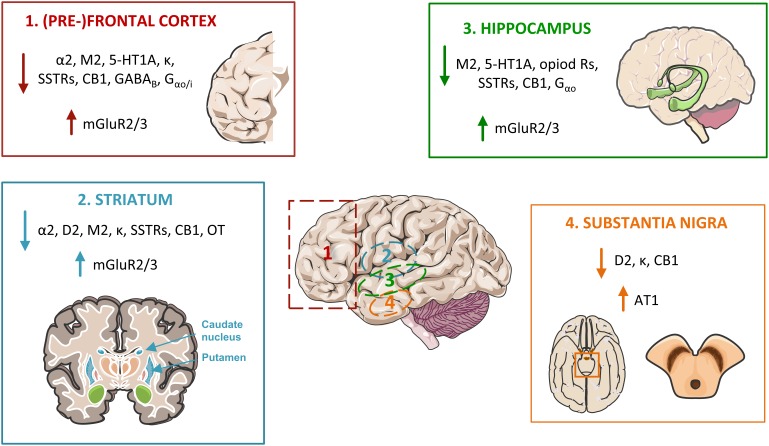
Major alterations in G_o/i_-coupled GPCRs’ pathways in the aged brain, affecting four main areas. In the frontal lobe, particularly at the pre-frontal cortex **(1)**, the protein densities and/or affinity of α2 adrenoceptors, acetylcholine M2 receptors (M2), serotonin 5-HT1A receptors, opioid receptors (including the kappa receptor, κ), somatostatin receptors (SSTRs), cannabinoid receptor CB1, the metabotropic GABA_B_ receptor, and of the Gα proteins G_αo/i_, are found decreased. The same occurs in the **(2)** striatum, excluding the serotonin 5-HT1A receptors, the GABA metabotropic receptors and Gαo/i, and including dopamine D2 receptors and the oxytocin (OT) receptors. The **(3)** hippocampus also presents decreases in the same receptors as the frontal cortex, with the exclusion of the adrenoceptors and the GABA_B_ receptor. Contrarily, mGluR2 and 3 glutamate receptors are up-regulated in these three brain areas. In the **(4)** substantia nigra, dopamine D2, opioid κ and cannabinoid CB1 receptors are also depleted with age, while angiotensin AT1 receptor levels are increased.

This review shows that the main age-related hallmark of G_i/o_-coupled GPCRs is their decreased brain densities (and associated binding potential), found in roughly two thirds of the subtypes of receptors studied. This may result in loss of function, particularly in brain regions where decreased density is associated with altered binding/agonist association, and/or decreased G protein levels or downstream signaling. Further, due to formation of GPCR dimers at the membrane, the decreased density of a GPCR may strongly affect the efficiency and the signaling pathway of another GPCR (and the pharmacological treatment to be applied). Some brain regions are more affected than others, including (1) the cortex, particularly the frontal cortex but also the temporal and other cortex lobes, (2) the striatum (caudate, putamen, nucleus accumbens), (3) substantia nigra, and (4) the hippocampus. These brain regions particularly sensitive to aging are related to functions such as learning, planning and execution, memory, and motor control. Homeostatic functions, neuromodulation and neuroprotection via adenosine and oxytocin receptors also decrease with age. On the other hand, receptors related to inflammation, oxidative stress and excitotoxicity have their densities increased with aging (Figure 4).

Since GPCRs are the largest family of transmembrane receptors in humans and regulate several physiological functions, and given that the heterotrimeric G_i/o_ family is the most abundant in brain, these alterations significantly impair signal transduction and lead to (or aid on) age-related senescence processes and neuropathologies. With an increased aged population, the G_i/o_-coupled GPCRs signaling pathways are thus to be seriously considered as targets to attempt to revert the decline in cognitive and motor functions associated with senescence, as well as increased sensitivity to neurodegeneration at advanced ages.

## Author Contributions

PO and MR performed the data mining regarding GPCRs, under the supervision of RD and SV. RD collected the data regarding the heterotrimeric G proteins. PO, MR, RD, and SV wrote and corrected the manuscript. AA, a clinician with expertise in human anatomy, has helped to categorize the data regarding brain regions and reviewed the manuscript.

## Conflict of Interest Statement

The authors declare that the research was conducted in the absence of any commercial or financial relationships that could be construed as a potential conflict of interest.

## References

[B1] Abush H. Akirav I. (2009). Cannabinoids modulate hippocampal memory and plasticity. *Hippocampus* 20 1126–1138. 10.1002/hipo.20711 19830813

[B2] Albasanz J. L. Perez S. Barrachina M. Ferrer I. Martín M. (2008). Up-regulation of adenosine receptors in the frontal cortex in Alzheimer’s disease. *Brain Pathol.* 18 211–219. 10.1111/j.1750-3639.2007.00112.x 18241242 PMC8095610

[B3] Alemany R. Perona J. S. Sánchez-Dominguez J. M. Montero E. Cañizares J. Bressani R. (2007). G protein-coupled receptor systems and their lipid environment in health disorders during aging. *Biochim. Biophys. Acta Biomembr.* 1768 964–975. 10.1016/j.bbamem.2006.09.024 17070497

[B4] Alexander S. P. Christopoulos A. Davenport A. P. Kelly E. Marrion N. V. Peters J. A. (2017). The concise guide to pharmacology 2017/18: G protein-coupled receptors. *Br. J. Pharmacol.* 174(Suppl.), S17–S129. 10.1111/bph.13878 29055040 PMC5650667

[B5] Amenta F. Mignini F. Ricci A. Sabbatini M. Tomassoni D. Tayebati S. K. (2001). Age-related changes of dopamine receptors in the rat hippocampus: a light microscope autoradiography study. *Mech. Ageing Dev.* 122 2071–2083. 10.1016/S0047-6374(01)00317-7 11589924

[B6] Angulo E. Casadó V. Mallol J. Canela E. I. Viñals F. Ferrer I. (2003). A1 adenosine receptors accumulate in neurodegenerative structures in Alzheimer disease and mediate both amyloid precursor protein processing and tau phosphorylation and translocation. *Brain Pathol.* 13 440–451. 10.1111/j.1750-3639.2003.tb00475.x 14655750 PMC8095992

[B7] Antonini A. Leenders L. K. Antonini A. Leenders K. L. Reist H. Thomann R. (1993). Effect of age on D2 dopamine receptors in normal human brain measured by positron emission tomography and 11C-raclopride. *Arch. Neurol.* 50 474–480. 10.1001/archneur.1993.00540050026010 8489403

[B8] Araki T. Kato H. Shuto K. Fujiwara T. Itoyama Y. (1997). Effect of aging on dopaminergic receptors and uptake sites in the rat brain studied by receptor autoradiography. *J. Neurol. Sci.* 148 131–137. 10.1016/S0022-510X(96)05343-9 9129108

[B9] Arango V. Underwood M. D. Mann J. J. (1992). Alterations in monoamine receptors in the brain of suicide victims. *J. Clin. Psychopharmacol.* 12 8S–12S. 10.1097/00004714-199204001-00002 1315808

[B10] Arranz B. Eriksson A. Mellerup E. Plenge P. Marcusson J. (1993). Effect of aging in human cortical pre- and postsynaptic serotonin binding sites. *Brain Res.* 620 163–166. 10.1016/0006-8993(93)90286-V 8402192

[B11] Arsenijevic Y. Dreifuss J. J. Vallet P. Marguerat A. Tribollet E. (1995). Reduced binding of oxytocin in the rat brain during aging. *Brain Res.* 698 275–279. 10.1016/0006-8993(95)01020-V 8581497

[B12] Arthofer E. Hot B. Petersen J. Strakova K. Jäger S. Grundmann M. (2016). WNT Stimulation Dissociates a Frizzled 4 Inactive-State Complex with Gα12/13. *Mol. Pharmacol.* 90 447–459. 10.1124/mol.116.104919 27458145 PMC5034691

[B13] Asano T. Kamiya N. Semba R. Kato K. (1988). Ontogeny of the GTP-binding protein Go in rat brain and heart. *J. Neurochem.* 51 1711–1716. 10.1111/j.1471-4159.1988.tb01149.x 3141585

[B14] Baik J.-H. (2013). Dopamine signaling in reward-related behaviors. *Front. Neural Circuits* 7:152. 10.3389/fncir.2013.00152 24130517 PMC3795306

[B15] Baltoumas F. A. Theodoropoulou M. C. Hamodrakas S. J. (2013). Interactions of the α-subunits of heterotrimeric G-proteins with GPCRs, effectors and RGS proteins: a critical review and analysis of interacting surfaces, conformational shifts, structural diversity and electrostatic potentials. *J. Struct. Biol.* 182 209–218. 10.1016/j.jsb.2013.03.004 23523730

[B16] Barense M. D. Fox M. T. Baxter M. G. (2002). Aged rats are impaired on an attentional set-shifting task sensitive to medial frontal cortex damage in young rats. *Learn. Mem.* 9 191–201. 10.1101/lm.48602 12177232 PMC182583

[B17] Barg J. Belcheva M. Rowinski J. Ho A. Burke W. J. Chung H. D. (1993). Opioid receptor density changes in Alzheimer amygdala and putamen. *Brain Res.* 632 209–215. 10.1016/0006-8993(93)91155-L 8149229

[B18] Benigni A. Corna D. Zoja C. Sonzogni A. Latini R. Salio M. (2009). Disruption of the Ang II type 1 receptor promotes longevity in mice. *J. Clin. Invest.* 119 524–530. 10.1172/JCI36703 19197138 PMC2648681

[B19] Bigham M. H. Lidow M. S. (1995). Adrenergic and serotonergic receptors in aged monkey neocortex. *Neurobiol. Aging* 16 91–104. 10.1016/0197-4580(95)80012-G 7723941

[B20] Bikkavilli R. K. Feigin M. E. Malbon C. C. (2008). G alpha o mediates WNT-JNK signaling through dishevelled 1 and 3, RhoA family members, and MEKK 1 and 4 in mammalian cells. *J. Cell Sci.* 121 234–245. 10.1242/jcs.021964 18187455

[B21] Birnbaumer L. (2007). Expansion of signal transduction by G proteins. *Biochim. Biophys. Acta Biomembr.* 1768 772–793. 10.1016/j.bbamem.2006.12.002 17258171 PMC1993906

[B22] Bjarnadóttir T. K. Fredriksson R. Höglund P. J. Gloriam D. E. Lagerström M. C. Schiöth H. B. (2004). The human and mouse repertoire of the adhesion family of G-protein-coupled receptors. *Genomics* 84 23–33. 10.1016/j.ygeno.2003.12.004 15203201

[B23] Blake M. J. Appel N. M. Joseph J. A. Stagg C. A. Anson M. de Souza E. B. (1991). Muscarinic acetylcholine receptor subtype mRNA expression and ligand binding in the aged rat forebrain. *Neurobiol. Aging* 12 193–199. 10.1016/0197-4580(91)90097-4 1876225

[B24] Brabet P. Pantaloni C. Bockaert J. Homburger V. (1991). Metabolism of two Go alpha isoforms in neuronal cells during differentiation. *J. Biol. Chem.* 266 12825–12828. 1906458

[B25] Branch S. Y. Chen C. Sharma R. Lechleiter J. D. Li S. Beckstead M. J. (2016). Dopaminergic neurons exhibit an age-dependent decline in electrophysiological parameters in the mitopark mouse model of Parkinson’s disease. *J. Neurosci.* 36 4026–4037. 10.1523/JNEUROSCI.1395-15.2016 27053209 PMC4821912

[B26] Brosnan J. T. Brosnan M. E. (2013). Glutamate: a truly functional amino acid. *Amino Acids* 45 413–418. 10.1007/s00726-012-1280-4 22526238

[B27] Burgos-Ramos E. Hervás-Aguilar A. Aguado-Llera D. Puebla-Jiménez L. Hernández-Pinto A. M. Barrios V. (2008). Somatostatin and Alzheimer’s disease. *Mol. Cell. Endocrinol.* 286 104–111. 10.1016/j.mce.2008.01.014 18359553

[B28] Burnet P. W. Eastwood S. L. Harrison P. J. (1994). Detection and quantitation of 5-HT1A and 5-HT2A receptor mRNAs in human hippocampus using a reverse transcriptase-polymerase chain reaction (RT-PCR) technique and their correlation with binding site densities and age. *Neurosci. Lett.* 178 85–89. 10.1016/0304-3940(94)90296-8 7529388

[B29] Burnstock G. Fredholm B. B. Verkhratsky A. (2011). Adenosine and ATP receptors in the brain. *Curr. Top. Med. Chem.* 11 973–1011. 10.2174/156802611795347627 21401499

[B30] Butzlaff M. Ponimaskin E. (2016). The role of serotonin receptors in Alzheimer’ s disease. *Opera Med. Physiol.* 1 91–100.

[B31] Calebiro D. Koszegi Z. (2019). The subcellular dynamics of GPCR signaling. *Mol. Cell. Endocrinol.* 483 24–30. 10.1016/j.mce.2018.12.020 30610913

[B32] Canas P. M. Duarte J. M. N. Rodrigues R. J. Köfalvi A. Cunha R. A. (2009). Modification upon aging of the density of presynaptic modulation systems in the hippocampus. *Neurobiol. Aging* 30 1877–1884. 10.1016/j.neurobiolaging.2008.01.003 18304697

[B33] Caraci F. Nicoletti F. Copani A. (2018). Metabotropic glutamate receptors: the potential for therapeutic applications in Alzheimer’s disease. *Curr. Opin. Pharmacol.* 38 1–7. 10.1016/j.coph.2017.12.001 29278824

[B34] Carbe C. J. Cheng L. Addya S. Gold J. I. Gao E. Koch W. J. (2014). Gi proteins mediate activation of the canonical hedgehog pathway in the myocardium. *Am. J. Physiol. Heart Circ. Physiol.* 307 H66–H72. 10.1152/ajpheart.00166.2014 24816261 PMC4080174

[B35] Carpenter B. Nehmé R. Warne T. Leslie A. G. W. Tate C. G. (2016). Structure of the adenosine A(2A) receptor bound to an engineered G protein. *Nature* 536 104–107. 10.1038/nature18966 27462812 PMC4979997

[B36] Carretero J. Bodego P. Rodríguez R. E. Rubio M. Blanco E. Burks D. J. (2004). Expression of the mu-opioid receptor in the anterior pituitary gland is influenced by age and sex. *Neuropeptides* 38 63–68. 10.1016/j.npep.2004.01.004 15223267

[B37] Caspary D. M. Ling L. Turner J. G. Hughes L. F. (2008). Inhibitory neurotransmission, plasticity and aging in the mammalian central auditory system. *J. Exp. Biol.* 211 1781–1791. 10.1242/jeb.013581 18490394 PMC2409121

[B38] Chang K. J. Pugh W. Blanchard S. G. McDermed J. Tam J. P. (1988). Antibody specific to the alpha subunit of the guanine nucleotide-binding regulatory protein Go: developmental appearance and immunocytochemical localization in brain. *Proc. Natl. Acad. Sci. U.S.A.* 85 4929–4933. 10.1073/pnas.85.13.4929 3133664 PMC280551

[B39] Chen J.-F. Lee C. Chern Y. (2014). Adenosine receptor neurobiology: overview. *Int. Rev. Neurobiol.* 119 1–49. 10.1016/B978-0-12-801022-8.00001-5 25175959

[B40] Chen W. Hu L. A. Semenov M. V. Yanagawa S. Kikuchi A. Lefkowitz R. J. (2001). beta-Arrestin1 modulates lymphoid enhancer factor transcriptional activity through interaction with phosphorylated dishevelled proteins. *Proc. Natl. Acad. Sci. U.S.A.* 98 14889–14894. 10.1073/pnas.211572798 11742073 PMC64954

[B41] Cheng J. T. Liu I. M. Juang S. W. Jou S. B. (2000). Decrease of adenosine A-1 receptor gene expression in cerebral cortex of aged rats. *Neurosci. Lett.* 283 227–229. 10.1016/S0304-3940(00)00961-7 10754229

[B42] Cheng L. Al-Owais M. Covarrubias M. L. Koch W. J. Manning D. R. Peers C. (2018). Coupling of Smoothened to inhibitory G proteins reduces voltage-gated K+ currents in cardiomyocytes and prolongs cardiac action potential duration. *J. Biol. Chem.* 293 11022–11032. 10.1074/jbc.RA118.001989 29802197 PMC6052211

[B43] Chu Z. Galarreta M. Hestrin S. (2003). Synaptic interactions of late-spiking neocortical neurons in layer 1. *J. Neurosci.* 23 96–102. 10.1523/JNEUROSCI.23-01-00096.2003 12514205 PMC6742162

[B44] Chu Sin Chung P. Kieffer B. L. (2013). Delta opioid receptors in brain function and diseases. *Pharmacol. Ther.* 140 112–120. 10.1016/j.pharmthera.2013.06.003 23764370 PMC3775961

[B45] Civelli O. (2005). Orphan GPCRs in the regulation of sleep and circadian rhythm. *FEBS J.* 272 5673–5674. 10.1111/j.1742-4658.2005.04867.x 16279932

[B46] Civelli O. (2012). Orphan GPCRs and Neuromodulation. *Neuron* 76 12–21. 10.1016/j.neuron.2012.09.009 23040803 PMC3474844

[B47] Cunha R. A. Constantino M. C. Sebastião A. M. Ribeiro J. A. (1995). Modification of A1 and A2a adenosine receptor binding in aged striatum, hippocampus and cortex of the rat. *Neuroreport* 6 1583–1588. 10.1097/00001756-199507310-00029 7579154

[B48] Dang L. C. Castrellon J. J. Perkins S. F. Le N. T. Cowan R. L. Zald D. H. (2017). Reduced effects of age on dopamine D2 receptor levels in physically active adults. *Neuroimage* 148 123–129. 10.1016/j.neuroimage.2017.01.018 28089678 PMC5344739

[B49] De Keyser J. Ebinger G. Vauquelin G. (1990). Age-related changes in the human nigrostriatal dopaminergic system. *Ann. Neurol.* 27 157–161. 10.1002/ana.410270210 2107785

[B50] De Vries L. Zheng B. Fischer T. Elenko E. Farquhar M. G. (2000). The regulator of G protein signaling family. *Annu. Rev. Pharmacol. Toxicol.* 40 235–271. 10.1146/annurev.pharmtox.40.1.235 10836135

[B51] Desai A. J. Miller L. J. (2018). Changes in the plasma membrane in metabolic disease: impact of the membrane environment on G protein-coupled receptor structure and function. *Br. J. Pharmacol.* 175 4009–4025. 10.1111/bph.13943 28691227 PMC6177615

[B52] Dillon K. A. Gross-Isseroff R. Israeli M. Biegon A. (1991). Autoradiographic analysis of serotonin 5-HT1A receptor binding in the human brain postmortem: effects of age and alcohol. *Brain Res.* 554 56–64. 10.1016/0006-8993(91)90171-Q 1834306

[B53] Diniz D. A. Petrocchi J. A. Navarro L. C. Souza T. C. Castor M. G. M. Perez A. C. (2015). Serotonin induces peripheral mechanical antihyperalgesic effects in mice. *Eur. J. Pharmacol.* 767 94–97. 10.1016/j.ejphar.2015.10.012 26460149

[B54] Doder M. Rabiner E. A. Turjanski N. Lees A. J. Brooks D. J. 11C-Way 100635 Pet study. (2003). Tremor in Parkinson’s disease and serotonergic dysfunction: an 11C-WAY 100635 PET study. *Neurology* 60 601–605. 10.1212/01.WNL.0000031424.51127.2B 12601099

[B55] Dong X. Han S. Zylka M. J. Simon M. I. Anderson D. J. (2001). A diverse family of GPCRs expressed in specific subsets of nociceptive sensory neurons. *Cell* 106 619–632. 10.1016/S0092-8674(01)00483-4 11551509

[B56] Duman R. S. Saito N. Tallman J. F. (1989). Development of beta-adrenergic receptor and G protein messenger RNA in rat brain. *Brain Res. Mol. Brain Res.* 5 289–296. 10.1016/0169-328X(89)90063-6 2546000

[B57] Duncan M. J. Hensler J. G. (2002). Aging alters in a region-specific manner serotonin transporter sites and 5-HT(1A) receptor-G protein interactions in hamster brain. *Neuropharmacology* 43 36–44. 10.1016/S0028-3908(02)00072-2 12213257

[B58] Durdagi S. Erol I. Salmas R. E. Aksoydan B. Kantarcioglu I. (2018). Oligomerization and cooperativity in GPCRs from the perspective of the angiotensin AT1 and dopamine D2 receptors. *Neurosci. Lett.* 10.1016/j.neulet.2018.04.028 [Epub ahead of print]. 29684528

[B59] Dwivedi Y. Rizavi H. S. Conley R. R. Roberts R. C. Tamminga C. A. Pandey G. N. (2002). mRNA and protein expression of selective alpha subunits of G proteins are abnormal in prefrontal cortex of suicide victims. *Neuropsychopharmacology* 27:499. 10.1016/S0893-133X(02)00335-4 12377388

[B60] Ekonomou A. Pagonopoulou O. Angelatou F. (2000). Age-dependent changes in adenosine A1 receptor and uptake site binding in the mouse brain: an autoradiographic study. *J. Neurosci. Res.* 60 257–265. 10.1002/(SICI)1097-4547(20000415)60:2<257::AID-JNR15>3.0.CO;2-U 10740231

[B61] Ferraguti F. Shigemoto R. (2006). Metabotropic glutamate receptors. *Cell Tissue Res.* 326 483–504. 10.1007/s00441-006-0266-5 16847639

[B62] Ferré S. (2015). The GPCR heterotetramer: challenging classical pharmacology. *Trends Pharmacol. Sci.* 36 145–152. 10.1016/j.tips.2015.01.002 25704194 PMC4357316

[B63] Ferre S. Casado V. Devi L. A. Filizola M. Jockers R. Lohse M. J. (2014). G protein-coupled receptor oligomerization revisited: functional and pharmacological perspectives. *Pharmacol. Rev.* 66 413–434. 10.1124/pr.113.008052 24515647 PMC3973609

[B64] Fredholm B. B. Johansson B. Lindström K. Wahlström G. (1998). Age-dependent changes in adenosine receptors are not modified by life-long intermittent alcohol administration. *Brain Res.* 791 177–185. 10.1016/S0006-8993(98)00090-0 9593882

[B65] Fredriksson R. Lagerström M. C. Lundin L.-G. Schiöth H. B. (2003). The G-protein-coupled receptors in the human genome form five main families. Phylogenetic analysis, paralogon groups, and fingerprints. *Mol. Pharmacol.* 63 1256–1272. 10.1124/mol.63.6.1256 12761335

[B66] Freeman G. B. Gibson G. E. (1987). Selective alteration of mouse brain neurotransmitter release with age. *Neurobiol. Aging* 8 147–152. 10.1016/0197-4580(87)90024-8 2884583

[B67] Friedman E. Wang H. Y. (1996). Receptor-mediated activation of G proteins is increased in postmortem brains of bipolar affective disorder subjects. *J. Neurochem.* 67 1145–1152. 10.1046/j.1471-4159.1996.67031145.x 8752121

[B68] Gahete M. D. Rubio A. Durán-Prado M. Avila J. Luque R. M. Castaño J. P. (2010). Expression of Somatostatin, cortistatin, and their receptors, as well as dopamine receptors, but not of neprilysin, are reduced in the temporal lobe of Alzheimer’s disease patients. *J. Alzheimers. Dis.* 20 465–475. 10.3233/JAD-2010-1385 20164562

[B69] Ghosh P. Rangamani P. Kufareva I. (2017). The GAPs, GEFs, GDIs and…now, GEMs: new kids on the heterotrimeric G protein signaling block. *Cell Cycle* 16 607–612. 10.1080/15384101.2017.1282584 28287365 PMC5397260

[B70] Gimpl G. Fahrenholz F. (2001). The oxytocin receptor system: structure, function, and regulation. *Physiol. Rev.* 81 629–683. 10.1152/physrev.2001.81.2.629 11274341

[B71] Gonzalez B. Leroux P. Lamacz M. Bodenant C. Balazs R. Vaudry H. (1992). Somatostatin receptors are expressed by immature cerebellar granule cells: evidence for a direct inhibitory effect of somatostatin on neuroblast activity. *Proc. Natl. Acad. Sci. U.S.A.* 89 9627–9631. 10.1073/pnas.89.20.9627 1357666 PMC50185

[B72] González S. Moreno-Delgado D. Moreno E. Pérez-Capote K. Franco R. Mallol J. (2012). Circadian-related heteromerization of adrenergic and dopamine D4 receptors modulates melatonin synthesis and release in the pineal gland. *PLoS Biol.* 10:e1001347. 10.1371/journal.pbio.1001347 22723743 PMC3378626

[B73] González-Maeso J. Torre I. Rodríguez-Puertas R. García-Sevilla J. A. Guimón J. Meana J. J. (2002). Effects of age, postmortem delay and storage time on receptor-mediated activation of G-proteins in human brain. *Neuropsychopharmacology* 26 468–478. 10.1016/S0893-133X(01)00342-6 11927171

[B74] Gouveia M. Xia K. Colón W. Vieira S. I. Ribeiro F. (2017). Protein aggregation, cardiovascular diseases, and exercise training: where do we stand? *Ageing Res. Rev.* 40 1–10. 10.1016/j.arr.2017.07.005 28757291

[B75] Grauschopf U. Lilie H. Honold K. Wozny M. Reusch D. Esswein A. (2000). The N-terminal fragment of human parathyroid hormone receptor 1 constitutes a hormone binding domain and reveals a distinct disulfide pattern. *Biochemistry* 39 8878–8887. 10.1021/bi0001426 10913300

[B76] Grigorieff N. Ceska T. A. Downing K. H. Baldwin J. M. Henderson R. (1996). Electron-crystallographic refinement of the structure of bacteriorhodopsin. *J. Mol. Biol.* 259 393–421. 10.1006/jmbi.1996.0328 8676377

[B77] Grumolato L. Liu G. Mong P. Mudbhary R. Biswas R. Arroyave R. (2010). Canonical and noncanonical Wnts use a common mechanism to activate completely unrelated coreceptors. *Genes Dev.* 24 2517–2530. 10.1101/gad.1957710 21078818 PMC2975928

[B78] Gudermann T. Kalkbrenner F. Schultz G. (1996). Diversity and selectivity of receptor-G protein interaction. *Annu. Rev. Pharmacol. Toxicol.* 36 429–459. 10.1146/annurev.pa.36.040196.002241 8725397

[B79] Guerram M. Zhang L.-Y. Jiang Z.-Z. (2016). G-protein coupled receptors as therapeutic targets for neurodegenerative and cerebrovascular diseases. *Neurochem. Int.* 101 1–14. 10.1016/j.neuint.2016.09.005 27620813

[B80] Guo D. F. Sun Y. L. Hamet P. Inagami T. (2001). The angiotensin II type 1 receptor and receptor-associated proteins. *Cell Res.* 11 165–180. 10.1038/sj.cr.7290083 11642401

[B81] Gupta V. Bhandari D. Leyme A. Aznar N. Midde K. K. Lo I.-C. (2016). GIV/Girdin activates Gαi and inhibits Gαs via the same motif. *Proc. Natl. Acad. Sci. U.S.A.* 113 E5721–E5730. 10.1073/pnas.1609502113 27621449 PMC5047194

[B82] Gurevich V. V. Gurevich E. V. (2017). Molecular mechanisms of GPCR signaling: a structural perspective. *Int. J. Mol. Sci.* 18:E2519. 10.3390/ijms18122519 29186792 PMC5751122

[B83] Hamilton C. A. Howe C. A. Reid J. L. (1984). Changes in brain alpha-adrenoceptors with increasing age in rabbits. *Brain Res.* 322 177–179. 10.1016/0006-8993(84)91201-0 6097335

[B84] Han Y. Moreira I. S. Urizar E. Weinstein H. Javitch J. A. (2009). Allosteric communication between protomers of dopamine class A GPCR dimers modulates activation. *Nat. Chem. Biol.* 5 688–695. 10.1038/nchembio.199 19648932 PMC2817978

[B85] Han Z. Kuyatt B. L. Kochman K. A. DeSouza E. B. Roth G. S. (1989). Effect of aging on concentrations of D2-receptor-containing neurons in the rat striatum. *Brain Res.* 498 299–307. 10.1016/0006-8993(89)91108-6 2529020

[B86] Hashimoto E. Ozawa H. Saito T. Gsell W. Takahata N. Riederer P. (2004). Impairment of G(salpha) function in human brain cortex of Alzheimer’s disease: comparison with normal aging. *J. Neural Transm.* 111 311–322. 10.1007/s00702-003-0089-4 14991457

[B87] Hauser A. S. Attwood M. M. Rask-Andersen M. Schiöth H. B. Gloriam D. E. (2017). Trends in GPCR drug discovery: new agents, targets and indications. *Nat. Rev. Drug Discov.* 16 829–842. 10.1038/nrd.2017.178 29075003 PMC6882681

[B88] He X. Li F. Chang W.-P. Tang J. (2005). GGA proteins mediate the recycling pathway of memapsin 2 (BACE). *J. Biol. Chem.* 280 11696–11703. 10.1074/jbc.M411296200 15615712

[B89] Heng B. C. Aubel D. Fussenegger M. (2013). An overview of the diverse roles of G-protein coupled receptors (GPCRs) in the pathophysiology of various human diseases. *Biotechnol. Adv.* 31 1676–1694. 10.1016/j.biotechadv.2013.08.017 23999358

[B90] Henry J. M. Filburn C. R. Joseph J. A. Roth G. S. (1986). Effect of aging on striatal dopamine receptor subtypes in Wistar rats. *Neurobiol. Aging* 7 357–361. 10.1016/0197-4580(86)90162-4 2946969

[B91] Hess G. D. Joseph J. A. Roth G. S. (1981). Effect of age on sensitivity to pain and brain opiate receptors. *Neurobiol. Aging* 2 49–55. 10.1016/0197-4580(81)90059-2 6267493

[B92] Hiller J. M. Fan L. Q. Simon E. J. (1992). Age-related changes in kappa opioid receptors in the guinea-pig brain: a quantitative autoradiographic study. *Neuroscience* 50 663–673. 10.1016/0306-4522(92)90455-B 1331867

[B93] Ho Wei L. Arastoo M. Georgiou I. Manning D. R. Riobo-Del Galdo N. A. (2018). Activation of the Gi protein-RHOA axis by non-canonical Hedgehog signaling is independent of primary cilia. *PLoS One* 13:e0203170. 10.1371/journal.pone.0203170 30148884 PMC6110505

[B94] Hollinger S. Hepler J. R. (2002). Cellular regulation of RGS proteins: modulators and integrators of G protein signaling. *Pharmacol. Rev.* 54 527–559. 10.1124/pr.54.3.527 12223533

[B95] Hollmann M. W. Strumper D. Herroeder S. Durieux M. E. (2005). Receptors, G proteins, and their interactions. *Anesthesiology* 103 1066–1078. 10.1097/00000542-200511000-00022 16249682

[B96] Hovelsø N. Sotty F. Montezinho L. P. Pinheiro P. S. Herrik K. F. Mørk A. (2012). Therapeutic potential of metabotropic glutamate receptor modulators. *Curr. Neuropharmacol.* 10 12–48. 10.2174/157015912799362805 22942876 PMC3286844

[B97] Huang Y. Todd N. Thathiah A. (2017). The role of GPCRs in neurodegenerative diseases: avenues for therapeutic intervention. *Curr. Opin. Pharmacol.* 32 96–110. 10.1016/j.coph.2017.02.001 28288370

[B98] Huang Z.-L. Zhang Z. Qu W.-M. (2014). Roles of adenosine and its receptors in sleep-wake regulation. *Int. Rev. Neurobiol.* 119 349–371. 10.1016/B978-0-12-801022-8.00014-3 25175972

[B99] Huguet F. Drieu K. Piriou A. (1994). Decreased cerebral 5-HT1A receptors during ageing: reversal by Ginkgo biloba extract (EGb 761). *J. Pharm. Pharmacol.* 46 316–318. 10.1111/j.2042-7158.1994.tb03802.x 8051617

[B100] Hunyady L. Catt K. J. (2006). Pleiotropic AT1 receptor signaling pathways mediating physiological and pathogenic actions of angiotensin II. *Mol. Endocrinol.* 20 953–970. 10.1210/me.2004-0536 16141358

[B101] Hurley M. J. Mash D. C. Jenner P. (2003). Expression of cannabinoid CB 1 receptor mRNA in basal ganglia of normal and parkinsonian human brain. *J. Neural Transm.* 110 1279–1288. 10.1007/s00702-003-0033-7 14628192

[B102] Ihnatovych I. Novotny J. Haugvicova R. Bourova L. Mares P. Svoboda P. (2002). Opposing changes of trimeric G protein levels during ontogenetic development of rat brain. *Brain Res. Dev. Brain Res.* 133 57–67. 10.1016/S0165-3806(01)00322-4 11850064

[B103] Inoue M. Suhara T. Sudo Y. Okubo Y. Yasuno F. Kishimoto T. (2001). Age-related reduction of extrastriatal dopamine D2 receptor measured by PET. *Life Sci.* 69 1079–1084. 10.1016/S0024-3205(01)01205-X 11508650

[B104] Ishibashi K. Ishii K. Oda K. Kawasaki K. Mizusawa H. Ishiwata K. (2009). Regional analysis of age-related decline in dopamine transporters and dopamine D2-like receptors in human striatum. *Synapse* 63 282–290. 10.1002/syn.20603 19116949

[B105] Jackson L. Eldahshan W. Fagan S. C. Ergul A. (2018). Within the brain: the renin angiotensin system. *Int. J. Mol. Sci.* 19:E876. 10.3390/ijms19030876 29543776 PMC5877737

[B106] Jiang M. Bajpayee N. S. (2009). Molecular mechanisms of go signaling. *Neurosignals* 17 23–41. 10.1159/000186688 19212138 PMC2836949

[B107] Jiang M. Spicher K. Boulay G. Martín-Requero A. Dye C. A. Rudolph U. (2002). Mouse gene knockout and knockin strategies in application to alpha subunits of Gi/Go family of G proteins. *Methods Enzymol.* 344 277–298. 10.1016/S0076-6879(02)44721-0 11771389

[B108] Jiang M. Spicher K. Boulay G. Wang Y. Birnbaumer L. (2001). Most central nervous system D2 dopamine receptors are coupled to their effectors by Go. *Proc. Natl. Acad. Sci.U.S.A.* 98 3577–3582. 10.1073/pnas.051632598 11248120 PMC30695

[B109] Jo S. Yarishkin O. Hwang Y. J. Chun Y. E. Park M. Woo D. H. (2014). GABA from reactive astrocytes impairs memory in mouse models of Alzheimer’s disease. *Nat. Med.* 20 886–896. 10.1038/nm.3639 24973918 PMC8385452

[B110] Joyce J. N. Loeschen S. K. Sapp D. W. Marshall J. F. (1986). Age-related regional loss of caudate-putamen dopamine receptors revealed by quantitative autoradiography. *Brain Res.* 378 158–163. 10.1016/0006-8993(86)90298-2 2943360

[B111] Kaasinen V. Vilkman H. Hietala J. Någren K. Helenius H. Olsson H. (2000). Age-related dopamine D2/D3 receptor loss in extrastriatal regions of the human brain. *Neurobiol. Aging* 21 683–688. 10.1016/S0197-4580(00)00149-4 11016537

[B112] Kamakura S. Nomura M. Hayase J. Iwakiri Y. Nishikimi A. Takayanagi R. (2013). The cell polarity protein minsc regulates neutrophil chemotaxis via a noncanonical G protein signaling pathway. *Dev. Cell* 26 292–302. 10.1016/j.devcel.2013.06.008 23891662

[B113] Karrer T. M. Josef A. K. Mata R. Morris E. D. Samanez-Larkin G. R. (2017). Reduced dopamine receptors and transporters but not synthesis capacity in normal aging adults: a meta-analysis. *Neurobiol. Aging* 57 36–46. 10.1016/j.neurobiolaging.2017.05.006 28599217 PMC5645072

[B114] Katada T. Oinuma M. Ui M. (1986). Two guanine nucleotide-binding proteins in rat brain serving as the specific substrate of islet-activating protein, pertussis toxin. Interaction of the alpha-subunits with beta gamma-subunits in development of their biological activities. *J. Biol. Chem.* 261 8182–8191. 3087970

[B115] Kilander M. B. C. Dahlström J. Schulte G. (2014a). Assessment of Frizzled 6 membrane mobility by FRAP supports G protein coupling and reveals WNT-Frizzled selectivity. *Cell. Signal.* 26 1943–1949. 10.1016/j.cellsig.2014.05.012 24873871

[B116] Kilander M. B. C. Petersen J. Andressen K. W. Ganji R. S. Levy F. O. Schuster J. (2014b). Disheveled regulates precoupling of heterotrimeric G proteins to Frizzled 6. *FASEB J.* 28 2293–2305. 10.1096/fj.13-246363 24500924

[B117] Ko J. Choii G. Um J. W. (2015). The balancing act of GABAergic synapse organizers. *Trends Mol. Med.* 21 256–268. 10.1016/j.molmed.2015.01.004 25824541

[B118] Kobayashi I. Shibasaki H. Takahashi K. Tohyama K. Kurachi Y. Ito H. (1990). Purification and characterization of five different alpha subunits of guanine-nucleotide-binding proteins in bovine brain membranes. Their physiological properties concerning the activities of adenylate cyclase and atrial muscarinic K+ channels. *Eur. J. Biochem.* 191 499–506. 10.1111/j.1432-1033.1990.tb19149.x 2116967

[B119] Kolakowski L. F. (1994). GCRDb: a G-protein-coupled receptor database. *Recept. Channels* 2 1–7. 10.4236/ojgen.2012.24B003 8081729

[B120] Kristiansen K. (2004). Molecular mechanisms of ligand binding, signaling, and regulation within the superfamily of G-protein-coupled receptors: molecular modeling and mutagenesis approaches to receptor structure and function. *Pharmacol. Ther.* 103 21–80. 10.1016/j.pharmthera.2004.05.002 15251227

[B121] Kroeze W. K. Sheffler D. J. Roth B. L. (2003). G-protein-coupled receptors at a glance. *J. Cell Sci.* 116 4867–4869. 10.1242/jcs.00902 14625380

[B122] Kumar U. (2005). Expression of somatostatin receptor subtypes (SSTR1-5) in Alzheimer’s disease brain: an immunohistochemical analysis. *Neuroscience* 134 525–538. 10.1016/j.neuroscience.2005.04.001 15961235

[B123] Lagerström M. C. Schiöth H. B. (2008). Structural diversity of G protein-coupled receptors and significance for drug discovery. *Nat. Rev. Drug Discov.* 7 339–357. 10.1038/nrd2518 18382464

[B124] Lai H. Bowden D. M. Horita A. (1987). Age-related decreases in dopamine receptors in the caudate nucleus and putamen of the rhesus monkey (*Macaca mulatta*). *Neurobiol. Aging* 8 45–49. 10.1016/0197-4580(87)90056-X 3561665

[B125] Laquerriere A. Leroux P. Bodenant C. Gonzalez B. Tayot J. Vaudry H. (1994). Quantitative autoradiographic study of somatostatin receptors in the adult human cerebellum. *Neuroscience* 62 1147–1154. 10.1016/0306-4522(94)90350-6 7845591

[B126] Laschet C. Dupuis N. Hanson J. (2018). The G protein-coupled receptors deorphanization landscape. *Biochem. Pharmacol.* 153 62–74. 10.1016/j.bcp.2018.02.016 29454621

[B127] Le Verche V. Kaindl A. M. Verney C. Csaba Z. Peineau S. Olivier P. (2009). The somatostatin 2A receptor is enriched in migrating neurons during rat and human brain development and stimulates migration and axonal outgrowth. *PLoS One* 4:e5509. 10.1371/journal.pone.0005509 19434240 PMC2677669

[B128] Lebois E. P. Thorn C. Edgerton J. R. Popiolek M. Xi S. (2018). Muscarinic receptor subtype distribution in the central nervous system and relevance to aging and Alzheimer’s disease. *Neuropharmacology* 136 362–373. 10.1016/j.neuropharm.2017.11.018 29138080

[B129] Lee H. J. Clagett-Dame M. Heideman W. Weiler M. S. (1994). The effect of age on muscarinic receptor transcripts in rat brain. *Neurosci. Lett.* 174 205–208. 10.1016/0304-3940(94)90022-1 7970180

[B130] Lerer B. Gelfin Y. Shapira B. (1999). Neuroendocrine evidence for age-related decline in central serotonergic function. *Neuropsychopharmacology* 21 321–322. 10.1016/S0893-133X(98)00125-0 10432480

[B131] Leroux P. Bodenant C. Bologna E. Gonzalez B. Vaudry H. (1995). Transient expression of somatostatin receptors in the brain during development. *Ciba Found. Symp.* 190 127–37; discussion 137–41. 7587643 10.1002/9780470514733.ch8

[B132] Leung C. Wong Y. (2017). Role of G protein-coupled receptors in the regulation of structural plasticity and cognitive function. *Molecules* 22:1239. 10.3390/molecules22071239 28737723 PMC6152405

[B133] Li X. Greenwood A. F. Powers R. Jope R. S. (1996). Effects of postmortem interval, age, and Alzheimer’s disease on G-proteins in human brain. *Neurobiol. Aging* 17 115–122. 10.1016/0197-4580(95)02023-3 8786793

[B134] Li Y. Sun H. Chen Z. Xu H. Bu G. Zheng H. (2016). Implications of GABAergic neurotransmission in Alzheimer’s disease. *Front. Aging Neurosci.* 8:31 10.3389/fnagi.2016.00031 PMC4763334 26941642

[B135] Liao C. Han Q. Ma Y. Su B. (2016). Age-related gene expression change of GABAergic system in visual cortex of rhesus macaque. *Gene* 590 227–233. 10.1016/j.gene.2016.05.010 27196061

[B136] Lidow M. S. Goldman-Rakic P. S. Rakic P. Gallager D. W. (1988). Differential quenching and limits of resolution in autoradiograms of brain tissue labeled with 3H-, 125I- and 14C-compounds. *Brain Res.* 459 105–119. 10.1016/0006-8993(88)90290-9 3167570

[B137] Lin C. Koval A. Tishchenko S. Gabdulkhakov A. Tin U. Solis G. P. (2014). Double suppression of the Gα protein activity by RGS proteins. *Mol. Cell* 53 663–671. 10.1016/j.molcel.2014.01.014 24560274

[B138] Liu J. J. Sharma K. Zangrandi L. Chen C. Humphrey S. J. Chiu Y. T. (2018). In vivo brain GPCR signaling elucidated by phosphoproteomics. *Science* 360:eaao4927. 10.1126/science.aao4927 29930108 PMC6527112

[B139] Loerch P. M. Lu T. Dakin K. A. Vann J. M. Isaacs A. Geula C. (2008). Evolution of the aging brain transcriptome and synaptic regulation. *PLoS One* 3:e3329. 10.1371/journal.pone.0003329 18830410 PMC2553198

[B140] Lütjens R. Rocher J.-P. (2017). Recent advances in drug discovery of GPCR allosteric modulators for neurodegenerative disorders. *Curr. Opin. Pharmacol.* 32 91–95. 10.1016/j.coph.2017.01.001 28135635

[B141] Ma’ayan A. Jenkins S. L. Barash A. Iyengar R. (2009). Neuro2A differentiation by Galphai/o pathway. *Sci. Signal.* 2 cm1. 10.1126/scisignal.254cm1 19155528 PMC2649824

[B142] Maccarrone M. Attinà M. Bari M. Cartoni A. Ledent C. Finazzi-Agrò A. (2001). Anandamide degradation and N-acylethanolamines level in wild-type and CB1 cannabinoid receptor knockout mice of different ages. *J. Neurochem.* 78 339–348. 10.1046/j.1471-4159.2001.00413.x 11461969

[B143] Maggi R. Limonta P. Dondi D. Martini L. Piva F. (1989). Distribution of kappa opioid receptors in the brain of young and old male rats. *Life Sci.* 45 2085–2092. 10.1016/0024-3205(89)90073-8 2557515

[B144] Mailleux P. Vanderhaeghen J. J. (1992). Age-related loss of cannabinoid receptor binding sites and mRNA in the rat striatum. *Neurosci. Lett.* 147 179–181. 10.1016/0304-3940(92)90589-Y 1491804

[B145] Mairesse J. Gatta E. Reynaert M.-L. Marrocco J. Morley-Fletcher S. Soichot M. (2015). Activation of presynaptic oxytocin receptors enhances glutamate release in the ventral hippocampus of prenatally restraint stressed rats. *Psychoneuroendocrinology* 62 36–46. 10.1016/j.psyneuen.2015.07.005 26231445

[B146] Maroteaux L. Béchade C. Roumier A. (2019). Dimers of serotonin receptors: impact on ligand affinity and signaling. *Biochimie* 10.1016/j.biochi.2019.01.009 [Epub ahead of print]. 30685449

[B147] Masuho I. Ostrovskaya O. Kramer G. M. Jones C. D. Xie K. Martemyanov K. A. (2015). Distinct profiles of functional discrimination among G proteins determine the actions of G protein-coupled receptors. *Sci. Signal.* 8:ra123. 10.1126/scisignal.aab4068 26628681 PMC4886239

[B148] Mathieu-Kia A. M. Fan L. Q. Kreek M. J. Simon E. J. Hiller J. M. (2001). Mu-, delta- and kappa-opioid receptor populations are differentially altered in distinct areas of postmortem brains of Alzheimer’s disease patients. *Brain Res.* 893 121–134. 10.1016/S0006-8993(00)03302-3 11223000

[B149] Mato S. Pazos A. (2004). Influence of age, postmortem delay and freezing storage period on cannabinoid receptor density and functionality in human brain. *Neuropharmacology* 46 716–726. 10.1016/j.neuropharm.2003.11.004 14996549

[B150] Matuskey D. Pittman B. Planeta-Wilson B. Walderhaug E. Henry S. Gallezot J.-D. (2012). Age effects on serotonin receptor 1B as assessed by PET. *J. Nucl. Med.* 53 1411–1414. 10.2967/jnumed.112.103598 22851636 PMC3690814

[B151] Matuskey D. Worhunksy P. Correa E. Pittman B. Gallezot J.-D. Nabulsi N. (2016). Age-related changes in binding of the D2/3 receptor radioligand [(11)C](+)PHNO in healthy volunteers. *Neuroimage* 130 241–247. 10.1016/j.neuroimage.2016.02.002 26876475 PMC4808424

[B152] McCutcheon J. E. Marinelli M. (2009). Age matters. *Eur. J. Neurosci.* 29 997–1014. 10.1111/j.1460-9568.2009.06648.x 19291226 PMC2761206

[B153] McLaughlin M. Ross B. M. Milligan G. McCulloch J. Knowler J. T. (1991). Robustness of G proteins in Alzheimer’s disease: an immunoblot study. *J. Neurochem.* 57 9–14. 10.1111/j.1471-4159.1991.tb02092.x 1904911

[B154] McQuail J. A. Bañuelos C. LaSarge C. L. Nicolle M. M. Bizon J. L. (2012). GABAB receptor GTP-binding is decreased in the prefrontal cortex but not the hippocampus of aged rats. *Neurobiol. Aging* 33 1124.e1–1124.e12. 10.1016/j.neurobiolaging.2011.11.011 22169202 PMC3310948

[B155] McQuail J. A. Davis K. N. Miller F. Hampson R. E. Deadwyler S. A. Howlett A. C. (2013). Hippocampal Gαq/11 but not Gαo-coupled receptors are altered in aging. *Neuropharmacology* 70 63–73. 10.1016/j.neuropharm.2013.01.009 23347951 PMC3705722

[B156] Meana J. J. Barturen F. Garro M. A. García-Sevilla J. A. Fontán A. Zarranz J. J. (1992). Decreased density of presynaptic alpha 2-adrenoceptors in postmortem brains of patients with Alzheimer’s disease. *J. Neurochem.* 58 1896–1904. 10.1111/j.1471-4159.1992.tb10067.x 1373179

[B157] Meerlo P. Roman V. Farkas E. Keijser J. N. Nyakas C. Luiten P. G. M. (2004). Ageing-related decline in adenosine A1 receptor binding in the rat brain: an autoradiographic study. *J. Neurosci. Res.* 78 742–748. 10.1002/jnr.20314 15470722

[B158] Meltzer C. C. Drevets W. C. Price J. C. Mathis C. A. Lopresti B. Greer P. J. (2001). Gender-specific aging effects on the serotonin 1A receptor. *Brain Res.* 895 9–17. 10.1016/S0006-8993(00)03211-X 11259754

[B159] Meltzer C. C. Smith G. DeKosky S. T. Pollock B. G. Mathis C. A. Moore R. Y. (1998). Serotonin in aging, late-life depression, and Alzheimer’s disease: the emerging role of functional imaging. *Neuropsychopharmacology* 18 407–430. 10.1016/S0893-133X(97)00194-2 9571651

[B160] Meng S. Z. Ozawa Y. Itoh M. Takashima S. (1999). Developmental and age-related changes of dopamine transporter, and dopamine D1 and D2 receptors in human basal ganglia. *Brain Res.* 843 136–144. 10.1016/S0006-8993(99)01933-2 10528120

[B161] Mesco E. R. Joseph J. A. Blake M. J. Roth G. S. (1991). Loss of D2 receptors during aging is partially due to decreased levels of mRNA. *Brain Res.* 545 355–357. 10.1016/0006-8993(91)91314-Q 1830509

[B162] Messing R. B. Vasquez B. J. Samaniego B. Jensen R. A. Martinez J. L. McGaugh J. L. (1981). Alterations in dihydromorphine binding in cerebral hemispheres of aged male rats. *J. Neurochem.* 36 784–787. 10.1111/j.1471-4159.1981.tb01659.x 6257860

[B163] Meyer P. T. Elmenhorst D. Boy C. Winz O. Matusch A. Zilles K. (2007). Effect of aging on cerebral A1 adenosine receptors: a [18F]CPFPX PET study in humans. *Neurobiol. Aging* 28 1914–1924. 10.1016/j.neurobiolaging.2006.08.005 16996650

[B164] Milbrandt J. C. Albin R. L. Caspary D. M. (1994). Age-related decrease in GABAB receptor binding in the fischer 344 rat inferior colliculus. *Neurobiol. Aging* 15 699–703. 10.1016/0197-4580(94)90051-5 7891824

[B165] Milligan G. Kostenis E. (2006). Heterotrimeric G-proteins: a short history. *Br. J. Pharmacol.* 147(Suppl.), S46–S55. 10.1038/sj.bjp.0706405 16402120 PMC1760735

[B166] Mizukami K. Ishikawa M. Akatsu H. Abrahamson E. E. Ikonomovic M. D. Asada T. (2011). An immunohistochemical study of the serotonin 1A receptor in the hippocampus of subjects with Alzheimer’s disease. *Neuropathology* 31 503–509. 10.1111/j.1440-1789.2010.01193.x 21269332 PMC3112246

[B167] Mombaerts P. (2004). Genes and ligands for odorant, vomeronasal and taste receptors. *Nat. Rev. Neurosci.* 5 263–278. 10.1038/nrn1365 15034552

[B168] Moore T. L. Schettler S. P. Killiany R. J. Herndon J. G. Luebke J. I. Moss M. B. (2005). Cognitive impairment in aged rhesus monkeys associated with monoamine receptors in the prefrontal cortex. *Behav. Brain Res.* 160 208–221. 10.1016/j.bbr.2004.12.003 15863218

[B169] Morgan D. G. Marcusson J. O. Nyberg P. Wester P. Winblad B. Gordon M. N. (1987). Divergent changes in D-1 and D-2 dopamine binding sites in human brain during aging. *Neurobiol. Aging* 8 195–201. 10.1016/0197-4580(87)90002-9 3600950

[B170] Morris E. D. Chefer S. I. Lane M. A. Muzic R. F. Wong D. F. Dannals R. F. (1999). Loss of D2 receptor binding with age in rhesus monkeys: importance of correction for differences in striatal size. *J. Cereb. Blood Flow Metab.* 19 218–229. 10.1097/00004647-199902000-00013 10027777

[B171] Munk C. Isberg V. Mordalski S. Harpsøe K. Rataj K. Hauser A. S. (2016). GPCRdb: the G protein-coupled receptor database - an introduction. *Br. J. Pharmacol.* 173 2195–2207. 10.1111/bph.13509 27155948 PMC4919580

[B172] Nakajima S. Caravaggio F. Boileau I. Chung J. K. Plitman E. Gerretsen P. (2015). Lack of age-dependent decrease in dopamine D3 receptor availability: a [(11)C]-(+)-PHNO and [(11)C]-raclopride positron emission tomography study. *J. Cereb. Blood Flow Metab.* 35 1812–1818. 10.1038/jcbfm.2015.129 26058690 PMC4635236

[B173] Naoi M. Maruyama W. (1999). Cell death of dopamine neurons in aging and Parkinson’s disease. *Mech. Ageing Dev.* 111 175–188. 10.1016/S0047-6374(99)00064-0 10656535

[B174] Nautiyal K. M. Tanaka K. F. Barr M. M. Tritschler L. Le Dantec Y. David D. J. (2015). Distinct circuits underlie the effects of 5-HT1B receptors on aggression and impulsivity. *Neuron* 86 813–826. 10.1016/j.neuron.2015.03.041 25892302 PMC4431594

[B175] Navarro G. Cordomí A. Casadó-Anguera V. Moreno E. Cai N. S. Cortés A. (2018). Evidence for functional pre-coupled complexes of receptor heteromers and adenylyl cyclase. *Nat. Commun.* 9 1–12. 10.1038/s41467-018-03522-3 29593213 PMC5871782

[B176] Nehmé R. Carpenter B. Singhal A. Strege A. Edwards P. C. White C. F. (2017). Mini-G proteins: novel tools for studying GPCRs in their active conformation. *PLoS One* 12:e0175642. 10.1371/journal.pone.0175642 28426733 PMC5398546

[B177] Nieves-Martinez E. Haynes K. Childers S. R. Sonntag W. E. Nicolle M. M. (2012). Muscarinic receptor/G-protein coupling is reduced in the dorsomedial striatum of cognitively impaired aged rats. *Behav. Brain Res.* 227 258–264. 10.1016/j.bbr.2011.10.048 22085876 PMC3253526

[B178] Nishimura A. Sunggip C. Oda S. Numaga-Tomita T. Tsuda M. Nishida M. (2017). Purinergic P2Y receptors: molecular diversity and implications for treatment of cardiovascular diseases. *Pharmacol. Ther.* 180 113–128. 10.1016/j.pharmthera.2017.06.010 28648830

[B179] Nobles M. Benians A. Tinker A. (2005). Heterotrimeric G proteins precouple with G protein-coupled receptors in living cells. *Proc. Natl. Acad. Sci. U.S.A.* 102 18706–18711. 10.1073/pnas.0504778102 16352729 PMC1317907

[B180] Nomura Y. Kitamura Y. Kawai M. Segawa T. (1986). Alpha 2-adrenoceptor-GTP binding regulatory protein-adenylate cyclase system in cerebral cortical membranes of adult and senescent rats. *Brain Res.* 379 118–124. 10.1016/0006-8993(86)90263-5 3017507

[B181] Nordberg A. Alafuzoff I. Winblad B. (1992). Nicotinic and muscarinic subtypes in the human brain: changes with aging and dementia. *J. Neurosci. Res.* 31 103–111. 10.1002/jnr.490310115 1613816

[B182] O’Boyle K. M. Waddington J. L. (1984). Loss of rat striatal dopamine receptors with ageing is selective for D-2 but not D-1 sites: association with increased non-specific binding of the D-1 ligand [3H]piflutixol. *Eur. J. Pharmacol.* 105 171–174. 10.1016/0014-2999(84)90663-0 6149143

[B183] Offermanns S. (2003). G-proteins as transducers in transmembrane signalling. *Prog. Biophys. Mol. Biol.* 83 101–130. 10.1016/S0079-6107(03)00052-X 12865075

[B184] Offermanns S. Rosenthal W. (2008). *Encyclopedia of Molecular Pharmacology.* Berlin: Springer 10.1007/978-3-540-38918-7

[B185] Okamoto T. Takeda S. Murayama Y. Ogata E. Nishimoto I. (1995). Ligand-dependent G protein coupling function of amyloid transmembrane precursor. *J. Biol. Chem.* 270 4205–4208. 10.1074/jbc.270.9.4205 7876177

[B186] O’Neill C. Wiehager B. Fowler C. J. Ravid R. Winblad B. Cowburn R. F. (1994). Regionally selective alterations in G protein subunit levels in the Alzheimer’s disease brain. *Brain Res.* 636 193–201. 10.1016/0006-8993(94)91017-0 8012802

[B187] Pagonopoulou O. Angelatou F. (1992). Reduction of A1 adenosine receptors in cortex, hippocampus and cerebellum in ageing mouse brain. *Neuroreport* 3 735–737. 10.1097/00001756-199209000-00003 1421127

[B188] Palczewski K. Kumasaka T. Hori T. Behnke C. A. Motoshima H. Fox B. A. (2000). Crystal structure of rhodopsin: a G protein-coupled receptor. *Science* 289 739–745. 10.1126/science.289.5480.739 10926528

[B189] Palego L. Marazziti D. Rossi A. Giannaccini G. Naccarato A. G. Lucacchini A. (1997). Apparent absence of aging and gender effects on serotonin 1A receptors in human neocortex and hippocampus. *Brain Res.* 758 26–32. 10.1016/S0006-8993(96)01415-1 9203529

[B190] Pandya M. Palpagama T. H. Turner C. Waldvogel H. J. Faull R. L. Kwakowsky A. (2019). Sex- and age-related changes in GABA signaling components in the human cortex. *Biol. Sex Differ.* 10:5. 10.1186/s13293-018-0214-6 30642393 PMC6332906

[B191] Park D. O’Doherty I. Somvanshi R. K. Bethke A. Schroeder F. C. Kumar U. (2012). Interaction of structure-specific and promiscuous G-protein-coupled receptors mediates small-molecule signaling in *Caenorhabditis elegans*. *Proc. Natl. Acad. Sci. U.S.A.* 109 9917–9922. 10.1073/pnas.1202216109 22665789 PMC3382479

[B192] Parker M. S. Park E. A. Sallee F. R. Parker S. L. (2011). Two intracellular helices of G-protein coupling receptors could generally support oligomerization and coupling with transducers. *Amino Acids* 40 261–268. 10.1007/s00726-010-0616-1 20571842

[B193] Parsey R. V. Oquendo M. A. Simpson N. R. Ogden R. T. Van Heertum R. Arango V. (2002). Effects of sex, age, and aggressive traits in man on brain serotonin 5-HT1A receptor binding potential measured by PET using [C-11]WAY-100635. *Brain Res.* 954 173–182. 10.1016/S0006-8993(02)03243-2 12414100

[B194] Pascual J. del Arco C. González A. M. Díaz A. del Olmo E. Pazos A. (1991). Regionally specific age-dependent decline in alpha 2-adrenoceptors: an autoradiographic study in human brain. *Neurosci. Lett.* 133 279–283. 10.1016/0304-3940(91)90588-K 1687760

[B195] Patel Y. C. (1999). Somatostatin and its receptor family. *Front. Neuroendocrinol.* 20:157–198. 10.1006/frne.1999.0183 10433861

[B196] Peres C. M. Aronoff D. M. Serezani C. H. Flamand N. Faccioli L. H. Peters-Golden M. (2007). Specific leukotriene receptors couple to distinct G proteins to effect stimulation of alveolar macrophage host defense functions. *J. Immunol.* 179 5454–5461. 10.4049/jimmunol.179.8.5454 17911632

[B197] Petroff O. A. C. (2002). GABA and glutamate in the human brain. *Neuroscientist* 8 562–573. 10.1177/1073858402238515 12467378

[B198] Petryszak R. Keays M. Tang Y. A. Fonseca N. A. Barrera E. Burdett T. (2016). Expression Atlas update–an integrated database of gene and protein expression in humans, animals and plants. *Nucleic Acids Res.* 44 D746–D752. 10.1093/nar/gkv1045 26481351 PMC4702781

[B199] Piggott M. A. Owens J. O’Brien J. Colloby S. Fenwick J. Wyper D. (2003). Muscarinic receptors in basal ganglia in dementia with Lewy bodies, Parkinson’s disease and Alzheimer’s disease. *J. Chem. Neuroanat.* 25 161–173. 10.1016/S0891-0618(03)00002-4 12706204

[B200] Piva F. Maggi R. Limonta P. Dondi D. Martini L. (1987). Decrease of mu opioid receptors in the brain and in the hypothalamus of the aged male rat. *Life Sci.* 40 391–398. 10.1016/0024-3205(87)90141-X 3027483

[B201] Piyanova A. Albayram O. Rossi C. A. Farwanah H. Michel K. Nicotera P. (2013). Loss of CB1 receptors leads to decreased cathepsin D levels and accelerated lipofuscin accumulation in the hippocampus. *Mech. Ageing Dev.* 134 391–399. 10.1016/j.mad.2013.08.001 23954857

[B202] Pohjalainen T. Rinne J. O. Någren K. Syvälahti E. Hietala J. (1998). Sex differences in the striatal dopamine D2 receptor binding characteristics in vivo. *Am. J. Psychiatry* 155 768–773. 10.1176/ajp.155.6.768 9619148

[B203] Polizio A. H. Chinchilla P. Chen X. Kim S. Manning D. R. Riobo N. A. (2011a). Heterotrimeric Gi proteins link Hedgehog signaling to activation of Rho small GTPases to promote fibroblast migration. *J. Biol. Chem.* 286 19589–19596. 10.1074/jbc.M110.197111 21474452 PMC3103338

[B204] Polizio A. H. Chinchilla P. Chen X. Manning D. R. Riobo N. A. (2011b). Sonic Hedgehog activates the GTPases Rac1 and RhoA in a Gli-independent manner through coupling of smoothened to Gi proteins. *Sci. Signal.* 4:t7. 10.1126/scisignal.2002396 22114142 PMC5811764

[B205] Prieto G. A. (2017). Abnormalities of dopamine D3 receptor signaling in the diseased brain. *J. Cent. Nerv. Syst. Dis.* 9:1179573517726335. 10.1177/1179573517726335 28855798 PMC5562332

[B206] Rangel-Barajas C. Coronel I. Florán B. (2015). Dopamine receptors and neurodegeneration. *Aging Dis.* 6 349–368. 10.14336/AD.2015.0330 26425390 PMC4567218

[B207] Rask-Andersen M. Masuram S. Schiöth H. B. (2014). The druggable genome: evaluation of drug targets in clinical trials suggests major shifts in molecular class and indication. *Annu. Rev. Pharmacol. Toxicol.* 54 9–26. 10.1146/annurev-pharmtox-011613-135943 24016212

[B208] Raz N. Rodrigue K. M. Kennedy K. M. Head D. Gunning-Dixon F. Acker J. D. (2003). Differential aging of the human striatum: longitudinal evidence. *AJNR Am. J. Neuroradiol.* 24 1849–1856. 14561615 PMC7976312

[B209] Reed D. K. Korytko A. I. Hipkin R. W. Wehrenberg W. B. Schonbrunn A. Cuttler L. (1999). Pituitary somatostatin receptor (sst)1-5 expression during rat development: age-dependent expression of sst2. *Endocrinology* 140 4739–4744. 10.1210/endo.140.10.7033 10499533

[B210] Resende M. Vieira S. I. (2012). “Proteínas G na Transdução de Sinal,” in *O eSsencial em … Sinalizaç*ão Celular, eds Fardilha M. da Cruz e Silva O. A. B. Conde M. (Aveiro: Edições Afrontamento/Departamento de Biologia da Universidade de Aveiro), 94–129.

[B211] Ribeiro F. M. Vieira L. B. Pires R. G. W. Olmo R. P. Ferguson S. S. G. (2017). Metabotropic glutamate receptors and neurodegenerative diseases. *Pharmacol. Res.* 115 179–191. 10.1016/j.phrs.2016.11.013 27872019

[B212] Ricci A. Mammola C. L. Vega J. A. Zaccheo D. Amenta F. (1996). Density and pattern of dopamine D2-like receptors in the cerebellar cortex of aged rats. *Neurobiol. Aging* 17 45–52. 10.1016/0197-4580(95)02029-2 8786802

[B213] Rinne J. O. (1987). Muscarinic and dopaminergic receptors in the aging human brain. *Brain Res.* 404 162–168. 10.1016/0006-8993(87)91367-9 3567563

[B214] Rinne J. O. Hietala J. Ruotsalainen U. Säkö E. Laihinen A. Någren K. (1993). Decrease in human striatal dopamine D2 receptor density with age: a PET study with [11C]raclopride. *J. Cereb. Blood Flow Metab.* 13 310–314. 10.1038/jcbfm.1993.39 8436624

[B215] Rinne J. O. Lönnberg P. Marjamäki P. (1990). Age-dependent decline in human brain dopamine D1 and D2 receptors. *Brain Res.* 508 349–352. 10.1016/0006-8993(90)90423-9 2407314

[B216] Rodefer J. S. Nguyen T. N. (2008). Naltrexone reverses age-induced cognitive deficits in rats. *Neurobiol. Aging* 29 309–313. 10.1016/j.neurobiolaging.2006.10.005 17098330

[B217] Romero J. Berrendero F. Garcia-Gil L. de la Cruz P. Ramos J. A. Fernández-Ruiz J. J. (1998). Loss of cannabinoid receptor binding and messenger RNA levels and cannabinoid agonist-stimulated [35S]guanylyl-5′O-(thio)-triphosphate binding in the basal ganglia of aged rats. *Neuroscience* 84 1075–1083. 10.1016/S0306-4522(97)00552-6 9578396

[B218] Sabelhaus C. F. Schröder U. H. Breder J. Henrich-Noack P. Reymann K. G. (2000). Neuroprotection against hypoxic/hypoglycaemic injury after the insult by the group III metabotropic glutamate receptor agonist (R,S)-4-phosphonophenylglycine. *Br. J. Pharmacol.* 131 655–658. 10.1038/sj.bjp.0703646 11030711 PMC1572399

[B219] Sarajärvi T. Marttinen M. Natunen T. Kauppinen T. Mäkinen P. Helisalmi S. (2015). Genetic variation in δ-opioid receptor associates with increased β- and γ-secretase activity in the late stages of Alzheimer’s disease. *J. Alzheimers. Dis.* 48 507–516. 10.3233/JAD-150221 26402014

[B220] Scarr E. (2012). Muscarinic receptors: their roles in disorders of the central nervous system and potential as therapeutic targets. *CNS Neurosci. Ther.* 18 369–379. 10.1111/j.1755-5949.2011.00249.x 22070219 PMC6493542

[B221] Schoenbaum G. Nugent S. Saddoris M. P. Gallagher M. (2002). Teaching old rats new tricks: age-related impairments in olfactory reversal learning. *Neurobiol. Aging* 23 555–564. 10.1016/S0197-4580(01)00343-8 12009505

[B222] Seeman P. Bzowej N. H. Guan H. C. Bergeron C. Becker L. E. Reynolds G. P. (1987). Human brain dopamine receptors in children and aging adults. *Synapse* 1 399–404. 10.1002/syn.890010503 3505371

[B223] Sengupta P. (2013). The laboratory rat: relating its age with human’s. *Int. J. Prev. Med.* 4 624–630. 23930179 PMC3733029

[B224] Shaked G. M. Chauv S. Ubhi K. Hansen L. A. Masliah E. (2009). Interactions between the amyloid precursor protein C-terminal domain and G proteins mediate calcium dysregulation and amyloid beta toxicity in Alzheimer’s disease. *FEBS J.* 276 2736–2751. 10.1111/j.1742-4658.2009.06997.x 19368557 PMC2838422

[B225] Sharp S. I. Ballard C. G. Ziabreva I. Piggott M. A. Perry R. H. Perry E. K. (2008). Cortical serotonin 1A receptor levels are associated with depression in patients with dementia with lewy bodies and Parkinson’s disease dementia. *Dement. Geriatr. Cogn. Disord.* 26 330–338. 10.1159/000161058 18841018

[B226] Shimokawa I. Yanagihara K. Higami Y. Okimoto T. Tomita M. Ikeda T. (2000). Effects of aging and dietary restriction on mRNA levels of receptors for growth hormone-releasing hormone and somatostatin in the rat pituitary. *J. Gerontol. A Biol. Sci. Med. Sci.* 55 B274–B279. 10.1093/gerona/55.6.B274 10843343

[B227] Silbereis J. C. Pochareddy S. Zhu Y. Li M. Sestan N. (2016). The cellular and molecular landscapes of the developing human central nervous system. *Neuron* 89 248–268. 10.1016/j.neuron.2015.12.008 26796689 PMC4959909

[B228] Simonyi A. Miller L. A. Sun G. Y. (2000). Region-specific decline in the expression of metabotropic glutamate receptor 7 mRNA in rat brain during aging. *Brain Res. Mol. Brain Res.* 82 101–106. 10.1016/S0169-328X(00)00189-3 11042362

[B229] Simonyi A. Ngomba R. T. Storto M. Catania M. V. Miller L. A. Youngs B. (2005). Expression of groups I and II metabotropic glutamate receptors in the rat brain during aging. *Brain Res.* 1043 95–106. 10.1016/j.brainres.2005.02.046 15862522

[B230] Sirohi S. Walker B. M. (2015). Maturational alterations in constitutive activity of medial prefrontal cortex kappa-opioid receptors in Wistar rats. *J. Neurochem.* 135 659–665. 10.1111/jnc.13279 26257334 PMC4636924

[B231] Sirvio J. Jolkkonen J. Pitkanen A. Riekkinen P. J. (1987). Age dependence of somatostatin levels and somatostatin binding in the rat brain. *Comp. Biochem. Physiol.* 87 355–357. 10.1016/0300-9629(87)90135-6 2886276

[B232] Sola Vigo F. Kedikian G. Heredia L. Heredia F. Anel A. D. Rosa A. L. (2009). Amyloid-beta precursor protein mediates neuronal toxicity of amyloid beta through Go protein activation. *Neurobiol Aging* 30 1379–1392. 10.1016/j.neurobiolaging.2007.11.017 18187234

[B233] Sowell E. R. Peterson B. S. Thompson P. M. Welcome S. E. Henkenius A. L. Toga A. W. (2003). Mapping cortical change across the human life span. *Nat. Neurosci.* 6 309–315. 10.1038/nn1008 12548289

[B234] Sperlágh B. Zsilla G. Baranyi M. Kékes-Szabó A. Vizi E. S. (1997). Age-dependent changes of presynaptic neuromodulation via A1-adenosine receptors in rat hippocampal slices. *Int. J. Dev. Neurosci.* 15 739–747. 10.1016/S0736-5748(97)00028-2 9402224

[B235] Sriram K. Insel P. A. (2018). G protein-coupled receptors as targets for approved drugs: how many targets and how many drugs? *Mol. Pharmacol.* 93 251–258. 10.1124/mol.117.111062 29298813 PMC5820538

[B236] Sternweis P. C. Robishaw J. D. (1984). Isolation of two proteins with high affinity for guanine nucleotides from membranes of bovine brain. *J. Biol. Chem.* 259 13806–13813. 6438083

[B237] Stumm C. Hiebel C. Hanstein R. Purrio M. Nagel H. Conrad A. (2013). Cannabinoid receptor 1 deficiency in a mouse model of Alzheimer’s disease leads to enhanced cognitive impairment despite of a reduction in amyloid deposition. *Neurobiol. Aging* 34 2574–2584. 10.1016/j.neurobiolaging.2013.05.027 23838176

[B238] Sudo H. Hashimoto Y. Niikura T. Shao Z. Yasukawa T. Ito Y. (2001). Secreted Aβ does not mediate neurotoxicity by antibody-stimulated amyloid precursor protein. *Biochem. Biophys. Res. Commun.* 282 548–556. 10.1006/bbrc.2001.4604 11401495

[B239] Sudo H. Jiang H. Yasukawa T. Hashimoto Y. Niikura T. Kawasumi M. (2000). Antibody-regulated neurotoxic function of cell-surface β-amyloid precursor protein. *Mol. Cell. Neurosci.* 16 708–723. 10.1006/mcne.2000.0910 11124892

[B240] Svingos A. L. Chavkin C. Colago E. E. Pickel V. M. (2001). Major coexpression of kappa-opioid receptors and the dopamine transporter in nucleus accumbens axonal profiles. *Synapse* 42 185–192. 10.1002/syn.10005 11746715

[B241] Svízenská I. Dubový P. Sulcová A. (2008). Cannabinoid receptors 1 and 2 (CB1 and CB2), their distribution, ligands and functional involvement in nervous system structures–a short review. *Pharmacol. Biochem. Behav.* 90 501–511. 10.1016/j.pbb.2008.05.010 18584858

[B242] Takkinen J. S. López-Picón F. R. Kirjavainen A. K. Pihlaja R. Snellman A. Ishizu T. (2018). [18F]FMPEP-d2 PET imaging shows age- and genotype-dependent impairments in the availability of cannabinoid receptor 1 in a mouse model of Alzheimer’s disease. *Neurobiol. Aging* 69 199–208. 10.1016/j.neurobiolaging.2018.05.013 29909177

[B243] Tang G. Wang Y. Park S. Bajpayee N. S. Vi D. Nagaoka Y. (2012). Go2 G protein mediates galanin inhibitory effects on insulin release from pancreatic β cells. *Proc. Natl. Acad. Sci. U.S.A.* 109 2636–2641. 10.1073/pnas.1200100109 22308501 PMC3289306

[B244] Tauscher J. Verhoeff N. P. L. Christensen B. K. Hussey D. Meyer J. H. Kecojevic A. (2001). Serotonin 5-HT1A receptor binding potential declines with age as measured by [11C]WAY-100635 and PET. *Neuropsychopharmacology* 24 522–530. 10.1016/S0893-133X(00)00227-X 11282252

[B245] Tayebati S. K. Amenta F. El-Assouad D. Zaccheo D. (2002). Muscarinic cholinergic receptor subtypes in the hippocampus of aged rats. *Mech. Ageing Dev.* 123 521–528. 10.1016/S0047-6374(01)00353-0 11796137

[B246] Tayebati S. K. Di Tullio M. A. Amenta F. (2004). Age-related changes of muscarinic cholinergic receptor subtypes in the striatum of Fisher 344 rats. *Exp. Gerontol.* 39 217–223. 10.1016/j.exger.2003.10.016 15036415

[B247] Tayebati S. K. Vitali D. Scordella S. Amenta F. (2001). Muscarinic cholinergic receptors subtypes in rat cerebellar cortex: light microscope autoradiography of age-related changes. *Brain Res.* 889 256–259. 10.1016/S0006-8993(00)03146-2 11166715

[B248] Thal L. J. Horowitz S. G. Dvorkin B. Makman M. H. (1980). Evidence for loss of brain [3H]spiroperidil and [3H]ADTN binding sites in rabbit brain with aging. *Brain Res.* 192 185–194. 10.1016/0006-8993(80)91018-5 7378779

[B249] Thathiah A. De Strooper B. (2011). The role of G protein-coupled receptors in the pathology of Alzheimer’s disease. *Nat. Rev. Neurosci.* 12 73–87. 10.1038/nrn2977 21248787

[B250] The UniProt Consortium. (2017). UniProt: the universal protein knowledgebase. *Nucleic Acids Res.* 45 D158–D169. 10.1093/nar/gkw1099 27899622 PMC5210571

[B251] Tice M. A. Hashemi T. Taylor L. A. McQuade R. D. (1996). Distribution of muscarinic receptor subtypes in rat brain from postnatal to old age. *Dev. brain Res.* 92 70–76. 10.1016/0165-3806(95)01515-9 8861724

[B252] Tóth A. D. Turu G. Hunyady L. Balla A. (2018). Novel mechanisms of G-protein-coupled receptors functions: AT1 angiotensin receptor acts as a signaling hub and focal point of receptor cross-talk. *Best Pract. Res. Clin. Endocrinol. Metab.* 32 69–82. 10.1016/j.beem.2018.02.003 29678287

[B253] Tribollet E. Audigier S. Dubois-Dauphin M. Dreifuss J. J. (1990). Gonadal steroids regulate oxytocin receptors but not vasopressin receptors in the brain of male and female rats. An autoradiographical study. *Brain Res.* 511 129–140. 10.1016/0006-8993(90)90232-Z 2158853

[B254] Tsuneki H. Sasaoka T. Sakurai T. (2016). Sleep control, GPCRs, and glucose metabolism. *Trends Endocrinol. Metab.* 27 633–642. 10.1016/j.tem.2016.06.011 27461005

[B255] Tuteja N. (2009). Signaling through G protein coupled receptors. *Plant Signal. Behav.* 4 942–947. 10.4161/psb.4.10.9530 19826234 PMC2801357

[B256] Uhlén M. Fagerberg L. Hallström B. M. Lindskog C. Oksvold P. Mardinoglu A. (2015). Proteomics. Tissue-based map of the human proteome. *Science* 347:1260419. 10.1126/science.1260419 25613900

[B257] Valerio A. Belloni M. Gorno M. L. Tinti C. Memo M. Spano P. (1994). Dopamine D2, D3, and D4 receptor mRNA levels in rat brain and pituitary during aging. *Neurobiol. Aging* 15 713–719. 10.1016/0197-4580(94)90053-1 7891826

[B258] van Biesen T. Hawes B. E. Raymond J. R. Luttrell L. M. Koch W. J. Lefkowitz R. J. (1996). G(o)-protein alpha-subunits activate mitogen-activated protein kinase via a novel protein kinase C-dependent mechanism. *J. Biol. Chem.* 271 1266–1269. 10.1074/jbc.271.3.1266 8576109

[B259] van Gastel J. Boddaert J. Jushaj A. Premont R. T. Luttrell L. M. Janssens J. (2018). GIT2-A keystone in ageing and age-related disease. *Ageing Res. Rev.* 43 46–63. 10.1016/j.arr.2018.02.002 29452267

[B260] Van Laere K. Goffin K. Casteels C. Dupont P. Mortelmans L. de Hoon J. (2008). Gender-dependent increases with healthy aging of the human cerebral cannabinoid-type 1 receptor binding using [(18)F]MK-9470 PET. *Neuroimage* 39 1533–1541. 10.1016/j.neuroimage.2007.10.053 18077184

[B261] Verdurand M. Zimmer L. (2017). Hippocampal 5-HT 1A receptor expression changes in prodromal stages of Alzheimer’s disease: beneficial or deleterious? *Neuropharmacology* 123 446–454. 10.1016/j.neuropharm.2017.06.021 28647411

[B262] Villar-Cheda B. Dominguez-Meijide A. Valenzuela R. Granado N. Moratalla R. Labandeira-Garcia J. L. (2014). Aging-related dysregulation of dopamine and angiotensin receptor interaction. *Neurobiol. Aging* 35 1726–1738. 10.1016/j.neurobiolaging.2014.01.017 24529758

[B263] Villar-Cheda B. Valenzuela R. Rodriguez-Perez A. I. Guerra M. J. Labandeira-Garcia J. L. (2012). Aging-related changes in the nigral angiotensin system enhances proinflammatory and pro-oxidative markers and 6-OHDA-induced dopaminergic degeneration. *Neurobiol. Aging* 33:204.e1-e11. 10.1016/j.neurobiolaging.2010.08.006 20888078

[B264] Vuong C. Van Uum S. H. M. O’Dell L. E. Lutfy K. Friedman T. C. (2010). The effects of opioids and opioid analogs on animal and human endocrine systems. *Endocr. Rev.* 31 98–132. 10.1210/er.2009-0009 19903933 PMC2852206

[B265] Wallace D. R. Booze R. M. (1996). Dopamine D3 receptor density elevation in aged Fischer-344 x Brown-Norway (F1) rats. *Eur. J. Pharmacol.* 308 283–285. 10.1016/0014-2999(96)00354-8 8858300

[B266] Wang G. J. Volkow N. D. Logan J. Fowler J. S. Schlyer D. MacGregor R. R. (1995). Evaluation of age-related changes in serotonin 5-HT2 and dopamine D2 receptor availability in healthy human subjects. *Life Sci.* 56 L249–L253. 10.1016/0024-3205(95)00066-F 7475891

[B267] Wang Y. Park S. Bajpayee N. S. Nagaoka Y. Boulay G. Birnbaumer L. (2011). Augmented glucose-induced insulin release in mice lacking G(o2), but not G(o1) or G(i) proteins. *Proc. Natl. Acad. Sci. U.S.A.* 108 1693–1698. 10.1073/pnas.1018903108 21220323 PMC3029743

[B268] Weiss B. Chen J. F. Zhang S. Zhou L. W. (1992). Developmental and age-related changes in the D2 dopamine receptor mRNA subtypes in rat brain. *Neurochem. Int.* 20(Suppl.), 49S–58S. 10.1016/0197-0186(92)90210-I 1365455

[B269] Whorton M. R. MacKinnon R. (2013). X-ray structure of the mammalian GIRK2-βγ G-protein complex. *Nature* 498 190–197. 10.1038/nature12241 23739333 PMC4654628

[B270] Wong D. F. Kuwabara H. Horti A. G. Raymont V. Brasic J. Guevara M. (2010). Quantification of cerebral cannabinoid receptors subtype 1 (CB1) in healthy subjects and schizophrenia by the novel PET radioligand [11C]OMAR. *Neuroimage* 52 1505–1513. 10.1016/j.neuroimage.2010.04.034 20406692 PMC6580862

[B271] Wong D. F. Wagner H. N. Jr. Dannals R. F. Links J. M. Frost J. J. Ravert H. T. (1984). Effects of age on dopamine and serotonin receptors measured by positron tomography in the living human brain. *Science* 226 1393–1396. 10.1126/science.6334363 6334363

[B272] Xu Y.-X. Wang H.-Q. Yan J. Sun X.-B. Guo J.-C. Zhu C.-Q. (2009). Antibody binding to cell surface amyloid precursor protein induces neuronal injury by deregulating the phosphorylation of focal adhesion signaling related proteins. *Neurosci. Lett.* 465 276–281. 10.1016/j.neulet.2009.09.022 19766167

[B273] Yamatsuji T. Matsui T. Okamoto T. Komatsuzaki K. Takeda S. Fukumoto H. (1996a). G protein-mediated neuronal DNA fragmentation induced by familial Alzheimer’s disease-associated mutants of APP. *Science* 272 1349–1352. 10.1126/science.272.5266.1349 8650548

[B274] Yamatsuji T. Okamoto T. Takeda S. Murayama Y. Tanaka N. Nishimoto I. (1996b). Expression of V642 APP mutant causes cellular apoptosis as Alzheimer trait-linked phenotype. *Embo J.* 15 498–509. 8599933 PMC449968

[B275] Yew D. T. Yeung L.-Y. Wai M. S.-M. Mak Y. T. (2009). 5-HT 1A and 2A receptor positive cells in the cerebella of mice and human and their decline during aging. *Microsc. Res. Tech.* 72 684–689. 10.1002/jemt.20717 19353636

[B276] Young L. T. Warsh J. J. Li P. P. Siu K. P. Becker L. Gilbert J. (1991). Maturational and aging effects on guanine nucleotide binding protein immunoreactivity in human brain. *Brain Res. Dev. Brain Res.* 61 243–248. 10.1016/0165-3806(91)90137-8 1752042

[B277] Yudin Y. Rohacs T. (2018). Inhibitory G i/O -coupled receptors in somatosensory neurons: potential therapeutic targets for novel analgesics. *Mol. Pain* 14:174480691876364. 10.1177/1744806918763646 29580154 PMC5882016

[B278] Zhang F. Challapalli S. C. Smith P. J. W. (2009). Cannabinoid CB1 receptor activation stimulates neurite outgrowth and inhibits capsaicin-induced Ca2+ influx in an in vitro model of diabetic neuropathy. *Neuropharmacology* 57 88–96. 10.1016/j.neuropharm.2009.04.017 19501110

[B279] Zhao J. Deng Y. Jiang Z. Qing H. (2016). G Protein-Coupled Receptors (GPCRs) in Alzheimer’s Disease: a Focus on BACE1 Related GPCRs. *Front. Aging Neurosci.* 8:58 10.3389/fnagi.2016.00058 PMC4805599 27047374

[B280] Zhou Y. Danbolt N. C. (2014). Glutamate as a neurotransmitter in the healthy brain. *J. Neural Transm.* 121 799–817. 10.1007/s00702-014-1180-8 24578174 PMC4133642

