# Supplementary material for: Gi/o-Protein Coupled Receptors in the Aging Brain
Source: Front Aging Neurosci. 2019 Apr 24;11:89. doi: 10.3389/fnagi.2019.00089 (PMC6492497; doi:10.3389/fnagi.2019.00089)
Supplement: Supplementary file 1 [file Table_1.docx]

**Supplementary Table S1:** GPCRs that can couple to the Gi/o subfamily, and their localization in several tissues of the human body (spanning tissues of several ages). Data was taken from ‘The Human Protein Atlas database’ (v16.1.proteinatlas.org). Human Protein Atlas expression score is based on immunohistochemical data scored with regard to staining intensity (classified as negative, weak, moderate or strong) and the fraction of stained cells in the tissue (<25%, 25-75% or >75%). Each combination of intensity and fractions is automatically converted into a protein expression level score as follows: **not detected (N.D.),** if negative OR combination of weak and <25%; **low,** if weak combined with either 25-75% or 75% OR moderate combined with <25%; **medium**, if moderate combined with either 25-75% or 75% OR strong combined with <25%; **high**, if strong combined with either 25-75% or 75%. **Not available (N.A.).**

| **Receptors** | | **Brain** | **Endocrine tissues** | **Bone marrow and immune system** | **Muscle tissues** | **Lung** | **Liver and gallbladder** | **Pancreas** | **Gastrointestinal tract** | **Kidney and Urinary bladder** | **Male tissues** | **Female tissues** | **Adipose and soft tissue** | **Skin** |
| --- | --- | --- | --- | --- | --- | --- | --- | --- | --- | --- | --- | --- | --- | --- |
| Adrenoceptors | α2A | N.A. | | | | | | | | | | | | |
|  | α2B | high | N.D. | N.D. | low | low | low | N.D. | N.D. | N.D. | low | N.D. | low | N.D. |
|  | α2C | N.A. | | | | | | | | | | | | |
| Histamine receptors | HRH3 | N.A. | | | | | | | | | | | | |
|  | HRH4 | N.A. | | | | | | | | | | | | |
| Dopamine receptors | D2 | medium | N.D. | N.D. | N.D. | N.D. | N.D. | N.D. | N.D. | N.D. | N.D. | N.D. | N.D. | N.D. |
|  | D3 | N.A. | | | | | | | | | | | | |
|  | D4 | N.A. | | | | | | | | | | | | |
| Acetylcholine receptors | M2 | high | high | high | medium | high | high | High | high | high | high | high | low | high |
|  | M4 | N.A. | | | | | | | | | | | | |
| Serotonin receptors | 5-HT1A | high | medium | low | medium | low | medium | N.D. | high | high | low | low | low | N.D. |
|  | 5-HT1B | low | N.D. | N.D. | N.D. | N.D. | N.D. | N.D. | low | N.D. | N.D. | low | N.D. | N.D. |
|  | 5-HT1D | N.A. | | | | | | | | | | | | |
|  | 5-HT1E | high | medium | high | medium | high | medium | medium | high | high | high | medium | medium | medium |
|  | 5-HT1F | N.A. | | | | | | | | | | | | |
|  | 5-HT5A | low | low | low | N.D. | low | low | Low | low | low | low | low | low | low |
| Melatonin receptors | MTNR1A | N.A. | | | | | | | | | | | | |
|  | MTNR1B | N.A. | | | | | | | | | | | | |
| Adenosine receptors (P1) | A1 | N.A. | | | | | | | | | | | | |
|  | A3 | N.A. | | | | | | | | | | | | |
| Purinoceptors (P2) | P2Y12 | medium | N.D. | low | N.D. | N.D. | N.D. | N.D. | N.D. | N.D. | N.D. | low | N.D. | N.D. |
|  | P2Y13 | low | N.D. | high | N.D. | low | N.D. | N.D. | N.D.^1^ | N.D. | N.D. | N.D. | N.D. | N.D. |
|  | P2Y14 | medium | medium | medium | medium | medium | medium | medium | medium | medium | medium | high | low | medium |
| Opioid receptors | δ | N.A. | | | | | | | | | | | | |
|  | κ | low | N.D. | N.D. | N.D. | N.D. | N.D. | N.D. | N.D. | N.D. | low | N.D. | N.D. | N.D. |
|  | μ | N.D. | N.D. | N.D. | N.D. | N.D. | N.D. | N.D. | N.D .^2^ | N.D. | medium | N.D. | N.D. | N.D. |
|  | OPRL1 | N.A. | | | | | | | | | | | | |
| Somatostatin receptors | SSTR1 | medium | low | medium | N.D. | low | low | medium | high | medium | medium | low | low | low |
|  | SSTR2 | high | medium | medium | N.D. | medium | medium | medium | medium | medium | medium | medium | medium | medium |
|  | SSTR3 | high | N.D. | N.D. | N.D. | N.D. | N.D. | N.D. | low | N.D. | medium | N.D. | N.D. | N.D. |
|  | SSTR4 | N.A. | | | | | | | | | | | | |
|  | SSTR5 | N.A. | | | | | | | | | | | | |
| Melanin-concentrating hormone receptors | MCHR1 | N.A. | | | | | | | | | | | | |
| Neuropeptide Y receptors | NPY1R | N.A. | | | | | | | | | | | | |
|  | NPY2R | N.A. | | | | | | | | | | | | |
|  | NPY4R | low | low | low | low | low | low | low | medium | low | low | low | low | medium |
|  | NPY5R | N.A. | | | | | | | | | | | | |
| Angiotensin receptors | AT1 | low | N.D .^3^ | N.D. | N.D. | N.D. | N.D. | N.D. | N.D. | N.D. | N.D. | N.D. | high | N.D. |
| Vasopressin/oxytocin receptors | OT | N.A. | | | | | | | | | | | | |
| Endothelin receptors | ETB | N.D. | N.D. | low | low | low | N.D. | N.D. | low | low | low | medium | N.D. | low |
| Neuropeptide FF receptor | NPFFR1 | N.A. | | | | | | | | | | | | |
|  | NPFFR2 | N.A. | | | | | | | | | | | | |
| Galanin receptors | GALR1 | N.A. | | | | | | | | | | | | |
|  | GALR2 | medium | medium | medium | medium | medium | medium | medium | medium | medium | medium | medium | low | medium |
|  | GALR3 | N.A. | | | | | | | | | | | | |
| Proteinase-activated receptors | PAR-1 | low | N.D. | medium | N.D. | medium | N.D. | N.D. | low | medium | medium | medium | low | medium |
|  | PAR-4 | N.A. | | | | | | | | | | | | |
| Neuropeptide B/W receptors | NPBWR1 | N.A. | | | | | | | | | | | | |
|  | NPBWR2 | N.A. | | | | | | | | | | | | |
| Apelin receptor | APJ | medium | medium | medium | medium | low | medium | N.D. | medium | low | medium | low | low | low |
| Chemokine receptors | CCR1 | N.A.. | | | | | | | | | | | | |
|  | CCR2 | low | N.D. | high | low | low | low | N.D. | low | low | low | medium | N.D. | low |
|  | CCR3 | low | medium | low | N.D. | medium | medium | N.D. | medium | low | low | low | low | low |
|  | CCR4 | medium | N.D. | medium | low | low | low | medium | medium | low | low | low | low | N.D. |
|  | CCR5 | N.A. | | | | | | | | | | | | |
|  | CCR6 | N.D. | N.D. | high | N.D. | N.D. | N.D. | N.D. | N.D. | N.D. | N.D. | N.D. | N.D. | N.D. |
|  | CCR7 | N.D. | N.D. | high | N.D. | high | N.D. | N.D. | N.D. | N.D. | N.D. | N.D. | N.D. | N.D. |
|  | CCR8 | N.A. | | | | | | | | | | | | |
|  | CCR9 | N.D. | N.D. | low | N.D. | N.D. | N.D. | N.D. | N.D. | N.D. | N.D. | N.D. | N.D. | N.D. |
|  | CCR10 | medium | medium | medium | medium | medium | low | low | medium | low | low | low | N.D. | low |
|  | CXCR1 | low | low | medium | low | low | low | low | low | low | low | low | N.D. | low |
|  | CXCR2 | N.D. | N.D. | medium | N.D. | N.D. | N.D. | N.D. | N.D. | N.D. | N.D. | N.D. | N.D. | N.D. |
|  | CXCR3 | N.D. | N.D. | medium | low | low | N.D. | N.D. | N.D .^4^ | N.D. | N.D. | N.D. | N.D. | N.D. |
|  | CXCR4 | N.A. | | | | | | | | | | | | |
|  | CXCR5 | medium | medium | high | medium | medium | low | medium | medium | medium | medium | medium | medium | medium |
|  | CXCR6 | N.A. | | | | | | | | | | | | |
|  | CXCR7 | N.D. | medium | medium | low | low | low | medium | low | medium | low | medium | low | low |
|  | CX3CR1 | medium | medium | medium | low | medium | medium | N.D. | medium | medium | medium | medium | low | low |
|  | XCR1 | N.A. | | | | | | | | | | | | |
| (Glyco)protein hormone receptors | LSH | N.A. | | | | | | | | | | | | |
|  | TSH | N.D. | high | N.D. | N.D. | N.D. | N.D. | N.D. | N.D. | N.D. | N.D. | N.D. | N.D. | N.D. |
| Cannabinoid receptors | CB1 | high | high | high | low | high | medium | low | medium | low | medium | high | medium | low |
|  | CB2 | medium | high | high | medium | medium | medium | medium | high | high | medium | high | low | high |
| Prostanoid receptors | DP2 | low | low | N.D. | N.D. | N.D. | low | high | low | N.D. | low | N.D. | N.D. | N.D. |
|  | EP3 | N.D. | N.D. | N.D. | medium | N.D. | N.D. | N.D. | N.D. | low | N.D. | high | N.D. | N.D. |
| Leukotriene receptors | LTB4R | low | N.D .^5^ | medium | N.D. | low | N.D. | N.D. | N.D. | N.D. | N.D. | N.D. | low | N.D. |
|  | LTB4R2 | N.A. | | | | | | | | | | | | |
| Chemotactic substances | C3a | N.D. | low | medium | low | low | N.D. | low | low | N.D. | low | low | low | N.D. |
|  | C5a | medium | medium | high | N.D. | medium | low | N.D. | medium | low | low | low | low | low |
|  | FPR | N.D. | N.D. | medium | low | low | N.D. | N.D. | low | N.D. | low | N.D. | N.D. | low |
|  | FPR2 | low | medium | medium | N.D. | N.D. | low | low | high | N.D. | N.D. | medium | N.D. | low |
| Lysophospholipid receptors | S1P1 | high | high | high | medium | high | medium | low | high | high | medium | high | low | high |
|  | S1P2 | N.D. | N.D. | N.D. | N.D. | N.D. | N.D. | N.D. | high | medium | N.D. | N.D. | N.D. | N.D. |
|  | S1P3 | N.A. | | | | | | | | | | | | |
|  | S1P4 | N.A. | | | | | | | | | | | | |
|  | S1P5 | low | N.D. | medium | N.D. | low | N.D. | N.D. | N.D. | N.D. | N.D. | N.D. | N.D. | high |
|  | LPA1 | N.A. | | | | | | | | | | | | |
|  | LPA2 | high | medium | medium | medium | medium | high | medium | high | medium | high | medium | low | low |
|  | LPA3 | medium | medium | high | medium | medium | medium | medium | medium | high | high | medium | medium | medium |
|  | LPA5 | medium | medium | high | medium | medium | medium | medium | high | medium | medium | medium | medium | low |
| Free fatty acid receptors | GPR41 | low | low | low | low | low | low | low | low | low | low | low | low | low |
|  | GPR43 | medium | N.D. | high | N.D. | medium | N.D. | low | low | N.D. | low | low | N.D. | low |
|  | GPR84 | medium | low | high | low | N.D .^6^ | N.D. | N.D. | low | low | low | N.D. | low | low |
| Dicarboxylate receptors  (Succinate receptor 1) | GPR91 | N.A. | | | | | | | | | | | | |
| Ketone body receptor  (Hydroxycarboxylic acid receptor) | GPR109A | low | low | medium | low | low | low | N.D. | low | medium | medium | low | low | low |
|  | GPR109B | low | low | medium | low | medium | low | N.D. | low | low | low | low | low | low |
| Oxoeicosanoid receptor 1 | OLOWER1 | medium | medium | medium | medium | medium | medium | medium | high | medium | medium | high | medium | high |
| N-arachidonyl glycine receptor | GPR18 | N.D. | N.D. | medium | N.D. | N.D. | N.D. | N.D. | N.D. | N.D. | N.D. | N.D. | N.D. | N.D. |
| G-protein coupled receptor 35 | GPR35 | N.A. | | | | | | | | | | | | |
| Metabotropic GABA receptors | GABAB2 | high | N.D. | N.D. | N.D. | N.D. | N.D. | N.D. | low | N.D. | N.D. | N.D. | N.D. | N.D. |
| Metabotropic glutamate receptors | mGluR2 | medium | N.D. | N.D. | N.D. | N.D. | N.D. | N.D. | N.D. | N.D. | N.D. | N.D. | N.D. | N.D. |
|  | mGluR3 | N.A. | | | | | | | | | | | | |
|  | mGluR4 | high | N.D. | N.D. | N.D. | N.D. | N.D. | N.D. | N.D. | N.D. | N.D. | N.D. | N.D. | N.D. |
|  | mGluR6 | low | N.D. | N.D. | N.D. | N.D. | N.D. | N.D. | low | N.D. | N.D. | N.D. | N.D. | N.D. |
|  | mGluR7 | N.A. | | | | | | | | | | | | |
|  | mGluR8 | N.A. | | | | | | | | | | | | |

^1^except for oral mucosa – N.A.; ^2^except for small intestine – N.A.; ^3^except for parathyroid gland – N.A.; ^4^except for small intestine – N.A.; ^5^except for parathyroid gland – N.A., ^6^except for bronchus – N.A.
