# Supplementary material for: Gi/o-Protein Coupled Receptors in the Aging Brain
Source: Front Aging Neurosci. 2019 Apr 24;11:89. doi: 10.3389/fnagi.2019.00089 (PMC6492497; doi:10.3389/fnagi.2019.00089)
Supplement: Supplementary file 2 [file Table_2.docx]

**Supplementary Table S2:** Age-related alterations in the protein density and affinity to ligands (or other compounds) of brain Gi/Go-coupled GPCRs. Information on alterations in mRNA levels, in Binding potential and in Affinity of ligand binding were also included when available. Binding potential is the *B_max_* to *K_D_* ratio, with *B_max_* being the total density (a measure of concentration) of receptors in a sample of tissue, and *K_D_* the equilibrium dissociation constant of the radioligand. Affinity of ligand binding is the inverse of *K_D_*. RT-PCR - Reverse Transcription Polymerase Chain Reaction; qPCR – quantitative (‘real-time’) PCR.

|  |  |  |  | **Age-associated alterations in:** | | |  | |
| --- | --- | --- | --- | --- | --- | --- | --- | --- |
| **Receptors Classes** | **Brain Region** | **Animal** | **Method; receptor subtype analyzed** | **Protein Density** | **Affinity to ligand / other** | **Others, including**  **Binding Potential** | **Reference** | |
| Adrenoceptors | Hindbrain (Rhombencephalon) | Male New Zealand White Rabbits (1 month-3 years) | [^3^H]clonidine binding; **α2** | Decreased | Increased |  | (Hamilton et al., 1984) | |
|  | Cerebellum – Stratum granulare | Human *(postmortem)* (20-70 years) | [^3^H]UK-14304 binding (Autoradiography); **α2** | No alteration |  |  | (Pascual et al., 1991) | |
|  | Lateral periaqueductal area |  |  | No alteration |  |  | (Pascual et al., 1991) | |
|  | Interpeduncular nucleus |  |  | No alteration |  |  | (Pascual et al., 1991) | |
|  | Forebrain (Prosencephalon) | Male New Zealand White Rabbits (1 month-3 years) | [^3^H]clonidine binding; **α2** | Decreased |  |  | (Hamilton et al., 1984) | |
|  | Hypothalamus – Ventromedial nucleus | Human *(postmortem)* (20-70 years) | [^3^H]UK-14304 binding (Autoradiography); **α2** | No alteration |  |  | (Pascual et al., 1991) | |
|  | Hippocampus – CornuAmmonis area 1 |  |  | No alteration |  |  | (Pascual et al., 1991) | |
|  | Amygdala – Nucleus medialis and basalis accesorius |  |  | No alteration |  |  | (Pascual et al., 1991) | |
|  | Claustrum |  |  | Decreased |  |  | (Pascual et al., 1991) | |
|  | Basal Ganglia – Striatum – Caudate and Putamen |  |  | Decreased |  |  | (Pascual et al., 1991) | |
|  | Basal Ganglia –  Nucleus basalis of Meynert |  |  | Decreased |  |  | (Pascual et al., 1991) | |
|  | Cerebral cortex | Wistar rats (70 days and 2 years) | [^3^H]clonidine binding; **α2** | No alteration | No alteration |  | (Nomura et al., 1986) | |
|  | Cerebral cortex – Frontal cortex – Layer I | Human *(postmortem)* (20-70 years) | [^3^H]UK-14304 binding (Autoradiography); **α2** | Decreased |  |  | (Pascual et al., 1991) | |
|  | Cerebral cortex – Frontal cortex – Prefrontal cortex | Rhesus monkeys *(Macaca mulatta)* (3-10 years and >20 years) | [^3^H]clonidine binding (Autoradiography); **α2** | Decreased (Layer I) | No alteration |  | (Bigham and Lidow, 1995) | |
|  |  | Human *(postmortem)* (1-88 years) | UK14304 stimulated [^35^S]GTPγS binding; **α2** |  |  | Decreased agonist (UK14304) stimulated [^35^S]GTPγS binding | (González-Maeso et al., 2002) | |
|  | Cerebral cortex - Frontal cortex – Primary motor cortex - Layer I-V | Rhesus monkeys *(Macaca mulatta)* (3-10 years and >20 years) | [^3^H]clonidine binding (Autoradiography); **α2** | Decreased | No alteration |  | (Bigham and Lidow, 1995) | |
|  | Cerebral cortex - Parietal cortex – Somatosensorycortex – Layer I-V |  |  | Decreased | No alteration |  | (Bigham and Lidow, 1995) | |
|  | Cerebral cortex -Temporal cortex-Layer I | Human *(postmortem)* (20-70 years) | [^3^H]UK-14304 binding (Autoradiography); **α2** | Decreased |  |  | (Pascual et al., 1991) | |
|  | Cerebral cortex – Occipital cortex - Primary visual cortex |  |  | Decreased (Layer III) |  |  | (Pascual et al., 1991) | |
|  |  | Rhesus monkeys *(Macaca mulatta)* (3-10 years and >20 years) | [^3^H]clonidine binding (Autoradiography); **α2** | No alteration | No alteration |  | (Bigham and Lidow, 1995) | |
|  | Brainstem -  Trigeminal nucleus | Human *(postmortem)* (20-70 years) | [^3^H]UK-14304 binding (Autoradiography); **α2** | No alteration |  |  | (Pascual et al., 1991) | |
| Dopamine Receptors | Cerebellum | Male Fischer 344 rats (6 and 24 months) | [^3^H]spiperone binding (Autoradiography); **D2**  [^3^H]nemonapride binding (Autoradiography); **D2** | No alteration |  |  | (Araki et al., 1997) | |
|  |  | Male Wistar rats (3,12 and 24 months) | [^3^H]spiroperidol binding (Autoradiography); **D2-like** (probably D3) | Decreased | No alteration |  | (Ricci et al., 1996) | |
|  | Corpora quadrigemina – Inferior Colliculus | Fischer 344 rats (3 and 24 months) | [^35^S]-labelled mRNA hybridization and Northern blot; **D2** |  |  | Decreased D2 mRNA levels | (Weiss et al., 1992) | |
|  | Substantia nigra | Male Fischer 344 rats (6 and 24 months) | [^3^H]spiperone binding (Autoradiography); **D2**  [^3^H]nemonapride binding (Autoradiography); **D2** | No alteration |  |  | (Araki et al., 1997) | |
|  |  | Male Wistar-Kyoto rats (6 and 24 months) | RT-PCR (mRNA); **D2** |  |  | Decreased D2 mRNA levels | (Valerio et al., 1994) | |
|  |  | Human (18-73 years) | [^11^C]-(+)4-Propyl- 3,4,4a,5,6,10b-hexahydro-*2H*-naphtho[1,2-b][1,4]oxazin-9-ol binding (PET); **D3** |  |  | No alteration in nondisplaceable binding potential of an agonist | (Nakajima et al., 2015) | |
|  |  | Human *(postmortem)* (0-20 and >65 years years) | [^3^H]spiroperidol binding; **D2** | Decreased | No alteration |  | (Rinne, 1987) | |
|  |  | Male Sprague-Dawley  rats (10 weeks and 18-20 months) | qPCR (mRNA); **D2**  Western Blot; **D2** | Decreased |  | Decreased D2 mRNA levels | (Villar-Cheda et al., 2014) | |
|  | Substantia nigra - Ventral Tegmental area | Human (19-55 years) | [^11^C]-(+)4-Propyl- 3,4,4a,5,6,10b-hexahydro-*2H*-naphtho[1,2-b][1,4]oxazin-9-ol binding (PET); **D3** |  |  | No alteration in binding potential of an agonist | (Matuskey et al., 2016) | |
|  |  |  | [^11^C]-(+)4-Propyl- 3,4,4a,5,6,10b-hexahydro-*2H*-naphtho[1,2-b][1,4]oxazin-9-ol binding (Whole-brain voxel-wise analysis); **D3** |  |  | Increased binding potential of an agonist | (Matuskey et al., 2016) | |
|  | Forebrain - Anterior Limbic cortex | New Zealand White Rabbits (5 month and 5.5 years) | [^3^H]spiroperidol binding; **Dopamine receptors** | Decreased | No alteration |  | (Thal et al., 1980) | |
|  | Thalamus | Human (21-82 years) | [^11^C]FLB 457 binding (PET); **D2** |  |  | Decreased binding potential of an antagonist | (Inoue et al., 2001) | |
|  |  | Human (19-55 years) | [^11^C]-(+)4-Propyl- 3,4,4a,5,6,10b-hexahydro-*2H*-naphtho[1,2-b][1,4]oxazin-9-ol binding (PET); **D2/3** |  |  | No alteration in binding potential of an agonist | (Matuskey et al., 2016) | |
|  | Thalamus – Medial and Lateral Nuclear group | Human (19-74 years) | [^11^C]FLB 457 binding (PET); **D2; D3** |  |  | Decreased binding potential of an antagonist | (Kaasinen et al., 2000) | |
|  | Hypothalamus | Human (18-73 years) | [^11^C]-(+)4-Propyl- 3,4,4a,5,6,10b-hexahydro-*2H*-naphtho[1,2-b][1,4]oxazin-9-ol binding (PET); **D3** |  |  | No alteration in nondisplaceable binding potential of an agonist | (Nakajima et al., 2015) | |
|  |  | Human (19-55 years) | [^11^C]-(+)4-Propyl- 3,4,4a,5,6,10b-hexahydro-*2H*-naphtho[1,2-b][1,4]oxazin-9-ol binding (PET); **D3** |  |  | No alteration in binding potential of an agonist | (Matuskey et al., 2016) | |
|  |  |  | [^11^C]-(+)4-Propyl- 3,4,4a,5,6,10b-hexahydro-*2H*-naphtho[1,2-b][1,4]oxazin-9-ol binding (Whole-brain voxel-wise analysis); **D3** |  |  | No alteration in binding potential of an agonist | (Matuskey et al., 2016) | |
|  | Anterior Pituitary | Male Wistar-Kyoto rats (6 and 24 months) | RT-PCR (mRNA); **D2** |  |  | Increased D2 mRNA levels | (Valerio et al., 1994) | |
|  |  |  | RT-PCR (mRNA); **D4** |  |  | No alteration in D4 mRNA levels | (Valerio et al., 1994) | |
|  | Pituitary Gland – Anterior and intermediate lobes | Fischer 344 rats (3 and 24 months) | [^35^S]-mRNA hybridization and Northern blot; **D2** |  |  | Increased D2 mRNA levels | (Weiss et al., 1992) | |
|  | Hippocampus | Male Wistar-Kyoto rats (6 and 24 months) | RT-PCR (mRNA); **D2** |  |  | No alteration in D2 mRNA levels | (Valerio et al., 1994) | |
|  |  | Human (21-82 years) | [^11^C]FLB 457 binding (PET); **D2** |  |  | Decreased binding potential of an antagonist | (Inoue et al., 2001) | |
|  |  | Human (19-74 years) | [^11^C]FLB 457 binding (PET)**; D2; D3** |  |  | Decreased binding potential of an antagonist | (Kaasinen et al., 2000) | |
|  | Hippocampus – CornuAmmonis area 1 | Male Fischer 344 rats (6 and 24 months) | [^3^H]spiperone binding (Autoradiography); **D2**  [^3^H]nemonapride binding (Autoradiography); **D2** | No alteration |  |  | (Araki et al., 1997) | |
|  | Amygdala | Human (19-74 years) | [^11^C]FLB 457 binding (PET); **D2; D3** |  |  | Decreased binding potential of an antagonist | (Kaasinen et al., 2000) | |
|  |  | Human (19-55 years) | [^11^C]-(+)4-Propyl- 3,4,4a,5,6,10b-hexahydro-*2H*-naphtho[1,2-b][1,4]oxazin-9-ol binding (PET); **D2/3** |  |  | No alteration in binding potential of an agonist | (Matuskey et al., 2016) | |
|  | Basal Ganglia - Striatum | Male Sprague-Dawley rats (4 and 22 months) | [^3^H]spiperone binding; **D2** | Decreased | No alteration |  | (O’Boyle and Waddington, 1984) | |
|  |  | Male Wistar rats (3, 12, and 24 months) |  | Decreased | No alteration |  | (Henry et al., 1986) | |
|  |  | Male Wistar rats (3-6 and  24-26 months) | [^3^H]spiperone binding (Autoradiography); **D2** | Decreased |  |  | (Han et al., 1989) | |
|  |  | Male Wistar rats (6 months and 24-25 months) | [^35^S]-labelled D2 cDNA hybridization (mRNA); **D2** |  |  | Decreased D2 mRNA levels | (Mesco et al., 1991) | |
|  |  | Male Fischer 344 rats (6 and 24 months) | [^3^H]spiperone binding (Autoradiography); **D2**  [^3^H]nemonapride binding (Autoradiography); **D2** | No alteration |  |  | (Araki et al., 1997) | |
|  |  | Fischer 344 rats (3 and 24 months) | [^35^S]-labelled mRNA hybridization and Northern blot; **D2** |  |  | Decreased D2 mRNA levels | (Weiss et al., 1992) | |
|  |  | Male Wistar-Kyoto rats (6 and 24 months) | RT-PCR (mRNA); **D2** |  |  | Decreased D2 mRNA levels | (Valerio et al., 1994) | |
|  |  |  | RT-PCR (mRNA); **D3** |  |  | No alteration in D3 of mRNA levels | (Valerio et al., 1994) | |
|  |  | New Zealand White Rabbits (5 month and 5.5 years) | [^3^H]spiroperidol binding; **Dopamine receptors** | Decreased | No alteration |  | (Thal et al., 1980) | |
|  |  | Male Fischer 344 x Brown-Norway rats (F1) (4 and 37 months) | [3H](+)-7-hydroxy-2-(*N, N*-di-*n*-propylamino)tetralin; **D3** | Increased |  |  | (Wallace and Booze, 1996) | |
|  |  | Human (20-81 years) | [^11^C]raclopride binding (PET); **D2** | Decreased | No alteration |  | (Rinne et al., 1993) | |
|  |  | Human (19-82 years) | [^11^C]raclopride binding (PET); **D2** | Decreased | No alteration (right striatum);  Decreased (women’s left striatum) | Decreased binding potential of an antagonist | (Pohjalainen et al., 1998) | |
|  |  | Male Human (21-49 years) | F-18-N-methylspiperone (PET); **D2** | Decreased |  |  | (Wang et al., 1995) | |
|  |  | Human (*postmortem*) (19-88 years) | [^3^H]spiperone binding; **D2** | No alteration | No alteration |  | (De Keyser et al., 1990) | |
|  |  | Human (*postmortem*) (7-104 years) |  | Decreased (men);  No alteration (women) |  |  | (Seeman et al., 1987) | |
|  |  | Male Sprague-Dawley  rats (10 weeks and 18-20 months) | qPCR (mRNA); **D2**  Western Blot; **D2** | Decreased |  | Decreased D2 mRNA levels | (Villar-Cheda et al., 2014) | |
|  | Basal Ganglia – Striatum – Caudate and Putamen | Fischer 344 rats (5-6 and 26-28 months) | [^3^H]spiroperidol binding (Autoradiography); **D2** | Decreased | No alteration |  | (Joyce et al., 1986) | |
|  |  | Rhesus Monkeys *(Macaca mulatta)* (2-22 years) | [^3^H]spiroperidol binding; **D2** | Decreased | No alteration |  | (Lai et al., 1987) | |
|  |  | Human (18-73 years) | [^11^C]-(+)4-Propyl- 3,4,4a,5,6,10b-hexahydro-*2H*-naphtho[1,2-b][1,4]oxazin-9-ol and [^11^C]raclopride binding (PET); **D2** |  |  | Decreased nondisplaceable binding potential by [^11^C]raclopride (antagonist) (putamen and caudate); Decreased nondisplaceable binding potential by [^11^C]-(+)-PHNO (agonist) (caudate) and no alteration (putamen) | (Nakajima et al., 2015) | |
|  |  | Human (21-68 years) | [^11^C]raclopride binding (PET); **D2** | Decreased |  |  | (Antonini A et al., 1993) | |
|  |  | Human (19-73 years) | [^11^C]3-N-methylspiperone binding (PET); **D2** | Decreased |  |  | (Wong et al., 1984) | |
|  |  | Human (21-74 years) | [^11^C]raclopride binding (PET); **D2-like** | Decreased |  | Decreased uptake ratio index and binding potential of an antagonist | (Ishibashi et al., 2009) | |
|  |  | Human (*postmortem*) (0-20 and >65 years years) | [^3^H]spiroperidol binding; **D2** | Decreased | No alteration |  | (Rinne, 1987) | |
|  |  | Human (*postmortem*) (6-93 years) |  | Decreased | No alteration |  | (Rinne et al., 1990) | |
|  |  | Human (*postmortem*) (<40 and >70 years) | [^3^H]fluphenazine binding; **D2** | Decreased (caudate);  No alteration (putamen) | No alteration |  | (Morgan et al., 1987) | |
|  |  | Human (19-55 years) | [^11^C]-(+)4-Propyl- 3,4,4a,5,6,10b-hexahydro-*2H*-naphtho[1,2-b][1,4]oxazin-9-ol binding (PET); **D2** |  |  | Decreased binding potential of an agonist | (Matuskey et al., 2016) | |
|  |  |  | [^11^C]-(+)4-Propyl- 3,4,4a,5,6,10b-hexahydro-*2H*-naphtho[1,2-b][1,4]oxazin-9-ol binding (Whole-brain voxel-wise analysis); **D2** |  |  | Decreased binding potential of an agonist | (Matuskey et al., 2016) | |
|  | Basal Ganglia – Striatum – Ventral Striatum | Human (18-73 years) | [^11^C]-(+)4-Propyl- 3,4,4a,5,6,10b-hexahydro-*2H*-naphtho[1,2-b][1,4]oxazin-9-ol and [^11^C]raclopride binding (PET); **D2; D3** |  |  | Decreased nondisplaceable binding potential by [^11^C]raclopride (antagonist);  No alteration with [^11^C]-(+)-PHNO (agonist) | (Nakajima et al., 2015) | |
|  |  | Human (19-55 years) | [^11^C]-(+)4-Propyl- 3,4,4a,5,6,10b-hexahydro-*2H*-naphtho[1,2-b][1,4]oxazin-9-ol binding (PET); **D2/3** |  |  | No alteration in binding potential of an agonist | (Matuskey et al., 2016) | |
|  | Basal Ganglia – Striatum - Caudate,  Putamen and Ventral striatum | Female Human (23-80 years) | [^18^F]fallypride binding (PET); **D2** |  |  | Decreased binding potential of an antagonist | (Dang et al., 2017) | |
|  |  | Human (18-81 years) | [^18^F]fallypride binding (PET); **D2; D3** |  |  | Decreased binding potential of an antagonist | (Dang et al., 2017) | |
|  | Basal Ganglia – Striatum – Nucleus accumbens | Male Fischer 344 rats (6 and 24 months) | [^3^H]spiperone binding (Autoradiography); **D2**  [^3^H]nemonapride binding (Autoradiography); **D2** | No alteration |  |  | (Araki et al., 1997) | |
|  |  | Male Fischer 344 x Brown-Norway rats (F1) (4 and 37 months) | [3H](+)-7-hydroxy-2-(*N, N*-di-*n*-propylamino)tetralin; **D3** | Increased |  |  | (Wallace and Booze, 1996) | |
|  | Basal Ganglia – Striatum – Olfactory tubercle | Fischer 344 rats (3 and 24 months) | [^35^S]-labelled mRNA hybridization and Northern blot; **D2** |  |  | Decreased D2 mRNA levels | (Weiss et al., 1992) | |
|  |  | Male Wistar-Kyoto rats (6 and 24 months) | RT-PCR (mRNA); **D2** |  |  | No alteration in D2 mRNA levels | (Valerio et al., 1994) | |
|  |  |  | RT-PCR (mRNA); **D3** |  |  | Decreased D3 mRNA levels | (Valerio et al., 1994) | |
|  | Basal Ganglia – Globus pallidus | Human (18-73 years) | [^11^C]-(+)4-Propyl- 3,4,4a,5,6,10b-hexahydro-*2H*-naphtho[1,2-b][1,4]oxazin-9-ol and [^11^C]raclopride binding (PET); **D2; D3** |  |  | No alteration in nondisplaceable binding potential  by [^11^C]raclopride (antagonist) and [^11^C]-(+)-PHNO (agonist) | (Nakajima et al., 2015) | |
|  | Basal Ganglia - Pallidum | Human (*postmortem*) (0-20 and >65 years years) | [^3^H]spiroperidol binding; **D2** | Decreased | No alteration |  | (Rinne, 1987) | |
|  |  | Human (19-55 years) | [^11^C]-(+)4-Propyl- 3,4,4a,5,6,10b-hexahydro-*2H*-naphtho[1,2-b][1,4]oxazin-9-ol binding (PET); **D2/3** |  |  | No alteration in binding potential of an agonist | (Matuskey et al., 2016) | |
|  | Basal Ganglia - Ventral Pallidum | Human (18-73 years) | [^11^C]-(+)4-Propyl- 3,4,4a,5,6,10b-hexahydro-*2H*-naphtho[1,2-b][1,4]oxazin-9-ol binding (PET); **D2; D3** |  |  | No alteration in nondisplaceable binding potential of an agonist | (Nakajima et al., 2015) | |
|  |  | Female Human  (23-80 years) | [^18^F]fallypride binding (PET); **D2** |  |  | Decreased binding potential of an antagonist | (Dang et al., 2017) |  |
|  |  | Human (18-81 years) | [^18^F]fallypride binding (PET); **D2; D3** |  |  | Decreased binding potential of an antagonist | (Dang et al., 2016) |  |
|  | Cerebral cortex - Frontal cortex | Male Fischer 344 rats (6 and 24 months) | [^3^H]spiperone binding (Autoradiography); **D2**  [^3^H]nemonapride binding (Autoradiography); **D2** | No alteration |  |  | (Araki et al., 1997) | |
|  |  | Male Wistar-Kyoto rats (6 and 24 months) | RT-PCR (mRNA); **D2** |  |  | Decreased D2 mRNA levels | (Valerio et al., 1994) | |
|  |  | New Zealand White Rabbits (5 mth & 5.5 yrs) | [^3^H]spiroperidol binding; **Dopamine receptors** | Decreased | No alteration |  | (Thal et al., 1980) | |
|  |  | Human (19-73 years) | [^11^C]3-N-methylspiperone binding (PET); **D2** | Decreased |  |  | (Wong et al., 1984) | |
|  |  | Human (21-82 years) | [^11^C]FLB 457 binding (PET; **D2** |  |  | Decreased binding potential of an antagonist | (Inoue et al., 2001) | |
|  |  | Human (19-74 years) | [^11^C]FLB 457 binding (PET); **D2; D3** |  |  | Decreased binding potential of an antagonist | (Kaasinen et al., 2000) | |
|  | Cerebral cortex - Parietal cortex | Male Fischer 344 rats (6 and 24 months) | [^3^H]spiperone binding (Autoradiography); **D2**  [^3^H]nemonapride binding (Autoradiography); **D2** | No alteration |  |  | (Araki et al., 1997) | |
|  |  | Human (21-82 years) | [^11^C]FLB 457 binding (PET); **D2** |  |  | Decreased binding potential of an antagonist | (Inoue et al., 2001) | |
|  | Cerebral cortex -Occipital cortex |  |  |  |  | Decreased binding potential of an antagonist | (Inoue et al., 2001) | |
|  | Cerebral cortex -Temporal cortex |  |  |  |  | Decreased binding potential of an antagonist | (Inoue et al., 2001) | |
|  | Cerebral cortex – Lateral and medial Temporal cortex | Human (19-74 years) | [^11^C]FLB 457 binding (PET); **D2; D3** |  |  | Decreased binding potential of an antagonist | (Kaasinen et al., 2000) | |
|  | Anterior Cingulate cortex |  |  |  |  | Decreased binding potential of an antagonist | (Kaasinen et al., 2000) | |
| Acetylcholine receptors | Cerebellum – Granular and molecular layer | Fischer 344 rats (6 and 22 months) | Silver grain (Autoradiography); **M2** | No alteration |  |  | (Tayebati et al., 2001) | |
|  |  |  | Silver grain (Autoradiography); **M4** | Increased |  |  | (Tayebati et al., 2001) | |
|  | Thalamus | Human (*postmortem*) (7-80 years) | [^3^H]-quinuclidinyl benzilate binding; **M2** | Increased |  |  | (Nordberg et al., 1992) | |
|  | Thalamus – Lateral and Medial Nuclear group | Male Wistar rats (5-6 and 24-25 months) | [^3^H]-quinuclidinyl benzilate binding (Autoradiography); **Acetylcholine receptors** | Decreased (lateral);  No alteration (medial) |  |  | (Blake et al., 1991) | |
|  | Hypothalamus |  |  | No alteration |  |  | (Blake et al., 1991) | |
|  | Hippocampus | Human (*postmortem*) (0-20 and >65 years years) | [^3^H]1quinuclidinyl(phenyl)-4-benzilate binding; **M2** | Decreased | No alteration |  | (Rinne, 1987) | |
|  | Hippocampus – Dentate gyrus | Male Wistar rats (5-6 and 24-25 months) | [^3^H]-quinuclidinyl benzilate binding (Autoradiography); **Acetylcholine receptors** | No alteration |  |  | (Blake et al., 1991) | |
|  | Hippocampus – Dentate gyrus – Granular and molecular layer | Male Fischer 344 rats (6 and 22 months) | [^3^H]N-methyl-scopolamine binding; **M2** | Decreased |  |  | (Tayebati et al., 2001) | |
|  |  |  | [^3^H]N-methyl-scopolamine binding; **M4** | No alteration |  |  | (Tayebati et al., 2001) | |
|  | Hippocampus – CornuAmmonis area 1 and 3 – Radial layer | Male Fischer 344 rats (6 and 22 months) | [^3^H]N-methyl-scopolamine binding; **M2** | Decreased |  |  | (Tayebati et al., 2001) | |
|  |  |  | [^3^H]N-methyl-scopolamine binding;**M4** | No alteration |  |  | (Tayebati et al., 2001) | |
|  | Hippocampus – CornuAmmonis area 1, 2 and 3 | Male Wistar rats (5-6 and 24-25 months) | [^3^H]-quinuclidinyl benzilate binding (Autoradiography); **Acetylcholine receptors**  [^35^S]-labeled mRNA hybridization; **M4** | No alteration |  | No alteration in M4 mRNA levels | (Blake et al., 1991) | |
|  | Amygdala |  |  | No alteration |  | No alteration in M4 mRNA levels | (Blake et al., 1991) | |
|  | Basal Ganglia - Striatum | Fischer 344 rats (6 or 15 and 22 months) | [^3^H]N-methyl-scopolamine binding; **M2** | No alteration |  |  | (Tayebati et al., 2004) | |
|  |  |  | [^3^H]N-methyl-scopolamine binding; **M4** | Decreased |  |  | (Tayebati et al., 2004) | |
|  | Basal Ganglia - Neostriatum | Male Fischer 344 x Brown-Norway rats (3 and 33 months) | Northern Blot (mRNA); **M2** |  |  | No alteration in M2 mRNA levels | (Lee et al., 1994) | |
|  |  |  | Northern Blot (mRNA); **M4** |  |  | Decreased M4 mRNA levels | (Lee et al., 1994) | |
|  | Basal Ganglia – Striatum – Dorsolateral and dorsomedial striatum | Male Fischer 344 x Brown-Norway rats (F1) (6, 18 and 24 months) | Oxotremorine-M stimulated [^35^S]GTPγS binding (Autoradiography); **M2/M4** [^3^H]AFDX-384 binding (Autoradiography); **M2/M4** | No alteration |  | Decreased agonist (oxotremorine-M) stimulated [^35^S]GTPγS binding (dorsomedial striatum); No alteration (dorsolateral striatum) | (Nieves-Martinez et al., 2012) | |
|  | Basal Ganglia – Striatum – Caudate and Putamen | Male Wistar rats (5-6 and 24-25 months) | [^3^H]-quinuclidinyl benzilate binding (Autoradiography); **Acetylcholine receptors**  [^35^S]-labeled hybridization (mRNA); **M4** | Decreased | No alteration | No alteration in M4 mRNA levels | (Blake et al., 1991) | |
|  |  | Human (*postmortem*) (0-20 and >65 years years) | [^3^H]1-quinuclidinyl(phenyl)-4-benzilate binding; **M2** | Decreased | No alteration |  | (Rinne, 1987) | |
|  | Basal Ganglia – Striatum – Nucleus accumbens | Male Wistar rats (5-6 and 24-25 months) | [^3^H]-quinuclidinyl benzilate binding (Autoradiography); **Acetylcholine receptors**  [^35^S]-labeled hybridization (mRNA); **M4** | Decreased |  | No alteration in M4 mRNA levels | (Blake et al., 1991) | |
|  | Basal Ganglia – Striatum – Olfactory tubercle |  |  | Decreased |  | No alteration in M4 mRNA levels | (Blake et al., 1991) | |
|  | Rhinencephalon – Piriform cortex |  |  | Decreased |  | No alteration in M4 mRNA levels | (Blake et al., 1991) | |
|  | Cerebral cortex - Frontal cortex |  |  | Decreased |  | No alteration in M4 mRNA levels | (Blake et al., 1991) | |
|  |  | Human (*postmortem*) (0-20 and >65 years years) | [^3^H]1-quinuclidinyl(phenyl)-4-benzilate binding; **M2** | Decreased | No alteration |  | (Rinne, 1987) | |
|  |  | Human (*postmortem*) (7-80 years) | [^3^H]-quinuclidinyl benzilate binding; **M2** | Decreased |  |  | (Nordberg et al., 1992) | |
|  | Cingulate cortex | Male Wistar rats (5-6 and 24-25 months) | [^3^H]-quinuclidinyl benzilate binding (Autoradiography); **Acetylcholine receptors**  [^35^S]-labeled hybridization (mRNA); **M4** | Decreased |  | No alteration in M4 mRNA levels | (Blake et al., 1991) | |
|  | Cingulate cortex – Retrosplenial cortex |  |  | Decreased |  | No alteration in M4 mRNA levels | (Blake et al., 1991) | |
|  | Parieto-frontal cortex |  |  | No alteration |  | No alteration in M4 mRNA levels | (Blake et al., 1991) | |
|  | Reunions nucleus |  |  | No alteration |  |  | (Blake et al., 1991) | |
| Serotonin receptors | Pons - Locus Coeruleus | Human (*postmortem*) (15-81 years) | [^3^H]8-hydroxy-2-(di*-N*-propylamino)tetralin binding (Autoradiography); **5-HT1A** | Decreased (men);  No alteration (women) |  |  | (Dillon et al., 1991) | |
|  | Cerebellum – Granular and molecular layer | ICR Mouse (3 and 12 months) | Immunocytochemistry;  **5-HT1A** | Decreased |  |  | (Yew et al., 2009) | |
|  |  | Human (57-78 and 82-91 years) |  | Decreased |  |  | (Yew et al., 2009) | |
|  | Dorsal and median Raphe nucleus | Male Syrian Hamsters (Harlan Sprague Dawley, SYR- HSD) (3-4 and 17-19 months) | [^3^H]4-(2´-Methoxy)-phenyl-1-[2´-(N-2”-pyridinyl)-p-fluorobenzamido]ethyl-piperzin binding (Autoradiography); **5-HT1A**  8-OH-DPAT stimulated [^35^S]GTPγS binding (Autoradiography); **5-HT1A** | No alteration |  | No alteration in agonist (8-OH-DPAT) stimulated [^35^S]GTPγS binding | (Duncan and Hensler, 2002) | |
|  | Raphe nucleus | Human (22-53 years) | *Carbonyl*-[^11^C]-N-[2-[4-(2-methoxyphenyl)-1-piper- azinyl]ethyl]-N-(2-pyridinyl)cyclohexane carboxamide binding (PET); **5-HT1A** |  |  | No alteration in binding potential of an antagonist | (Tauscher et al., 2001) | |
|  |  | Human (*postmortem*) (15-81 years) | [^3^H]8-hydroxy-2-(di*-N*-propylamino)tetralin binding (Autoradiography);  **5-HT1A** | Decreased (men’s dorsal);  No alteration (men’s median and obscurus; women’s brain) |  |  | (Dillon et al., 1991) | |
|  | Dorsal Raphe | Human (24-56 years) | *Carbonyl*-[^11^C]-N-[2-[4-(2-methoxyphenyl)-1-piper- azinyl]ethyl]-N-(2-pyridinyl)cyclohexane carboxamide binding (PET); **5-HT1A** |  |  | No alteration in binding potential of an antagonist | (Parsey et al., 2002) | |
|  | Thalamus | Human (21-80 years) |  |  |  | No alteration in binding potential of an antagonist | (Meltzer et al., 2001) | |
|  |  | Human (18-61 years) | [^11^C]P943 binding (PET); **5-HT1B** |  |  | No alteration in binding potential of an antagonist | (Matuskey et al., 2012) | |
|  | Thalamus - Medial nuclear group | Human (*postmortem*) (15-81 years) | [^3^H]8-hydroxy-2-(di*-N*-propylamino)tetralin binding (Autoradiography); **5-HT1A** | No alteration (men and women) |  |  | (Dillon et al., 1991) | |
|  | Hypothalamus | Human (18-61 years) | [^11^C]P943 binding (PET); **5-HT1B** |  |  | Decreased binding potential of an antagonist | (Matuskey et al., 2012) | |
|  | White matter | Human (*postmortem*) (15-81 years) | [^3^H]8-hydroxy-2-(di*-N*-propylamino)tetralin binding (Autoradiography); **5-HT1A** | No alteration (men and women) |  |  | (Dillon et al., 1991) | |
|  | Hippocampus | Human (24-56 years) | *Carbonyl*-[^11^C]-N-[2-[4-(2-methoxyphenyl)-1-piper- azinyl]ethyl]-N-(2-pyridinyl)cyclohexane carboxamide binding (PET); **5-HT1A** |  |  | No alteration in binding potential of an antagonist | (Parsey et al., 2002) | |
|  |  | Human (21-80 years) |  |  |  | Decreased binding potential of an antagonist (men); No alteration (women) | (Meltzer et al., 2001) | |
|  |  | Human (18-61 years) | [^11^C]P943 binding (PET); **5-HT1B** |  |  | No alteration in binding potential of an antagonist | (Matuskey et al., 2012) | |
|  |  | Human (*postmortem*) (30-83 years) | [^3^H]8-hydroxy-2-(di*-N*-propylamino)tetralin binding; **5-HT1A** | No alteration (men);  Decreased (women) | No alteration |  | (Palego et al., 1997) | |
|  | Hippocampus – Dentate gyrus | Male Syrian Hamsters (Harlan Sprague Dawley, SYR- HSD) (3-4 and 17-19 months) | [^3^H]4-(2´-Methoxy)-phenyl-1-  [2´-(N-2”-pyridinyl)-p-fluorobenzamido]ethyl-piperzin binding (Autoradiography); **5-HT1A**  8-OH-DPAT stimulated [^35^S]GTPγS binding (Autoradiography); **5-HT1A** | Decreased |  | No alteration in agonist (8-OH-DPAT) stimulated [^35^S]GTPγS binding | (Duncan and Hensler, 2002) | |
|  |  | Human *(postmortem)* (45-84 years) | [^3^H]8-hydroxy-2-(di*-N*-propylamino)tetralin binding (Autoradiography); **5-HT1A**  RT-PCR mRNA; **5-HT1A** | Decreased |  | No alteration in 5-HT1A mRNA levels | (Burnet et al., 1994) | |
|  | Hippocampus – Dentate gyrus – Granular layer, Molecular layer and hilus | Human (*postmortem*) (15-81 years) | [^3^H]8-hydroxy-2-(di*-N*-propylamino)tetralin binding (Autoradiography); **5-HT1A** | Decreased (men); Increased (women’s granular layer); No alteration (women’s molecular layer and hilus) |  |  | (Dillon et al., 1991) | |
|  | Hippocampus – CornuAmmonis area – Pyramidal and molecular layer | Human (*postmortem*) (15-81 years) | [^3^H]8-hydroxy-2-(di*-N*-propylamino)tetralin binding (Autoradiography); **5-HT1A** | No alteration (men and women) |  |  | (Dillon et al., 1991) | |
|  | Hippocampus – CornuAmmonis area 1 | Male Syrian Hamsters (Harlan Sprague Dawley, SYR- HSD) (3-4 and 17-19 months) | [^3^H]4-(2´-Methoxy)-phenyl-1-  [2´-(N-2”-pyridinyl)-p-fluorobenzamido]ethyl-piperzin binding (Autoradiography); **5-HT1A**  8-OH-DPAT stimulated [^35^S]GTPγS binding (Autoradiography); **5-HT1A** | No alteration |  | Increased agonist (8-OH-DPAT) stimulated [^35^S]GTPγS binding | (Duncan and Hensler, 2002) | |
|  |  | Human *(postmortem)* (45-84 years) | [^3^H]8-hydroxy-2-(di*-N*-propylamino)tetralin binding (Autoradiography); **5-HT1A**  RT-PCR (mRNA); **5-HT1A** | Decreased |  | No alteration in 5-HT1A mRNA levels | (Burnet et al., 1994) | |
|  | Hippocampus –Subiculum | Human (*postmortem*) (15-81 years) | [^3^H]8-hydroxy-2-(di*-N*-propylamino)tetralin binding (Autoradiography); **5-HT1A** | Decreased (men);  No alteration (women) |  |  | (Dillon et al., 1991) | |
|  | Parahippocampal gyrus | Human *(postmortem)* (45-84 years) | [^3^H]8-hydroxy-2-(di*-N*-propylamino)tetralin binding (Autoradiography); **5-HT1A**  RT-PCR (mRNA); **5-HT1A** | Decreased |  | No alteration in 5-HT1A mRNA levels | (Burnet et al., 1994) | |
|  |  | Human (*postmortem*) (15-81 years) | [^3^H]8-hydroxy-2-(di*-N*-propylamino)tetralin binding (Autoradiography); **5-HT1A** | Decreased (men’s external band);  No alteration (women and in men’s middle and internal band) |  |  | (Dillon et al., 1991) | |
|  | Amygdala | Human (24-56 years) | *Carbonyl*-[^11^C]-N-[2-[4-(2-methoxyphenyl)-1-piper- azinyl]ethyl]-N-(2-pyridinyl)cyclohexane carboxamide binding (PET); **5-HT1A** |  |  | No alteration in binding potential of an antagonist | (Parsey et al., 2002) | |
|  |  | Human (18-61 years) | [^11^C]P943 binding (PET); **5-HT1B** |  |  | No alteration in binding potential of an antagonist | (Matuskey et al., 2012) | |
|  | Claustrum | Human (*postmortem*) (15-81 years) | [^3^H]8-hydroxy-2-(di*-N*-propylamino)tetralin binding (Autoradiography); **5-HT1A** | No alteration (men and women) |  |  | (Dillon et al., 1991) | |
|  | Basal Ganglia | Human (21-80 years) | *Carbonyl*-[^11^C]-N-[2-[4-(2-methoxyphenyl)-1-piper- azinyl]ethyl]-N-(2-pyridinyl)cyclohexane carboxamide binding (PET); **5-HT1A** |  |  | No alteration in binding potential of an antagonist | (Meltzer et al., 2001) | |
|  | Basal Ganglia – Striatum – Caudate and Putamen | Human (18-61 years) | [^11^C]P943 binding (PET); **5-HT1B** |  |  | Increased binding potential of an antagonist (putamen);  No alteration (caudate) | (Matuskey et al., 2012) | |
|  | Basal Ganglia -  Pallidum |  |  |  |  | Increased binding potential of an antagonist | (Matuskey et al., 2012) | |
|  | Cerebral cortex | Male Wistar rats (4 and 24 months) | [^3^H]8-hydroxy-2-(di*-N*-propylamino)tetralin binding; **5-HT1A** | Decreased | No alteration |  | (Huguet et al., 1994) | |
|  | Cerebral cortex - Frontal cortex | Human *(postmortem)* (16-75 years) |  | No alteration | No alteration |  | (Arranz et al., 1993) | |
|  |  |  | [^3^H]5-hydroxytryptamine binding; **5-HT1D** | Decreased | No alteration |  | (Arranz et al., 1993) | |
|  |  | Human (18-61 years) | [^11^C]P943 binding (PET); **5-HT1B** |  |  | Decreased binding potential of an antagonist | (Matuskey et al., 2012) | |
|  | Cerebral cortex - Frontal cortex – Superior frontal gyrus | Human (*postmortem*) (15-81 years) | [^3^H]8-hydroxy-2-(di*-N*-propylamino)tetralin binding (Autoradiography); **5-HT1A** | Decreased (men’s prefrontal, and external and internal band of frontal parietal level);  No alteration (men’s middle band of frontal parietal level);  No alteration (women) |  |  | (Dillon et al., 1991) | |
|  | Cerebral cortex - Frontal cortex – Middle and inferior frontal gyrus | Human (*postmortem*) (15-81 years) |  | No alteration (men and women) |  |  | (Dillon et al., 1991) | |
|  | Cerebral cortex - Frontal cortex – Orbitofrontal cortex | Human (22-53 years) | *Carbonyl*-[^11^C]-N-[2-[4-(2-methoxyphenyl)-1-piper- azinyl]ethyl]-N-(2-pyridinyl)cyclohexane carboxamide binding (PET); **5-HT1A** |  |  | Decreased binding potential of an antagonist | (Tauscher et al., 2001) | |
|  | Cerebral cortex - Frontal cortex – Lateral Orbitofrontal cortex | Human (21-80 years) |  |  |  | Decreased binding potential of an antagonist (men); No alteration (women) | (Meltzer et al., 2001) | |
|  | Cerebral cortex - Frontal cortex – Precentral gyrus | Human (*postmortem*) (15-81 years) | [^3^H]8-hydroxy-2-(di*-N*-propylamino)tetralin binding (Autoradiography) **5-HT1A** | Decreased (men);  No alteration (women) |  |  | (Dillon et al., 1991) | |
|  | Cerebral cortex - Frontal cortex – Prefrontal cortex | Rhesus monkeys *(Macaca mulatta)* (3-10 years and >20 years) | [^3^H]5-hydroxytrytamine binding (Autoradiography); **Serotonin receptors** | No alteration | No alteration |  | (Bigham and Lidow, 1995) | |
|  |  | Human *(postmortem)* (1-88 years) | 8-OH-DPAT stimulated [^35^S]GTPγS binding; **5-HT1A** |  |  | Decreased agonist (8-OH-DPAT) stimulated [^35^S]GTPγS binding | (Araki et al., 1997) | |
|  |  | Human (*postmortem*) | [^3^H]8-hydroxy-2-(di*-N*-propylamino)tetralin binding; **5-HT1A** | No alteration | No alteration |  | (Palego et al., 1997) | |
|  | Cerebral cortex - Frontal cortex – Dorsolateral prefrontal cortex | Human (22-53 years) | *Carbonyl*-[^11^C]-N-[2-[4-(2-methoxyphenyl)-1-piper- azinyl]ethyl]-N-(2-pyridinyl)cyclohexane carboxamide binding (PET); **5-HT1A** |  |  | Decreased binding potential of an antagonist | (Tauscher et al., 2001) | |
|  | Cerebral cortex - Frontal cortex – Medial prefrontal cortex | Human (24-56 years) |  |  |  | No alteration in binding potential of an antagonist | (Parsey et al., 2002) | |
|  | Cerebral cortex - Frontal cortex – Orbital prefrontal cortex |  |  |  |  | No alteration in binding potential of an antagonist |  |  |
|  | Cerebral cortex - Frontal cortex – Primary motor cortex | Rhesus monkeys *(Macaca mulatta)* (3-10 years and >20 years) | [^3^H]5-hydroxytrytamine binding (Autoradiography); **Serotonin receptors** | No alteration | Decreased (I-IIIa);  No alteration (IIIb-VI) |  | (Bigham and Lidow, 1995) | |
|  | Cerebral cortex - Parietal cortex | Human (22-53 years) | *Carbonyl*-[^11^C]-N-[2-[4-(2-methoxyphenyl)-1-piper- azinyl]ethyl]-N-(2-pyridinyl)cyclohexane carboxamide binding (PET); **5-HT1A** |  |  | Decreased binding potential of an antagonist | (Tauscher et al., 2001) | |
|  |  | Human (18-61 years) | [^11^C]P943 binding (PET); **5-HT1B** |  |  | Decreased binding potential of an antagonist | (Matuskey et al., 2012) | |
|  |  | Human (*postmortem*) (30-83 years) | [^3^H]8-hydroxy-2-(di*-N*-propylamino)tetralin binding; **5-HT1A** | Unalterated in men; Decreased in women | No alteration |  | (Palego et al., 1997) | |
|  | Cerebral cortex - Parietal cortex – Somatosensory cortex | Rhesus monkeys *(Macaca mulatta)* (3-10 years and >20 years) | [^3^H]5-hydroxytrytamine binding (Autoradiography); **Serotonin receptors** | Decreased (Layer I-V);  No alteration (Layer VI) | Decreased (I-V); No alteration (VI) |  | (Bigham and Lidow, 1995) | |
|  | Cerebral cortex - Parietal cortex – Inferior parietal lobe | Human (*postmortem*) (15-81 years) | [^3^H]8-hydroxy-2-(di*-N*-propylamino)tetralin binding (Autoradiography); **5-HT1A** | Decreased (men);  No alteration (women) |  |  | (Dillon et al., 1991) | |
|  | Cerebral cortex - Parietal cortex – Postcentral gyrus |  |  | Decreased (men);  No alteration (women) |  |  |  |  |
|  | Cerebral cortex -Occipital cortex | Human (22-53 years) | *Carbonyl*-[^11^C]-N-[2-[4-(2-methoxyphenyl)-1-piper- azinyl]ethyl]-N-(2-pyridinyl)cyclohexane carboxamide binding (PET); **5-HT1A** |  |  | Decreased binding potential of an antagonist | (Tauscher et al., 2001) | |
|  |  | Human (21-80 years) |  |  |  | Decreased binding potential of an antagonist (men); No alteration (women) | (Meltzer et al., 2001) | |
|  |  | Human (18-61 years) | [^11^C]P943 binding (PET); **5-HT1B** |  |  | Decreased binding potential of an antagonist | (Matuskey et al., 2012) | |
|  |  | Human (*postmortem*) (30-83 years) | [^3^H]8-hydroxy-2-(di*-N*-propylamino)tetralin binding; **5-HT1A** | No alteration (men);  Increased (women) | Increased (men);  No alteration (women) |  | (Palego et al., 1997) | |
|  | Cerebral cortex -Occipital cortex – Primary visual cortex | Rhesus monkeys *(Macaca mulatta)* (3-10 years and >20 years) | [^3^H]5-hydroxytrytamine binding (Autoradiography); **Serotonin receptors** | No alteration | No alteration |  | (Bigham and Lidow, 1995) | |
|  | Cerebral cortex -Temporal cortex | Human (18-61 years) | [^11^C]P943 binding (PET); **5-HT1B** |  |  | Decreased binding potential of an antagonist | (Matuskey et al., 2012) | |
|  |  | Human (*postmortem*) (30-83 years) | [^3^H]8-hydroxy-2-(di*-N*-propylamino)tetralin binding; **5-HT1A** | No alteration | No alteration |  | (Palego et al., 1997) | |
|  | Cerebral cortex -Temporal cortex – Transverse temporal gyrus | Human (*postmortem*) (15-81 years) |  | Decreased (men’s middle and internal band);  No alteration (women; men’s external band) |  |  | (Dillon et al., 1991) | |
|  | Cerebral cortex -Temporal cortex – Superior/Medial/Inferior temporal gyrus | Human (*postmortem*) (15-81 years) | [^3^H]8-hydroxy-2-(di*-N*-propylamino)tetralin bind (Autoradiography); **5-HT1A** | Decreased (men); No alteration (women) |  |  | (Dillon et al., 1991) | |
|  | Cerebral cortex -Temporal cortex – Mediotemporal cortex | Human (22-53 years) | *Carbonyl*-[^11^C]-N-[2-[4-(2-methoxyphenyl)-1-piper- azinyl]ethyl]-N-(2-pyridinyl)cyclohexane carboxamide binding (PET); **5-HT1A** |  |  | No alteration in binding potential of an antagonist | (Tauscher et al., 2001) | |
|  | Cerebral cortex -Temporal cortex – Mesial temporal cortex | Human (21-80 years) |  |  |  | Decreased binding potential of an antagonist (men); No alteration (women) | (Meltzer et al., 2001) | |
|  | Cerebral cortex -Temporal cortex – Lateral temporal cortex | Human (22-53 years) |  |  |  | Decreased binding potencial of an antagonist | (Tauscher et al., 2001) | |
|  | Cerebral cortex - Cingulate body | Human (24-56 years) |  |  |  | No alteration in binding potential of an antagonist | (Parsey et al., 2002) | |
|  | Cingulate cortex | Human (*postmortem*) (15-81 years) | [^3^H]8-hydroxy-2-(di*-N*-propylamino)tetralin binding (Autoradiography); **5-HT1A** | No alteration (men’s pre-frontal cortex);  Decreased (men’s frontal parietal);  No alteration (women) |  |  | (Dillon et al., 1991) | |
|  | Anterior Cingulate cortex | Human (22-53 years) | *Carbonyl*-[^11^C]-N-[2-[4-(2-methoxyphenyl)-1-piper- azinyl]ethyl]-N-(2-pyridinyl)cyclohexane carboxamide binding (PET); **5-HT1A** |  |  | Decreased binding potential of an antagonist | (Tauscher et al., 2001) | |
|  |  | Human (24-56 years) |  |  |  | No alteration in binding potential of an antagonist | (Parsey et al., 2002) | |
|  | Cingulate cortex – Subgenual Cingulate | Human (21-80 years) |  |  |  | Decreased binding potential of an antagonist (men); No alteration (women) | (Meltzer et al., 2001) | |
|  | Cingulate cortex – Pregenual Cingulate |  |  |  |  | Decreased binding potential of an antagonist (men); No alteration (women) |  |  |
|  | Insular cortex | Human (*postmortem*) (15-81 years) | [^3^H]8-hydroxy-2-(di*-N*-propylamino)tetralin binding (Autoradiography); **5-HT1A** | Decreased (men);  No alteration (women) |  |  | (Dillon et al., 1991) | |
|  | Brainstem - Brainstrem raphe | Human (21-80 years) | *Carbonyl*-[^11^C]-N-[2-[4-(2-methoxyphenyl)-1-piper- azinyl]ethyl]-N-(2-pyridinyl)cyclohexane carboxamide binding (PET); **5-HT1A** |  |  | Decreased binding potential of an antagonist (men); No alteration (women) | (Meltzer et al., 2001) | |
|  | Lateral Occipitotemporal gyrus | Human (*postmortem*) (15-81 years) | [^3^H]8-hydroxy-2-(di*-N*-propylamino)tetralin binding (Autoradiography); **5-HT1A** | Decreased (men’s external and internal band);  No alteration (men’s middle band);  No alteration (women) |  |  | (Dillon et al., 1991) | |
|  | Orbital gyrus |  |  | No alteration (men and women) |  |  |  |  |
|  | Gyrus rectus |  |  |  |  |  |  |  |
| Purinoceptors | ------------------------------------ | | | | | | | |
| Opioid Receptors | Mindbrain (Mesencephalon) | Male Fischer 344 rats (5 and 26 months) | [^3^H]dihydromorphine binding; **Opioid receptors** | No alteration | No alteration |  | (Messing et al., 1981) | |
|  |  | Male Sprague-Dawley rats (2 and 19 months) | ^3^H-bremazocine binding; **κ** | No alteration | No alteration |  | (Maggi et al., 1989) | |
|  | Substantia nigra | Male Hartley Guinea-pigs (1 and 36 months) | [^3^H]bremazocine binding (Autoradiography); **κ** | Decreased |  |  | (Hiller et al., 1992) | |
|  | Thalamus | Male Fischer 344 rats (5 and 26 months) | [^3^H]dihydromorphine binding; **Opioid receptors** | No alteration | No alteration |  | (Messing et al., 1981) | |
|  |  | Male Sprague-Dawley rats (2 and 19 months) | ^3^H-bremazocine binding; **κ** | Increased | No alteration |  | (Maggi et al., 1989) | |
|  | Hypothalamus | Male Sprague-Dawley rats (2 and 19 months) | ^3^H-bremazocine binding; **κ** | No alteration | No alteration |  | (Maggi et al., 1989) | |
|  |  | Male Sprague-Dawley rats (2 and 22 months) | ^3^H-dihydromorphine binding; **µ** | Decreased | No alteration |  | (Piva et al., 1987) | |
|  | Hippocampus | Male Wistar rats (2-3, 6-12 and 24 months) | ^3^H-etorphine binding; **Opioid receptors** | Decreased | No alteration |  | (Hess et al., 1981) | |
|  |  | Male Sprague-Dawley rats (2 and 19 months) | ^3^H-bremazocine binding; **κ** | No alteration | No alteration |  | (Maggi et al., 1989) | |
|  | Amygdala | Male Fischer 344 rats (5 and 26 months) | [^3^H]dihydromorphine binding; **Opioid receptors** | No alteration | No alteration |  | (Messing et al., 1981) | |
|  |  | Male Wistar rats (2-3, 6-12 and 24 months) | ^3^H-etorphine binding; **Opioid receptors** | No alteration | No alteration |  | (Hess et al., 1981) | |
|  |  | Male Sprague-Dawley rats (2 and 19 months) | ^3^H-bremazocine binding; **κ** | Increased | No alteration |  | (Maggi et al., 1989) | |
|  | Basal Ganglia - Striatum | Male Fischer 344 rats (5 and 26 months) | [^3^H]dihydromorphine binding; **Opioid receptors** | Decreased | No alteration |  | (Messing et al., 1981) | |
|  |  | Male Wistar rats (2-3, 6-12 and 24 months) | ^3^H-etorphine binding; **Opioid receptors** | Decreased | No alteration |  | (Hess et al., 1981) | |
|  |  | Male Sprague-Dawley rats (2 and 19 months) | ^3^H-bremazocine binding; **κ** | No alteration | No alteration |  | (Maggi et al., 1989) | |
|  | Basal Ganglia – Striatum – Caudate and Putamen | Male Hartley Guinea-pigs (1 and 36 months) | [^3^H]bremazocine binding (Autoradiography); **κ** | Decreased |  |  | (Hiller et al., 1992) | |
|  | Basal Ganglia – Globus Pallidus | Male Hartley Guinea-pigs (1 and 36 months) | [^3^H]bremazocine binding (Autoradiography); **κ** | No alteration |  |  | (Hiller et al., 1992) | |
|  | Cerebral cortex - Frontal cortex – Prefrontal cortex | Human *(postmortem)* (1-88 years) | DAMGO stimulated [^35^S]GTPγS binding; **µ** |  |  | Increased agonist (DAMGO) stimulated [^35^S]GTPγS binding | (González-Maeso et al., 2002) | |
|  | Cerebral cortex - Frontal cortex - Lateral Agranular Field | Male Hartley Guinea-pigs (1 and 36 months) | [^3^H]bremazocine binding (Autoradiography); **κ** | Decreased |  |  | (Hiller et al., 1992) | |
|  | Cerebral cortex – Parietal cortex – Primary and Supplementary Somatosensory cortex | Male Hartley Guinea-pigs (1 and 36 months) | [^3^H]bremazocine binding (Autoradiography); **κ** | Decreased |  |  | (Hiller et al., 1992) | |
|  | Cerebral cortex -Occipital cortex |  |  | Decreased |  |  |  |  |
|  | Cerebral cortex -Temporal cortex |  |  | Decreased |  |  |  |  |
|  | Cerebral cortex -Anterior cortex | Male Fischer 344 rats (5 and 26 months) | [^3^H]dihydromorphine binding; **Opioid receptors** | Decreased | No alteration |  | (Messing et al., 1981) | |
|  |  | Male Sprague-Dawley rats (2 and 19 months) | ^3^H-bremazocine binding; **κ** | No alteration | No alteration |  | (Maggi et al., 1989) | |
|  |  | Male Wistar rats (2-3, 6-12 and 24 months) | ^3^H-etorphine binding; **Opioid receptors** | No alteration | No alteration |  | (Hess et al., 1981) | |
|  | Cerebral cortex - Posterior cortex | Male Sprague-Dawley rats (2 and 19 months) | ^3^H-bremazocine binding; **κ** | No alteration | No alteration |  | (Maggi et al., 1989) | |
|  | Cerebral cortex – Frontal Lobe - Frontal poles | Male Fischer 344 rats (5 and 26 months) | [^3^H]dihydromorphine binding; **Opioid receptors** | Decreased | Increased |  | (Messing et al., 1981) | |
|  |  | Male Wistar rats (2-3, 6-12 and 24 months) | ^3^H-etorphine binding; **Opioid receptors** | Decreased | No alteration |  | (Hiller et al., 1992) | |
|  |  | Male Sprague-Dawley rats (2 and 19 months) | ^3^H-bremazocine binding; **κ** | No alteration | No alteration |  | (Maggi et al., 1989) | |
|  | Medial prefrontal cortex | Male Wistar rats (60–150 days) | nor-binaltorphimine stimulated [^35^S] GTPγS binding; **κ** |  |  | Decreased antagonist (nor-binaltorphimine) stimulated [^35^S]GTPγS binding | (Sirohi and Walker, 2015) | |
|  | Cingulate cortex - Retrosplenial cortex | Male Hartley Guinea-pigs (1 and 36 months) | [^3^H]bremazocine binding (Autoradiography); **κ** | No alteration |  |  | (Hiller et al., 1992) | |
|  | Agranular Insular cortex – Dorsal part |  |  | Decreased |  |  |  |  |
| Somatostatin Receptors | Cerebellum – Granular and molecular layer | Human (*postmortem*) (28-86 years) | [^125^I-Try^0^, DTrp^8^]S14 binding, Autoradiography; **Somatostatin receptors** | No alteration |  |  | (Laquerriere et al., 1994) | |
|  | Hypothalamus | Male Wistar rats (*Rattus norvegicus)* (1 and 18 months) | ^125^I-TyrI-somatostatin binding; **Somatostatin receptors** |  | No alteration |  | (Sirvio et al., 1987) | |
|  | Pituitary gland | Male Fischer 344 rats (6 and 24 months) | RT-PCR (mRNA); **SSTR2; SSTR5** |  |  | Decreased SSTR2 and SSTR5 mRNA levels | (Shimokawa et al., 2000) | |
|  |  | Sprague-Dawley rats (2 days and 1 year) | cRNA labeled with [^32^P]-UTP (mRNA); **SSTR2** |  |  | Increased SSTR2 mRNA levels | (Reed et al., 1999) | |
|  |  |  | cRNA labeled with [^32^P]-UTP ( qPCR - mRNA); **SSTR1; SSTR3; SSTR4; SSTR5** |  |  | No alteration in SSTR1, SSTR3, SSTR4, SSTR5 mRNA levels | (Villar-Cheda et al., 2012) | |
|  | Hippocampus | Male Wistar rats (*Rattus norvegicus)* (1 and 18 months) | ^125^I-TyrI-somatostatin binding; **Somatostatin receptors** |  | Decreased |  | (Sirvio et al., 1987) | |
|  | Basal Ganglia - Striatum |  |  |  | Decreased |  |  |  |
|  | Cerebral cortex - Frontal cortex |  |  |  | Decreased |  |  |  |
| Angiotensin Receptors | Substantia nigra | Male Sprague-Dawley rats (10 weeks and 24 months) | qPCR (mRNA); **AT1**  Western blot; **AT1** | Increased |  | Increased AT1 mRNA levels | (Villar-Cheda et al., 2012) | |
|  |  | Male Sprague-Dawley rats (10 weeks & 18-20 months) | qPCR (mRNA); **AT1**  Western Blot; **AT1** | Increased |  | Increased AT1 mRNA levels | (Villar-Cheda et al., 2014) | |
|  | Striatum | Male Sprague-Dawley rats (10 weeks & 18-20 months) | qPCR (mRNA); **AT1**  Western Blot; **AT1** | Increased |  | Increased AT1 mRNA levels | (Villar-Cheda et al., 2014) | |
| Cannabinoid Receptors | Cerebellum | Human (18-45 and 45-70 years) | N-[2-(3-cyano-phenyl)-3-(4-(2-[^18^F]fluor-  ethoxy)phenyl)-1-methylpropyl]-2-(5-methyl-2-pyridyloxy)-2- methylproponamide binding (PET); **CB1** |  | Increased (women);  No alteration (men) |  | (Van Laere et al., 2008) | |
|  | Substantia nigra | Male Wistar rats (3 months and >2 years) | [^3^H]-WIN 55,212-2 binding (Autoradiography); **Cannabinoid receptors**  WIN 55,212-2 stimulated [^35^S]GTPγS binding (Autoradiography); **Cannabinoid receptors** | Decreased (pars reticulata) |  | Decreased agonist (WIN 55,212-2) stimulated [^35^S]GTPγS binding | (Romero et al., 1998) | |
|  | Thalamus | Human (18-45 and 45-70 years) | N-[2-(3-cyano-phenyl)-3-(4-(2-[^18^F]fluor-  ethoxy)phenyl)-1-methylpropyl]-2-(5-methyl-2-pyridyloxy)-2- methylproponamide binding (PET); **CB1** |  | Increased (women);  No alteration (men) |  | (Van Laere et al., 2008) | |
|  | Hippocampus | Male Wistar rats (2 and 24 months) | Western Blot; **CB1** | Decreased |  |  | (Canas et al., 2009) | |
|  |  | Human (18-45 and 45-70 years) | N-[2-(3-cyano-phenyl)-3-(4-(2-[^18^F]fluor-  ethoxy)phenyl)-1-methylpropyl]-2-(5-methyl-2-pyridyloxy)-2- methylproponamide binding (PET); **CB1** |  | Increased (women);  No alteration (men) |  | (Van Laere et al., 2008) | |
|  |  | Mice (6 and 15 months) | [^18^F]FMPEP-*d_2_* binding (PET); **CB1** |  | Increased |  | (Takkinen et al., 2018) | |
|  | Hippocampus – Dentate gyrus | Male Wistar rats (3 months and >2 years) | [^3^H]-WIN 55,212-2 binding (Autoradiography); **Cannabinoid receptors** | Increased |  |  | (Romero et al., 1998) | |
|  | Hippocampus – CornuAmmonis area 1 |  |  | No alteration |  |  |  |  |
|  | Amygdala | Human (18-45 and 45-70 years) | N-[2-(3-cyano-phenyl)-3-(4-(2-[^18^F]fluor-  ethoxy)phenyl)-1-methylpropyl]-2-(5-methyl-2-pyridyloxy)-2- methylproponamide binding (PET); **CB1** |  | Increased (women);  No alteration (men) |  | (Van Laere et al., 2008) | |
|  | Basal Ganglia – Striatum – Caudate and Putamen | Male Wistar rats (3 and 24 months) | [^3^H]CP55,940 binding (Radioautography); **Cannabinoid receptors**  α-[^35^S]dATP radiolabed hybridization (mRNA); **Cannabinoid receptors** | Decreased |  | Decreased cannabinoid receptors mRNA levels | (Mailleux and Vanderhaeghen, 1992) | |
|  |  | Male Wistar rats (3 months and >2 years) | [^3^H]-WIN 55,212-2 binding (Autoradiography); **Cannabinoid receptors**  [^3^H]-WIN 55,212-2 stimulated [^35^S]GTPγS binding (Autoradiography); **Cannabinoid receptors**  [^35^S]dATP labelled hybridization (mRNA); **Cannabinoid receptors** | Decreased (lateral caudate-putamen);  No alteration (medial caudate-putamen) |  | No alteration in agonist (WIN 55,212-2) stimulated [^35^S]GTPγS binding (lateral caudate-putamen); Decreased cannabinoid receptors mRNA levels (lateral and medial caudate-putamen) | (Romero et al., 1998) | |
|  |  | Human (18-45 and 45-70 years) | N-[2-(3-cyano-phenyl)-3-(4-(2-[^18^F]fluor-  ethoxy)phenyl)-1-methylpropyl]-2-(5-methyl-2-pyridyloxy)-2- methylproponamide binding (PET); **CB1** |  | Increased (women’s putamen);  No alteration (men’s putamen) |  | (Van Laere et al., 2008) | |
|  |  | Male Human (21-56 years) | [^11^C]OMAR binding (PET); **CB1** |  |  | No alteration in binding of an antagonist (putamen) | (Wong et al., 2010) | |
|  | Basal Ganglia - Globus Pallidus | Male Wistar rats (3 months and >2 years) | [^3^H]-WIN 55,212-2 binding (Autoradiography); **Cannabinoid receptors**  [^3^H]-WIN 55,212-2 stimulated [^35^S]GTPγS binding (Autoradiography); **Cannabinoid receptors** | No alteration |  | No alteration in agonist (WIN 55,212-2) stimulated [^35^S]GTPγS binding | (Romero et al., 1998) | |
|  |  | Male Human (21-56 years) | [^11^C]OMAR binding (PET); **CB1** |  |  | Decreased binding of an antagonist | (Wong et al., 2010) | |
|  | Basal Ganglia -Entopeduncular nucleus | Male Wistar rats (3 months and >2 years) | [^3^H]-WIN 55,212-2 binding (Autoradiography); **Cannabinoid receptors** | Decreased |  |  | (Romero et al., 1998) | |
|  | Cortex | Male Human (21-56 years) | [^11^C]OMAR binding (PET); **CB1** |  |  | No alteration in binding of an antagonist | (Wong et al., 2010) | |
|  | Cerebral cortex | Male Wistar rats (3 months and >2 years) | [^3^H]-WIN 55,212-2 binding (Autoradiography); **Cannabinoid receptors**  [^35^S]dATP labelled hybridization (mRNA); **Cannabinoid receptors** | Decreased (deep layer VI);  No alteration (superficial layer I) |  | No alteration in cannabinoid receptors mRNA levels | (Romero et al., 1998) | |
|  | Cerebral cortex - Frontal cortex | Human (*postmortem*) (22-73 years) | [^3^H]CP55940 binding (Autoradiography); **CB1**  ([^3^H]CP55940) stimulated [^35^S]GTPγS binding; **CB1** | Decreased |  | Decreased basal [^35^S]GTPγS binding;  No alteration in agonist ([^3^H] CP55940) stimulated [^35^S]GTPγS binding | (Mato and Pazos, 2004) | |
|  | Cerebral cortex - Frontal cortex – Orbitofrontal cortex | Human (18-45 and 45-70 years) | N-[2-(3-cyano-phenyl)-3-(4-(2-[^18^F]fluor-  ethoxy)phenyl)-1-methylpropyl]-2-(5-methyl-2-pyridyloxy)-2- methylproponamide binding (PET); **CB1** |  | Increased  (women); No alteration (men) |  | (Van Laere et al., 2008) | |
|  | Cerebral cortex - Frontal cortex – Lower lateral prefrontal cortex |  |  |  |  |  |  |  |
|  | Cerebral cortex -Occipital cortex |  |  |  |  |  |  |  |
|  | Cerebral cortex – Middle and inferior temporal cortex |  |  |  |  |  |  |  |
|  | Cerebral cortex – Temporal lobe - Entorhinal cortex |  |  |  |  |  |  |  |
|  | Anterior cingulate cortex |  |  |  |  |  |  |  |
|  | Parietotemporal cortex | Mice (6 and 15 months) | ([3R,5R]-5-((3-([^18^F]fluoromethoxy-d2)phenyl)-  3-((R)-1-phenyl-ethylamino)-1-(4-trifluoromethyl-phenyl)-pyrrolidin-  2-one) binding (PET); **CB1** |  | Increased |  | (Takkinen et al., 2018) | |
| Leukotriene Receptors | -------------------------- | | | | | | | |
| Metabotropic GABA Receptors | Cerebellum - Molecular layer | Fischer 344 rats (3 and 26 months) | [^3^H]GABA (Autoradiography); **GABA_B_** |  |  | No alteration in GABA_B_ receptor binding | (Milbrandt et al., 1994) | |
|  | Corpora Quadrigemina – Inferior Colliculus |  |  |  | Decreased |  |  |  |
|  | Hippocampus | Male Fischer 344 rats (6-22 months) | Western Blot; **GABA_B_R2** | No alteration |  |  | (McQuail et al., 2012) | |
|  | Cerebral cortex - Frontal cortex |  |  | Decreased |  |  |  |  |
|  | Sensory cortex | Human (*postmortem*) (34-89 years) | Western Blot; **GABA_B_R2** | No alteration |  |  | (Pandya et al., 2019) | |
|  | Motor cortex | Human (*postmortem*) (34-89 years) | Western Blot; **GABA_B_R2** | No alteration |  |  | (Pandya et al., 2019) | |
|  | Cerebellum | Human (*postmortem*) (34-89 years) | Western Blot; **GABA_B_R2** | No alteration |  |  | (Pandya et al., 2019) | |
|  | Cerebral cortex -Temporal cortex - Inferior temporal gyrus | Human (*postmortem*) (34-89 years) | Western Blot; **GABA_B_R2** | No alteration |  |  | (Pandya et al., 2019) | |
|  | Cerebral cortex -Temporal cortex - Medial temporal gyrus | Human (*postmortem*) (34-89 years) | Western Blot; **GABA_B_R2** | No alteration |  |  | (Pandya et al., 2019) | |
|  | Cerebral cortex -Temporal cortex - Superior temporal gyrus | Human (*postmortem*) (34-89 years) | Western Blot; **GABA_B_R2** | No alteration |  |  | (Pandya et al., 2019) | |
|  | Primary visual cortex | Rhesus Monkeys *(Macaca mulatta)* (8-15 years and 23-26 years) | qPCR (mRNA); **GABA_B_R2** |  |  | Increased GABA_B2_ mRNA levels | (Liao et al., 2016) | |
|  | Cerebral cortex - Frontal cortex – Prefrontal cortex | Human *(postmortem)* | Baclofen stimulated [^35^S]GTPγS binding; **GABA_B_** |  |  | Decreased in agonist (baclofen) stimulated [^35^S]GTPγS binding | (González-Maeso et al., 2002) | |
| Metabotropic Glutamate Receptors | Cerebellum | Male Fischer 344 rats (3-25 months) | Western blot; **mGluR2/3**  ^35^S-dATP labeled hybridization (mRNA); **mGluR2; mGluR3** | Increased |  | No alteration in mGluR2 mRNA levels;  Increased mGluR3 mRNA levels | (Simonyi et al., 2005) | |
|  |  |  | Immunohistochemistry; **mGluR2/3** | No alteration |  |  | (Simonyi et al., 2005) | |
|  | Thalamus – Lateral dorsal nucleus | Male Fischer 344 rats (3-25 months) | Immunohistochemistry; **mGluR2/3** | Increased |  |  | (Simonyi et al., 2005) | |
|  |  |  | ^35^S-dATP labeled hybridization (mRNA); **mGluR7** |  |  | Decreased mGluR7 mRNA levels | (Simonyi et al., 2000) | |
|  | Thalamus – Ventral posterior lateral nucleus |  |  |  |  | No alteration in mGluR7 mRNA levels |  |  |
|  |  |  | Immunohistochemistry; **mGluR2/3** | Increased |  |  | (Simonyi et al., 2005) | |
|  | Thalamus – Thalamic reticular nucleus |  | Immunohistochemistry; **mGluR2/3**. ^35^S-dATP labeled hybridization (mRNA); **mGluR3** | Increased |  | Increased mGluR3 mRNA levels | (Simonyi et al., 2005) | |
|  | White matter – Internal capsule |  | ^35^S-dATP labeled hybridization (mRNA); **mGluR3** |  |  | Increased mGluR3 mRNA levels |  |  |
|  | Hippocampus |  | Western blot; **mGluR2/3** | Increased |  |  |  |  |
|  | Hippocampus – Dentate gyrus |  | ^35^S-dATP labeled hybridization (mRNA); **mGluR2** |  |  | Decreased mGluR2 mRNA levels (upper blade); No alteration (lower blade) |  |  |
|  |  |  | ^35^S-dATP labeled hybridization (mRNA); **mGluR3** |  |  | No alteration mGluR3 mRNA levels (upper blade); Increased (lower blade) |  |  |
|  |  |  | ^35^S-dATP labeled hybridization (mRNA); **mGluR7** |  |  | No alteration mGluR7mRNA levels (upper blade); Decreased (lower blade) | (Simonyi et al., 2000) | |
|  | Hippocampus – CornuAmmonis area 1 and 3 |  |  | Increased (CA3) |  | Decreased mGluR7mRNA levels (CA1);  No alteration (CA3) |  |  |
|  |  |  | Immunohistochemistry; **mGluR2/3** | No alteration (CA1) |  |  | (Simonyi et al., 2005) | |
|  | Basal Ganglia – Striatum – Caudate and Putamen | Male Fischer 344 rats (3-25 months) | Western blot; Immunohistochemistry; **mGluR2/3**  ^35^S-dATP labeled hybridization (mRNA); **mGluR2; mGluR3** | Increased |  | Increased mGluR2 mRNA levels;  No alteration in mGluR3 mRNA levels | (Simonyi et al., 2005) | |
|  |  |  | ^35^S-dATP labeled hybridization (mRNA); **mGluR7** |  |  | No alteration in mGluR7 mRNA levels | (Simonyi et al., 2000) | |
|  | Basal Ganglia – Striatum – Nucleus accumbens |  | ^35^S-dATP labeled hybridization (mRNA); **mGluR3** |  |  | Increased mGluR3 mRNA levels (core);  No alteration (shell) | (Simonyi et al., 2005) | |
|  |  |  | ^35^S-dATP labeled hybridization (mRNA); **mGluR7** |  |  | No alteration in mGluR7 mRNA levels | (Simonyi et al., 2000) | |
|  | Piriform cortex |  |  |  |  | Decreased mGluR7 mRNA levels |  |  |
|  | Cerebral cortex |  | Western blot; **mGluR2/3** | Increased |  |  | (Simonyi et al., 2005) | |
|  | Cerebral cortex - Frontal cortex |  | Immunohistochemistry; **mGluR2/3** ^35^S-dATP labeled hybridization (mRNA); **mGluR2; mGluR3** | Increased |  | No alteration in mGluR2 and mGluR3 mRNA levels |  |  |
|  |  |  | ^35^S-dATP labeled hybridization (mRNA); **mGluR7** |  |  | Decreased mGluR7 mRNA levels | (Simonyi et al., 2000) | |
|  | Cerebral cortex - Parietal cortex |  |  |  |  | Decreased mGluR7 mRNA levels |  |  |
|  |  |  | ^35^S-dATP labeled hybridization (mRNA); **mGluR2; mGluR3** |  |  | No alteration in mGluR2 and mGluR3 mRNA levels | (Simonyi et al., 2005) | |
|  | Cerebral cortex -Occipital cortex | Male Fischer 344 rats (3-25 months) | ^35^S-dATP labeled hybridization (mRNA); **mGluR2; mGluR3** |  |  | No alteration in mGluR2 mRNA levels;  Increased mGluR3 mRNA levels | (Simonyi et al., 2005) | |
|  |  |  | ^35^S-dATP labeled hybridization (mRNA); **mGluR7** |  |  | Decreased mGluR7 mRNA levels | (Simonyi et al., 2000) | |
|  | Cerebral cortex -Temporal cortex | Male Fischer 344 rats (3-25 months) |  |  |  | Decreased mGluR7 mRNA levels |  |  |
|  |  |  | ^35^S-dATP labeled hybridization (mRNA); **mGluR2; mGluR3** |  |  | No alteration in mGluR2 and mGluR3 mRNA levels | (Simonyi et al., 2005) | |
|  | Cerebral cortex – Temporal lobe - Entorhinal cortex |  |  |  |  | No alteration in mGluR2 and mGluR3 mRNA levels |  |  |
|  |  |  | ^35^S-dATP labeled hybridization (mRNA); **mGluR7** |  |  | No alteration in mGluR7 mRNA levels | (Simonyi et al., 2000) | |
|  | Corpus callosum |  | Immunohistochemistry; **mGluR2/3**  ^35^S-dATP labeled hybridization (mRNA); **mGluR3** | Increased |  | Increased mGluR3 mRNA levels | (Simonyi et al., 2005) | |
|  | Periaqueductal area - Central gray |  |  | Increased |  | Increased mGluR3 mRNA levels |  |  |
|  |  |  | ^35^S-dATP labeled hybridization (mRNA); **mGluR7** |  |  | No alteration in mGluR7 mRNA levels | (Simonyi et al., 2000) | |

**References**

Antonini A, L.K., Antonini, A., Leenders, K.L., Reist, H., Thomann, R., Beer, H.F., Locher, J., 1993. Effect of age on D2 dopamine receptors in normal human brain measured by positron emission tomography and 11C-raclopride. Arch. Neurol. 50, 474–80. https://doi.org/10.1001/archneur.1993.00540050026010

Araki, T., Kato, H., Shuto, K., Fujiwara, T., Itoyama, Y., 1997. Effect of aging on dopaminergic receptors and uptake sites in the rat brain studied by receptor autoradiography. J. Neurol. Sci. 148, 131–7. https://doi.org/10.1016/S0022-510X(96)05343-9

Arranz, B., Eriksson, A., Mellerup, E., Plenge, P., Marcusson, J., 1993. Effect of aging in human cortical pre- and postsynaptic serotonin binding sites. Brain Res. 620, 163–6.

Bigham, M.H., Lidow, M.S., 1995. Adrenergic and serotonergic receptors in aged monkey neocortex. Neurobiol. Aging 16, 91–104.

Blake, M.J., Appel, N.M., Joseph, J.A., Stagg, C.A., Anson, M., de Souza, E.B., Roth, G.S., 1991. Muscarinic acetylcholine receptor subtype mRNA expression and ligand binding in the aged rat forebrain. Neurobiol. Aging 12, 193–9.

Burnet, P.W., Eastwood, S.L., Harrison, P.J., 1994. Detection and quantitation of 5-HT1A and 5-HT2A receptor mRNAs in human hippocampus using a reverse transcriptase-polymerase chain reaction (RT-PCR) technique and their correlation with binding site densities and age. Neurosci. Lett. 178, 85–9.

Canas, P.M., Duarte, J.M.N., Rodrigues, R.J., Köfalvi, A., Cunha, R.A., 2009. Modification upon aging of the density of presynaptic modulation systems in the hippocampus. Neurobiol. Aging 30, 1877–84. https://doi.org/10.1016/j.neurobiolaging.2008.01.003

Dang, L.C., Castrellon, J.J., Perkins, S.F., Le, N.T., Cowan, R.L., Zald, D.H., Samanez-Larkin, G.R., 2017. Reduced effects of age on dopamine D2 receptor levels in physically active adults. Neuroimage 148, 123–129. https://doi.org/10.1016/j.neuroimage.2017.01.018

De Keyser, J., Ebinger, G., Vauquelin, G., 1990. Age-related changes in the human nigrostriatal dopaminergic system. Ann. Neurol. 27, 157–61. https://doi.org/10.1002/ana.410270210

Dillon, K.A., Gross-Isseroff, R., Israeli, M., Biegon, A., 1991. Autoradiographic analysis of serotonin 5-HT1A receptor binding in the human brain postmortem: effects of age and alcohol. Brain Res. 554, 56–64.

Duncan, M.J., Hensler, J.G., 2002. Aging alters in a region-specific manner serotonin transporter sites and 5-HT(1A) receptor-G protein interactions in hamster brain. Neuropharmacology 43, 36–44.

González-Maeso, J., Torre, I., Rodríguez-Puertas, R., García-Sevilla, J.A., Guimón, J., Meana, J.J., 2002. Effects of Age, Postmortem Delay and Storage Time on Receptor-mediated Activation of G-proteins in Human Brain. Neuropsychopharmacology 26, 468–478. https://doi.org/10.1016/S0893-133X(01)00342-6

Hamilton, C.A., Howe, C.A., Reid, J.L., 1984. Changes in brain alpha-adrenoceptors with increasing age in rabbits. Brain Res. 322, 177–9.

Han, Z., Kuyatt, B.L., Kochman, K.A., DeSouza, E.B., Roth, G.S., 1989. Effect of aging on concentrations of D2-receptor-containing neurons in the rat striatum. Brain Res. 498, 299–307.

Henry, J.M., Filburn, C.R., Joseph, J.A., Roth, G.S., 1986. Effect of aging on striatal dopamine receptor subtypes in Wistar rats. Neurobiol. Aging 7, 357–61.

Hess, G.D., Joseph, J.A., Roth, G.S., 1981. Effect of age on sensitivity to pain and brain opiate receptors. Neurobiol. Aging 2, 49–55.

Hiller, J.M., Fan, L.Q., Simon, E.J., 1992. Age-related changes in kappa opioid receptors in the guinea-pig brain: a quantitative autoradiographic study. Neuroscience 50, 663–73.

Huguet, F., Drieu, K., Piriou, A., 1994. Decreased cerebral 5-HT1A receptors during ageing: reversal by Ginkgo biloba extract (EGb 761). J. Pharm. Pharmacol. 46, 316–8.

Inoue, M., Suhara, T., Sudo, Y., Okubo, Y., Yasuno, F., Kishimoto, T., Yoshikawa, K., Tanada, S., 2001. Age-related reduction of extrastriatal dopamine D2 receptor measured by PET. Life Sci. 69, 1079–1084. https://doi.org/10.1016/S0024-3205(01)01205-X

Ishibashi, K., Ishii, K., Oda, K., Kawasaki, K., Mizusawa, H., Ishiwata, K., 2009. Regional analysis of age-related decline in dopamine transporters and dopamine D2-like receptors in human striatum. Synapse 63, 282–90. https://doi.org/10.1002/syn.20603

Joyce, J.N., Loeschen, S.K., Sapp, D.W., Marshall, J.F., 1986. Age-related regional loss of caudate-putamen dopamine receptors revealed by quantitative autoradiography. Brain Res. 378, 158–63.

Kaasinen, V., Vilkman, H., Hietala, J., Någren, K., Helenius, H., Olsson, H., Farde, L., Rinne, J., 2000. Age-related dopamine D2/D3 receptor loss in extrastriatal regions of the human brain. Neurobiol. Aging 21, 683–8.

Lai, H., Bowden, D.M., Horita, A., 1987. Age-related decreases in dopamine receptors in the caudate nucleus and putamen of the rhesus monkey (Macaca mulatta). Neurobiol. Aging 8, 45–9.

Laquerriere, A., Leroux, P., Bodenant, C., Gonzalez, B., Tayot, J., Vaudry, H., 1994. Quantitative autoradiographic study of somatostatin receptors in the adult human cerebellum. Neuroscience 62, 1147–54.

Lee, H.J., Clagett-Dame, M., Heideman, W., Weiler, M.S., 1994. The effect of age on muscarinic receptor transcripts in rat brain. Neurosci. Lett. 174, 205–8.

Liao, C., Han, Q., Ma, Y., Su, B., 2016. Age-related gene expression change of GABAergic system in visual cortex of rhesus macaque. Gene 590, 227–233. https://doi.org/10.1016/j.gene.2016.05.010

Maggi, R., Limonta, P., Dondi, D., Martini, L., Piva, F., 1989. Distribution of kappa opioid receptors in the brain of young and old male rats. Life Sci. 45, 2085–92.

Mailleux, P., Vanderhaeghen, J.J., 1992. Age-related loss of cannabinoid receptor binding sites and mRNA in the rat striatum. Neurosci. Lett. 147, 179–81.

Mato, S., Pazos, A., 2004. Influence of age, postmortem delay and freezing storage period on cannabinoid receptor density and functionality in human brain. Neuropharmacology 46, 716–726. https://doi.org/10.1016/j.neuropharm.2003.11.004

Matuskey, D., Pittman, B., Planeta-Wilson, B., Walderhaug, E., Henry, S., Gallezot, J.-D., Nabulsi, N., Ding, Y.-S., Bhagwagar, Z., Malison, R., Carson, R.E., Neumeister, A., 2012. Age effects on serotonin receptor 1B as assessed by PET. J. Nucl. Med. 53, 1411–4. https://doi.org/10.2967/jnumed.112.103598

Matuskey, D., Worhunksy, P., Correa, E., Pittman, B., Gallezot, J.-D., Nabulsi, N., Ropchan, J., Sreeram, V., Gudepu, R., Gaiser, E., Cosgrove, K., Ding, Y.-S., Potenza, M.N., Huang, Y., Malison, R.T., Carson, R.E., 2016. Age-related changes in binding of the D2/3 receptor radioligand [(11)C](+)PHNO in healthy volunteers. Neuroimage 130, 241–247. https://doi.org/10.1016/j.neuroimage.2016.02.002

McQuail, J.A., Bañuelos, C., LaSarge, C.L., Nicolle, M.M., Bizon, J.L., 2012. GABAB receptor GTP-binding is decreased in the prefrontal cortex but not the hippocampus of aged rats. Neurobiol. Aging 33, 1124.e1-1124.e12. https://doi.org/10.1016/j.neurobiolaging.2011.11.011

Meltzer, C.C., Drevets, W.C., Price, J.C., Mathis, C.A., Lopresti, B., Greer, P.J., Villemagne, V.L., Holt, D., Mason, N.S., Houck, P.R., Reynolds, C.F., DeKosky, S.T., 2001. Gender-specific aging effects on the serotonin 1A receptor. Brain Res. 895, 9–17. https://doi.org/10.1016/S0006-8993(00)03211-X

Mesco, E.R., Joseph, J.A., Blake, M.J., Roth, G.S., 1991. Loss of D2 receptors during aging is partially due to decreased levels of mRNA. Brain Res. 545, 355–7.

Messing, R.B., Vasquez, B.J., Samaniego, B., Jensen, R.A., Martinez, J.L., McGaugh, J.L., 1981. Alterations in dihydromorphine binding in cerebral hemispheres of aged male rats. J. Neurochem. 36, 784–7.

Milbrandt, J.C., Albin, R.L., Caspary, D.M., 1994. Age-related decrease in GABAB receptor binding in the Fischer 344 rat inferior colliculus. Neurobiol. Aging 15, 699–703.

Morgan, D.G., Marcusson, J.O., Nyberg, P., Wester, P., Winblad, B., Gordon, M.N., Finch, C.E., 1987. Divergent changes in D-1 and D-2 dopamine binding sites in human brain during aging. Neurobiol. Aging 8, 195–201.

Nakajima, S., Caravaggio, F., Boileau, I., Chung, J.K., Plitman, E., Gerretsen, P., Wilson, A.A., Houle, S., Mamo, D.C., Graff-Guerrero, A., 2015. Lack of age-dependent decrease in dopamine D3 receptor availability: a [(11)C]-(+)-PHNO and [(11)C]-raclopride positron emission tomography study. J. Cereb. Blood Flow Metab. 35, 1812–8. https://doi.org/10.1038/jcbfm.2015.129

Nieves-Martinez, E., Haynes, K., Childers, S.R., Sonntag, W.E., Nicolle, M.M., 2012. Muscarinic receptor/G-protein coupling is reduced in the dorsomedial striatum of cognitively impaired aged rats. Behav. Brain Res. 227, 258–64. https://doi.org/10.1016/j.bbr.2011.10.048

Nomura, Y., Kitamura, Y., Kawai, M., Segawa, T., 1986. Alpha 2-adrenoceptor-GTP binding regulatory protein-adenylate cyclase system in cerebral cortical membranes of adult and senescent rats. Brain Res. 379, 118–24.

Nordberg, A., Alafuzoff, I., Winblad, B., 1992. Nicotinic and muscarinic subtypes in the human brain: changes with aging and dementia. J. Neurosci. Res. 31, 103–11. https://doi.org/10.1002/jnr.490310115

O’Boyle, K.M., Waddington, J.L., 1984. Loss of rat striatal dopamine receptors with ageing is selective for D-2 but not D-1 sites: association with increased non-specific binding of the D-1 ligand [3H]piflutixol. Eur. J. Pharmacol. 105, 171–4.

Palego, L., Marazziti, D., Rossi, A., Giannaccini, G., Naccarato, A.G., Lucacchini, A., Cassano, G.B., 1997. Apparent absence of aging and gender effects on serotonin 1A receptors in human neocortex and hippocampus. Brain Res. 758, 26–32.

Pandya, M., Palpagama, T.H., Turner, C., Waldvogel, H.J., Faull, R.L., Kwakowsky, A., 2019. Sex- and age-related changes in GABA signaling components in the human cortex. Biol. Sex Differ. 10, 5. https://doi.org/10.1186/s13293-018-0214-6

Parsey, R. V, Oquendo, M.A., Simpson, N.R., Ogden, R.T., Van Heertum, R., Arango, V., Mann, J.J., 2002. Effects of sex, age, and aggressive traits in man on brain serotonin 5-HT1A receptor binding potential measured by PET using [C-11]WAY-100635. Brain Res. 954, 173–82.

Pascual, J., del Arco, C., González, A.M., Díaz, A., del Olmo, E., Pazos, A., 1991. Regionally specific age-dependent decline in alpha 2-adrenoceptors: an autoradiographic study in human brain. Neurosci. Lett. 133, 279–83.

Piva, F., Maggi, R., Limonta, P., Dondi, D., Martini, L., 1987. Decrease of mu opioid receptors in the brain and in the hypothalamus of the aged male rat. Life Sci. 40, 391–8.

Pohjalainen, T., Rinne, J.O., Någren, K., Syvälahti, E., Hietala, J., 1998. Sex differences in the striatal dopamine D2 receptor binding characteristics in vivo. Am. J. Psychiatry 155, 768–73. https://doi.org/10.1176/ajp.155.6.768

Reed, D.K., Korytko, A.I., Hipkin, R.W., Wehrenberg, W.B., Schonbrunn, A., Cuttler, L., 1999. Pituitary somatostatin receptor (sst)1-5 expression during rat development: age-dependent expression of sst2. Endocrinology 140, 4739–44. https://doi.org/10.1210/endo.140.10.7033

Ricci, A., Mammola, C.L., Vega, J.A., Zaccheo, D., Amenta, F., 1996. Density and pattern of dopamine D2-like receptors in the cerebellar cortex of aged rats. Neurobiol. Aging 17, 45–52.

Rinne, J.O., 1987. Muscarinic and dopaminergic receptors in the aging human brain. Brain Res. 404, 162–8.

Rinne, J.O., Hietala, J., Ruotsalainen, U., Säkö, E., Laihinen, A., Någren, K., Lehikoinen, P., Oikonen, V., Syvälahti, E., 1993. Decrease in human striatal dopamine D2 receptor density with age: a PET study with [11C]raclopride. J. Cereb. blood flow Metab. 13, 310–314. https://doi.org/10.1038/jcbfm.1993.39

Rinne, J.O., Lönnberg, P., Marjamäki, P., 1990. Age-dependent decline in human brain dopamine D1 and D2 receptors. Brain Res. 508, 349–52.

Romero, J., Berrendero, F., Garcia-Gil, L., de la Cruz, P., Ramos, J.A., Fernández-Ruiz, J.J., 1998. Loss of cannabinoid receptor binding and messenger RNA levels and cannabinoid agonist-stimulated [35S]guanylyl-5’O-(thio)-triphosphate binding in the basal ganglia of aged rats. Neuroscience 84, 1075–83.

Seeman, P., Bzowej, N.H., Guan, H.C., Bergeron, C., Becker, L.E., Reynolds, G.P., Bird, E.D., Riederer, P., Jellinger, K., Watanabe, S., 1987. Human brain dopamine receptors in children and aging adults. Synapse 1, 399–404. https://doi.org/10.1002/syn.890010503

Shimokawa, I., Yanagihara, K., Higami, Y., Okimoto, T., Tomita, M., Ikeda, T., Lee, S., 2000. Effects of aging and dietary restriction on mRNA levels of receptors for growth hormone-releasing hormone and somatostatin in the rat pituitary. J. Gerontol. A. Biol. Sci. Med. Sci. 55, B274-9.

Simonyi, A., Miller, L.A., Sun, G.Y., 2000. Region-specific decline in the expression of metabotropic glutamate receptor 7 mRNA in rat brain during aging. Brain Res. Mol. Brain Res. 82, 101–6.

Simonyi, A., Ngomba, R.T., Storto, M., Catania, M. V., Miller, L.A., Youngs, B., DiGiorgi-Gerevini, V., Nicoletti, F., Sun, G.Y., 2005. Expression of groups I and II metabotropic glutamate receptors in the rat brain during aging. Brain Res. 1043, 95–106. https://doi.org/10.1016/j.brainres.2005.02.046

Sirohi, S., Walker, B.M., 2015. Maturational alterations in constitutive activity of medial prefrontal cortex kappa-opioid receptors in Wistar rats. J. Neurochem. 135, 659–65. https://doi.org/10.1111/jnc.13279

Sirvio, J., Jolkkonen, J., Pitkanen, A., Riekkinen, P.J., 1987. Age Dependence of Somatostatin Levels and Somatostatin Binding in the Rat Brain. Comp. Biochem. Physiol. 87, 355–357.

Takkinen, J.S., López-Picón, F.R., Kirjavainen, A.K., Pihlaja, R., Snellman, A., Ishizu, T., Löyttyniemi, E., Solin, O., Rinne, J.O., Haaparanta-Solin, M., 2018. [18F]FMPEP-d2 PET imaging shows age- and genotype-dependent impairments in the availability of cannabinoid receptor 1 in a mouse model of Alzheimer’s disease. Neurobiol. Aging 69, 199–208. https://doi.org/10.1016/j.neurobiolaging.2018.05.013

Tauscher, J., Verhoeff, N.P.L., Christensen, B.K., Hussey, D., Meyer, J.H., Kecojevic, A., Javanmard, M., Kasper, S., Kapur, S., 2001. Serotonin 5-HT1A Receptor Binding Potential Declines with Age as Measured by [11C]WAY-100635 and PET. Neuropsychopharmacology 24, 522–530. https://doi.org/10.1016/S0893-133X(00)00227-X

Tayebati, S.K., Di Tullio, M.A., Amenta, F., 2004. Age-related changes of muscarinic cholinergic receptor subtypes in the striatum of Fisher 344 rats. Exp. Gerontol. 39, 217–23. https://doi.org/10.1016/j.exger.2003.10.016

Tayebati, S.K., Vitali, D., Scordella, S., Amenta, F., 2001. Muscarinic cholinergic receptors subtypes in rat cerebellar cortex: light microscope autoradiography of age-related changes. Brain Res. 889, 256–9.

Thal, L.J., Horowitz, S.G., Dvorkin, B., Makman, M.H., 1980. Evidence for loss of brain [3H]spiroperidil and [3H]ADTN binding sites in rabbit brain with aging. Brain Res. 192, 185–194. https://doi.org/10.1016/0006-8993(80)91018-5

Valerio, A., Belloni, M., Gorno, M.L., Tinti, C., Memo, M., Spano, P., 1994. Dopamine D2, D3, and D4 receptor mRNA levels in rat brain and pituitary during aging. Neurobiol. Aging 15, 713–9.

Van Laere, K., Goffin, K., Casteels, C., Dupont, P., Mortelmans, L., de Hoon, J., Bormans, G., 2008. Gender-dependent increases with healthy aging of the human cerebral cannabinoid-type 1 receptor binding using [(18)F]MK-9470 PET. Neuroimage 39, 1533–41. https://doi.org/10.1016/j.neuroimage.2007.10.053

Villar-Cheda, B., Dominguez-Meijide, A., Valenzuela, R., Granado, N., Moratalla, R., Labandeira-Garcia, J.L., 2014. Aging-related dysregulation of dopamine and angiotensin receptor interaction. Neurobiol. Aging 35, 1726–38. https://doi.org/10.1016/j.neurobiolaging.2014.01.017

Villar-Cheda, B., Valenzuela, R., Rodriguez-Perez, A.I., Guerra, M.J., Labandeira-Garcia, J.L., 2012. Aging-related changes in the nigral angiotensin system enhances proinflammatory and pro-oxidative markers and 6-OHDA-induced dopaminergic degeneration. Neurobiol. Aging 33, 204.e1-11. https://doi.org/10.1016/j.neurobiolaging.2010.08.006

Wallace, D.R., Booze, R.M., 1996. Dopamine D3 receptor density elevation in aged Fischer-344 x Brown-Norway (F1) rats. Eur. J. Pharmacol. 308, 283–5.

Wang, G.J., Volkow, N.D., Logan, J., Fowler, J.S., Schlyer, D., MacGregor, R.R., Hitzemann, R.J., Gur, R.C., Wolf, A.P., 1995. Evaluation of age-related changes in serotonin 5-HT2 and dopamine D2 receptor availability in healthy human subjects. Life Sci. 56, PL249-53.

Weiss, B., Chen, J.F., Zhang, S., Zhou, L.W., 1992. Developmental and age-related changes in the D2 dopamine receptor mRNA subtypes in rat brain. Neurochem. Int. 20 Suppl, 49S–58S.

Wong, D.F., Kuwabara, H., Horti, A.G., Raymont, V., Brasic, J., Guevara, M., Ye, W., Dannals, R.F., Ravert, H.T., Nandi, A., Rahmim, A., Ming, J.E., Grachev, I., Roy, C., Cascella, N., 2010. Quantification of cerebral cannabinoid receptors subtype 1 (CB1) in healthy subjects and schizophrenia by the novel PET radioligand [11C]OMAR. Neuroimage 52, 1505–1513. https://doi.org/10.1016/j.neuroimage.2010.04.034

Wong, D.F., Wagner Jr., H.N., Dannals, R.F., Links, J.M., Frost, J.J., Ravert, H.T., Wilson, A.A., Rosenbaum, A.E., Gjedde, A., Douglass, K.H., Petronis, J.D., Folstein, M.F., Toung, J.K.T., Burns, H.D., Kuhar, M.J., 1984. Effects of age on dopamine and serotonin receptors measured by positron tomography in the living human brain. Science (80-. ). 226, 1393–1396.

Yew, D.T., Yeung, L.-Y., Wai, M.S.-M., Mak, Y.T., 2009. 5-HT 1A and 2A receptor positive cells in the cerebella of mice and human and their decline during aging. Microsc. Res. Tech. 72, 684–9. https://doi.org/10.1002/jemt.20717
